# Effect of sensory-motor intervention associated with skin-to-skin contact on neuromotor and clinical outcomes of preterm newborns: A randomized controlled trial

**DOI:** 10.1371/journal.pone.0332269

**Published:** 2025-09-12

**Authors:** Mariane de Oliveira Nunes Reco, Andressa Lagoa Nascimento França, Priscila Rímoli de Almeida, Daniele Soares-Marangoni

**Affiliations:** 1 Graduate Program in Health and Development, Faculty of Medicine, Federal University of Mato Grosso do Sul, Cidade Universitária, Campo Grande, MS, Brazil; 2 University Hospital Maria Aparecida Pedrossian, Federal University of Mato Grosso do Sul, Campo Grande, MS, Brazil; 3 Hospital Santa Casa ABC, Campo Grande, MS, Brazil; 4 Regional Hospital of Mato Grosso do Sul, Av. Eng. Lutero Lopes, Campo Grande, MS, Brazil; 5 Institute of Health, Physical Therapy Section, Federal University of Mato Grosso do Sul, Cidade Universitária, s/n, Campo Grande, MS, Brazil; Muhimbili University of Health and Allied Sciences, TANZANIA, UNITED REPUBLIC OF

## Abstract

**Objective:**

To examine the effects of a physical therapy sensory-motor intervention combined with kangaroo skin-to-skin contact in clinically stable preterm newborns.

**Design, setting, and participants:**

This randomized controlled trial included two parallel and balanced groups. Thirty-four preterm newborns (≤34 weeks gestation and low birth weight) who were hospitalized in a neonatal intermediate care unit were randomly assigned to either the experimental (EG) or control (CG) group. Only newborns with a poor repertoire, as determined by the Prechtl General Movements Assessment (GMA), were included.

**Interventions:**

The intervention consisted of 10 sessions of 15-minute sensory-motor physical therapy followed by 60 minutes of skin-to-skin contact (EG), or 60 minutes of skin-to-skin contact only (CG).

**Outcome measures:**

The primary outcome was the quality of general movements (GMs), assessed by the GMA during the writhing movements (WM) and fidgety movements (FM) phases. Secondary outcomes included weight gain, posture and muscle tone, behavioral state, length of hospital stay, and establishment and maintenance of breastfeeding. Vital signs and signs of respiratory distress were also monitored.

**Results:**

Newborns gained weight, showed no signs of respiratory distress, and maintained stable vital signs during the interventions. Both groups exhibited similar proportions of normal and abnormal GMs at both the WM and FM phases. However, the EG group showed improved scores for popliteal angle and leg recoil after the intervention compared to the control group.

**Conclusions:**

The neonatal physical therapy intervention was a clinically safe technique for stable preterm newborns. This technique may be recommended to promote the development of physiological flexor tone in the lower limbs, but it does not appear to provide advantages in improving the quality of GMs compared to skin-to-skin contact. Due to the non-confirmatory findings, this study should be considered a pilot.

**Trial registration:**

REBEC identifier RBR-4wx7wp. Trial registered in the Brazilian Clinical Trials Registry (ReBec). ReBec is a Primary Registry in the WHO Registry Network. Trial nº. RBR-5n82tv. *URL:* http://www.ensaiosclinicos.gov.br/rg/RBR-5n82tv/

## Introduction

Neonatal care plays a pivotal role in shaping the developmental outcomes of at-risk infants, particularly those born prematurely (< 37 weeks of gestation). Given the critical period of neuroplasticity in the early stages of life, there is growing interest in investigating interventions starting in neonatal unit care that may improve outcomes for these infants [1].

Sensory-motor stimulation is a widely endorsed early intervention in neonatal care. It includes strategies designed to enhance neuropsychomotor development by providing multisensory stimuli (e.g., auditory, tactile, visual, vestibular) tailored to the infant’s functional development, gestational age, and weight [2]. Evidence suggests that such stimuli can improve various developmental outcomes, including behavioral organization, muscle tone, and weight gain, and can even shorten the length of hospitalization [1–3].

Gentle tactile interactions, particularly those provided by touching, can potentially improve the newborn’s experiences [2,3]. Affective touch has been linked to the activation of specific mechanoreceptors involved in modulating emotional and stress-regulatory pathways, which can potentially improve neonatal outcomes [4–6]. In fact, skin-to-skin contact—a core element of kangaroo care involving direct affective touch between the newborn’s skin and the caregiver’s bare skin—is recognized for multiple neonatal benefits. These include physiological stabilization, enhanced bonding, improved breastfeeding outcomes, better sleep and weight gain, reduced length of hospitalization, and overall improvements in infant health [7–12]. Hence, skin-to-skin contact has become a global standard of neonatal care [13].

Interventions that encourage the newborn’s active movement and enhance mobility are also aligned with current recommendations, particularly in supporting neuromotor outcomes in at-risk infants [1,14]. Preterm infants often struggle with postural organization due to maturation-related hypotonia, which may lead to extensor muscle retractions, motor delays, and a reduced quality of spontaneous movements [14–16]. Interventions that promote the maturation of a newborn’s flexor chain may stimulate the development of better body organization and, perhaps, enhance the quality of movements [17].

Despite the positive and potential effects of interventions for newborns, few studies have investigated their impact on the quality of early spontaneous movements. A particular type of spontaneous movement, the so-called general movements (GMs), have been recognized as a reliable biomarker of brain function [17–20]. These movements, involving variable activity of all body parts, are present from early fetal life and gradually disappear by 16–20 weeks post-term as intentional movements begin to predominate. GMs are generated endogenously by central pattern generators (CPG) and modulated by supraspinal projections and sensory feedback. Hence, the quality of GMs expresses the young nervous system functioning [18,21].

Randomized controlled trials have provided mixed results on the effects of early interventions on GMs [22–24]. A parent-administered motor intervention before term age did not affect the quality of GMs at 3–4 months post-term compared to standard care [23]. However, a daily 90-minute structured multisensory intervention until discharge was effective in reducing the proportion of poor repertoire GMs compared to standard care [24]. When a multimodal intervention was continued by parents at home after hospital discharge, the GMs rate was significantly higher compared to controls [22]. Sensory-motor stimulation, which encourages tailored sensory experiences for newborns, is thought to support the development of neural pathways [25], which could ultimately improve GMs.

Although there is a growing body of literature that recommends sensory-motor interventions in neonatal care [1,2], the specific effects of these practices on sensitive markers of neurological function, such as GMs, remain underexplored. Additionally, given the contrasting results of the existing literature on this topic [22–24], further investigation is crucial to provide clinically relevant insights into the impact of early sensory-motor interventions on neuromotor outcomes in preterm newborns. Further research is also warranted, as physical therapists commonly employ a range of techniques with preterm newborns based on sensory-motor principles. This study aimed to examine the short- and medium-term effects of a sensory-motor physical therapy intervention combined with kangaroo skin-to-skin contact, in comparison to skin-to-skin contact alone, on the GMs of preterm newborns in a neonatal setting. Additionally, considering the relevance of strengthening the evidence base supporting early interventions in neonatal clinical practice, the secondary objectives included exploring the effects of the intervention on weight gain, posture and muscle tone, behavioral state, length of hospital stay, and the establishment and maintenance of breastfeeding. The intervention would be considered beneficial if infants in the treatment group demonstrated superior GMs quality or, secondarily, greater weight gain, improved scores for posture and muscle tone, reduced hospital stay, and higher proportions of sleep state and breastfeeding compared to controls. The findings provide new exploratory insights to shed light on clinical decision-making and future larger trials on the influence of sensory-motor experiences on early motor behaviors and clinical outcomes in preterm newborns.

## Materials and methods

### Study design and trial registration

**Fig 1:**
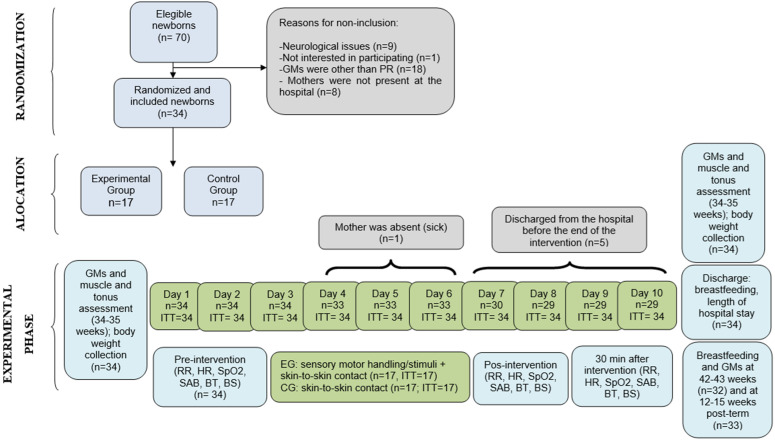
Flow diagram of the study. GMs, general movements; PR, poor repertoire; EG, experimental group; CG, control group; RR, respiratory rate; HC, heart rate; SpO2, oxygen saturation; SAB: Silverman-Andersen Bulletin; BS, behavioral state; BT, body temperature; ITT, intention-to-treat analysis.

This is a parallel-group trial with two arms. The study design details are presented in Fig 1. The trial is registered with the Brazilian Clinical Trials Registry (https://ensaiosclinicos.gov.br/rg/RBR-4wx7wp, accessed on March 13, 2024; registration date: September 27, 2019; latest update: October 18, 2023). The study protocol was published prospectively elsewhere [26]. Given the non-confirmatory nature of the findings and the clinical relevance of the information reported in this paper, we recommend that the overall study be regarded as a pilot trial.

Participant enrollment began on February 1, 2020. Recruitment was paused from March 2020 to June 2021 due to the COVID-19 pandemic restrictions. The last participant was assessed on June 19, 2024. The study was approved by the Ethics Committee of the Federal University of Mato Grosso do Sul (protocol code 01455818.2.0000.0021, date of approval: 19 December 2018).

### Participants and eligibility

Newborns were recruited from the Neonatal Intermediate Care Units (NInCUs) at the Maria Aparecida Pedrossian University Hospital and the Mato Grosso do Sul Regional Hospital, located in Campo Grande, Mato Grosso do Sul (Brazil). To estimate the minimum sample size, it was considered a difference of at least 50% between groups for the quality of GMs after the intervention using the chi-square test; additionally, it was taken as a reference that 26% of control preterm newborns with more than 34 weeks of postmenstrual age presented GMs with poor repertoire in the study by Ma et al. [19]. The suggested sample size was 15 participants per group (80% power, α = 5%). To account for potential losses to follow-up, two groups of 17 participants were included. Adequate recruitment was ensured by screening every newborn admitted to the NInCUs at the involved hospitals [26].

The inclusion criteria were a postmenstrual age of 34 weeks, more than 72 hours of postnatal life, admission to a NInCU, stable clinical condition, regardless of central or peripheral venous access, no need for mechanical ventilation, and GMs with a poor repertoire (see Section 2.6. Outcome Measures). Exclusion criteria included: congenital malformations, genetic syndromes, progressive conditions, orthopedic issues, grade III and/or IV peri-intraventricular hemorrhage, hyperbilirubinemia, congenital infections, ongoing infections (evidenced by changes in blood count or positive blood culture), and an Apgar score lower than 7 at five minutes. All newborns should spontaneously breathe room air, although the use of additional oxygen would not disqualify them. A written, informed legal consent form was signed by the parents prior to participation [26].

### Randomization

Randomization was carried out at the start of the project by creating two balanced groups using a Matlab routine with a random binary generator (experimental or control). Allocation was concealed from researchers and participants before the intervention began, using sequentially numbered, sealed, opaque envelopes. The newborn’s group assignment was determined by the order in which the envelopes were opened. Randomization, allocation, and concealment procedures were performed by a researcher who was blinded to the study objectives [26].

### Blinding

The primary outcome was video recorded and later assessed by two independent assessors who were blinded to the newborns’ group allocation. The medical team responsible for documenting some secondary outcomes in the medical records (body weight, length of hospital stay, and breastfeeding) was also blinded to the group assignments, as they were not involved in the study. Due to the nature of the intervention, the researcher administering it to the newborns could not be blinded. Similarly, the assessor evaluating the secondary outcomes posture and muscle tone, and behavioral state, could not be blinded in the NInCU over a 15-day program. Newborns were identified solely by their randomization number for data analysis, and data analysts were blinded to the group allocation as far as possible [26].

### Intervention

#### Physical therapy associated with Kangaroo skin-to-skin contact.

The physical therapy intervention consisted of a sensory-motor intervention conducted between feeding times, always at the same time each day. The experimental group received sensory-motor physical therapy combined with kangaroo skin-to-skin delivered in 10 sessions (one per day) across a 15-day period. Sessions were held once daily, excluding Sundays and days when the mother was unavailable. The intervention commenced when the newborn reached 34–35 weeks of postmenstrual age. By the end of the 15 days, each newborn had received ten 15-minute physical therapy sessions combined with 60 minutes of skin-to-skin contact and reached 36–37 weeks of postmenstrual age [26].

**Fig 2:**
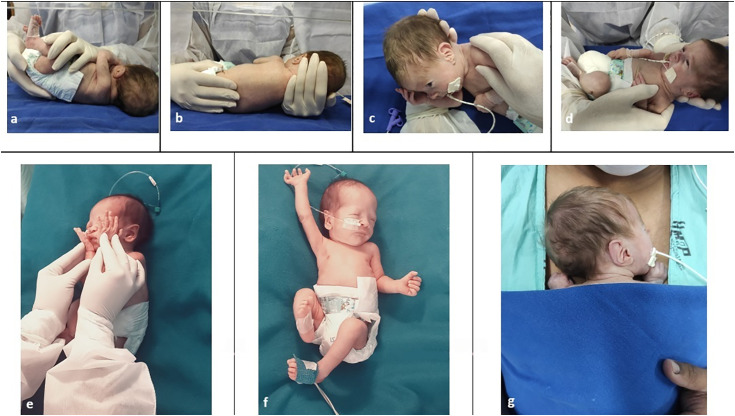
I) Passive mobilization and stretching, including lumbosacral (a), posterior (b) and cervical stretchings (c), and thoracohumeral dissociation (d); II) e) Sensory experience in head and face; III) f) Spontaneous general movements; IV) g) Kangaroo skin-to-skin contact. The individual in this manuscript has given written informed consent (as outlined in PLOS consent form) to publish these photographs.

The sensory-motor physical therapy followed techniques already implemented in the participating NInCUs and involved the following steps of sensory-motor handling and stimuli (Fig 2).

I) Passive mobilization and stretching: First, lumbosacral pomp was initiated using gentle caudal traction at the newborn’s pelvis. Posterior stretching was performed by applying passive traction along the newborn’s vertebral axis during expiration. Cervical stretching was then performed through lateral sliding of the newborn’s head and neck, combined with controlled shoulder mobilization. Finally, thoracohumeral dissociation involved circular mobilization of the shoulder region in the supine position (duration: 2 mins each handling, totaling 8 minutes) [26].
II) Sensory experience: Newborn in the supine position, head aligned, and trunk and lower limbs flexed, the therapist passively guided the newborn’s hands toward the parietal region, sliding the palms gently across his face—touching the cheeks, mouth, nose, and eyes—to provide controlled sensory stimulation (duration: 2 minutes) [26].
III) Spontaneous general movements: Newborn in the supine position in the incubator/crib was allowed to freely perform spontaneous motor activity (duration: 5 minutes) [26].
IV) Kangaroo skin-to-skin contact: Following spontaneous movements, the newborn was positioned for skin-to-skin contact in the kangaroo position on the parent’s thoracic region (duration: 60 minutes; pauses were provided when necessary, based on the newborn’s tolerance) [26].

Further details can be accessed in Reco et al. [26].

### Control intervention

The newborns in the control group underwent the same kangaroo skin-to-skin contact procedure as the experimental group, but without receiving the sensory-motor physical therapy. As such, the newborns were kept in the incubator or crib before the skin-to-skin contact with their parent [26].

The physical therapy and control interventions were carried out by the same researcher, a physical therapist with expertise in the described techniques, who was affiliated with one of the participating hospitals. If signs of sensory overload were observed—such as changes in skin color (e.g., pallor or perioral cyanosis), cardiorespiratory difficulties (e.g., bradycardia, irregular breathing, apnea, or irregular respiratory rate), state changes (e.g., crying, irritability, startles, hiccups, yawning, salivation), or withdrawal symptoms—the intervention was immediately interrupted. Physiological positioning and a firm touch were applied to help the newborn self-regulate. Completion of the intervention was defined as the delivery of at least 85% of the program. All newborns continued to receive standard hospital care [26].

### Outcome measures

#### Primary outcome.

The primary outcome was the quality of GMs assessed using Prechtl’s GMA. GMs involve coordinated movements of the upper and lower limbs, neck, and trunk, in a variable sequence. They are classified into age-specific categories: (a) fetal and preterm movements, occurring from the fetal period up to 37 weeks of postmenstrual age; (b) writhing movements (WMs), from 38 to 40 weeks of postmenstrual age until 8 weeks post-term; and (c) fidgety movements (FMs), which are observed from the 9^th^ to the 20^th^ post-term week. Normal WMs are characterized by small to moderate amplitudes, low to moderate velocity, occurring elliptically and giving the appearance of graceful contortions. Abnormal preterm and WMs are classified as: poor repertoire (PR), which consists of monotonous motor patterns with limited variability; cramped-synchronized (CS), when limb and trunk muscles contract and relax simultaneously without the fluidity of normal patterns; and chaotic (Ch), which involves high-amplitude movements that lack the smoothness and coordination of regular motor patterns. Normal FMs are small amplitude, moderate velocity movements of the limbs, trunk, and head, with variable acceleration and rotational movements of the hands and feet. Aberrant FMs include absent FMs (no or sporadic fidgety movements) and abnormally exaggerated FMs (marked by increased amplitude and velocity, and loss of continuity in the movements). Therefore, the GM quality categories include Normal, PR, CS, Ch, absent/sporadic FMs, and abnormal FMs [18,21].

During hospitalization, GMs were recorded using a cell phone following Prechtl’s GMA standards, with the newborns placed in the supine position, dressed only in a diaper or bodysuit, without pacifiers or exposure to physical/verbal interaction. Video recordings lasted 3–5 minutes and were conducted by the same researcher at two time points: (I) the day before the start of the interventions (34–35 weeks of postmenstrual age), and (II) one day after the interventions’ conclusion (day 16, around 36–37 weeks of postmenstrual age). After discharge, parents recorded GMs using a cell phone application at two additional time points: (I) 2–4 weeks post-term (WMs), and (II) 12–15 weeks post-term (FMs). Parents received prior instructions from the researcher on how to record GMs at home. Recordings were scheduled between feeding intervals (approximately 60–90 minutes) and were avoided on vaccination days [26].

The recorded GMs were assessed by two independent researchers certified by the GM Trust. In cases of disagreement, a third certified assessor was consulted to reach a consensus. The assessors were blinded to group allocation.

#### Secondary outcomes.

The secondary outcomes included body weight, posture and muscle tone, behavioral state, length of hospital stay, breastfeeding success, and breastfeeding maintenance [26].

Body Weight: The change in body weight between the day before day 1 and the day after day 10 of the intervention, collected from the medical team’s documentation on each day of the intervention. It is expected that preterm newborns experience a daily weight gain of approximately 15 grams [27].

Posture and Muscle Tone: Measured the day before day 1 and the day after day 10 of the intervention using an adapted neonatal neuromotor screening [28] based on Dubowitz et al. [29]. The screening starts with the observational assessment of the newborn’s posture, followed by assessment of arm recoil, leg recoil, scarf sign, popliteal angle, heel to ear, ankle dorsiflexion, head lag, ventral suspension, and head elevation. The tests are scored based on Dubowitz et al. [29], with lower scores indicating hypotonia and higher scores indicating hypertonia [28,29].

Behavioral State: Assessed on each day of the intervention immediately before, immediately after, and 30 minutes after the intervention, using the adapted Brazelton Neonatal Behavioral Assessment Scale. The scale categorizes the newborn’s behavior based on their current state, ranging from 1 to 6. State 1 corresponds to deep sleep, characterized by the absence of movement and regular breathing. State 2 reflects active or light sleep, with closed eyes and slight body movements. State 3 denotes a drowsy state, during which the newborn intermittently opens and closes the eyes. State 4 is the quiet alert state, marked by open eyes and minimal body activity. State 5 represents active alertness, with open eyes and pronounced body movements. State 6 is characterized by crying [30].

Length of Hospital Stay: The total number of days of hospitalization, collected from the medical records at discharge.

Breastfeeding Success: A dichotomous variable (yes/no) that reflects the primary guidelines outlined by the Brazilian Ministry of Health [31] for proper positioning and attachment during breastfeeding. The assessment was conducted by the nursery staff, noted in the medical records, and collected at hospital discharge.

Breastfeeding Maintenance: Defined as the continuation of exclusive breastfeeding (yes/no). Assessed through direct inquiry to the mothers at 2–4 weeks and at 12–15 weeks post-term, coinciding with GMs assessments.

#### Monitoring.

Each infant in the NInCU was continuously monitored by the hospital staff as part of routine clinical care. Information on vital signs (including respiratory rate, heart rate, body temperature, and peripheral oxygen saturation) and respiratory distress, measured using the Silverman–Anderson Bulletin (scores 0–2: normal; 3–4: mild respiratory distress; 5–6: moderate respiratory distress; 7–10: severe respiratory distress) [32], were recorded daily by the researcher from day 1 to day 10. These measurements were taken immediately before, immediately after, and 30 minutes after the intervention (in both experimental and control groups) [26].

### Data analysis

The analyses were performed using SPSS 23.0, with a significance level (α) set at 5% for all tests. All data were entered twice and audited by two researchers before being analyzed. Statistical analysis followed the intention-to-treat principle, meaning that all included newborns were analyzed. Data normality was tested through visual inspection of histograms and validated by the Shapiro-Wilk test. The baseline characteristics of both groups were described using means and standard deviations for continuous variables, and frequencies and percentages for categorical variables. To test for group differences (treatment effects) in categorical variables (quality of GMs, breastfeeding), chi-square tests were performed, with Cramér’s V or Phi used to assess effect size.

For comparisons between groups regarding continuous variables, such as body weight, posture and muscle tone, and length of hospitalization, two-sampled t-tests were conducted, with Cohen’s d used to describe effect size. For respiratory distress, the Mann-Whitney test was applied, with r calculated for effect size (r = z/ √N). For continuous variables measured across each intervention day (vital signs, behavioral state), adjusted mean differences between groups (estimates) and 95% confidence intervals were calculated using a mixed linear model (GLM), incorporating the interaction term: group × intervention day. For analysis purposes, the categories of behavioral state were merged as A for sleep state (states 1–3 – inactive sleep, active sleep, and drowsiness), B for alert state (states 4 and 5 – alert and active alert); and C for crying state (state 6).

## Results

A total data loss of 6.18% was observed across the intervention days in both groups, attributable to hospital discharge or the mother’s impossibility to perform kangaroo care (Fig 1). The experimental group received an average of 9.2 intervention days, corresponding to approximately 138 minutes of intervention per newborn throughout the 10 sessions.

### Sample characterization

A total of 34 preterm newborns were included, with a mean gestational age at birth of 31.30 ± 1.90 weeks, a birth weight of 1469.71 ± 367.19 grams, and a head circumference at birth of 28.69 ± 2.33 centimeters. The sample characteristics are detailed in Table 1.

**Table 1:** Sample characterization (n = 34).

Characteristics	EG (n = 17)	CG (n = 17)
**Sex** f (%)		
Female	9 (52.9)	8 (47.1)
Male	8 (47.1)	9 (52.9)
**Gestational Age** (M ± SD)	31.38 ± 0.37	31.22 ± 0.55
**Weight Birth** (M ± SD)	1440.29 ± 83.65	1499.12 ± 96.19
**Head Circumference** (M ± SD)	28.68 ± 0.47	28.70 ± 0.67
**Intrauterine Growth** f (%)		
AGA	15 (50.0)	15 (50.0)
SGA	2 (50.0)	2 (50.0)
**Apgar 1**^**st**^ **min** (M ± SD)	7.24 ± 0.30	7.82 ± 0.29
**Apgar 5**^**th**^ **min** (M ± SD)	8.53 ± 0.15	9.00 ± 0.17
US* f (%)		
Normal	16 (53.3)	14 (46.7)
PIVH I/II	1 (25.0)	3 (75.0)
**Maternal Age** (M ± SD)	28.88 ± 1.85	28.88 ± 1.20

EG, experimental group; CG, control group; PIVH, periventricular-intraventricular hemorrhage; M, mean; SD, standard deviation. f, frequency; AGA, adequate for gestational age; SGA, small for gestational age. *Brain tomography or magnetic resonance imaging were not performed or noted in medical records.

### Primary outcome

Regarding the quality of GMs, all newborns in the preterm GM phase exhibited PR GMs prior to the interventions, as this was an inclusion criterion. At the end of the interventions, no differences between the groups were observed in the quality of GMs at any time point. Both groups showed similar proportions of normal and aberrant GMs immediately after the interventions (X^2^(2)=3.56; p = 0.17; V = 0.32), during the WM phase (2–4 weeks post-term; X^2^(2)=3.03; p = 0.25; V = 0.31), and in the FM phase (12–15 weeks post-term; X^2^(1)=1.00; p = 0.60; Phi = 0.17). Cramped-synchronized GMs were observed only once in each group: during the preterm phase in a newborn from the control group, and during the WM phase in a newborn from the experimental group. Abnormal FMs were not observed (Table 2).

**Table 2:** Trajectory of the quality of GMs in the sample. Proportions are comparing groups in each GMA category at each age.

Age	Preterm/Writhing Movements (n = 34) f (%)						Fidgety Movements (n = 33) f (%)			
	Normal		PR		CS		Normal		Absent/Sporadic	
	EG	CG	EG	CG	EG	CG	EG	CG	EG	CG
36-37 wk	10 (66.7)	5 (33.3)	7 (38.9)	11 (61.1)	0 (0)	1 (100)	–	–	–	–
42-44 wk	8 (50.0)	8 (50.0)	5 (35.7)	9 (64.3)	0 (0)	2 (100)	–	–	–	–
52-55 wk	–	–	–	–	–	–	15 (51.7)	14 (48.3)	1 (25.0)	3 (75.0)

EG, experimental group; CG, control group; PR, poor repertoire; CS, cramped-synchronized; f, frequency; wk, postmenstrual weeks.

### Secondary outcomes

There were no differences between the groups for body weight before (t(32)=0.34; p = 0.73; d = 0.12) or after the interventions (t(32)=1.57; p = 0.13; d = 0.52). The experimental group gained 202.35 ± 176.50 grams, while controls gained 288.53 ± 142.26 grams throughout the 10 days of intervention (Table 3).

**Table 3:** Clinical outcomes (n = 34).

Outcomes	EG	CG
**Weight** (M ± SD)		
Before intervention	1705.29 ± 276.97	1736.76 ± 255.77
After intervention	1907.75 ± 261.16	2025.29 ± 181.09
**Hospital Stay** (M ± SD)		
Total	44.82 ± 16.22	49.06 ± 17.29
After intervention	8.00 ± 7.61	11.47 ± 8.86
**Breastfeeding at Discharge** f (%)		
Yes	13 (48.1)	14 (51.9)
No	4 (57.1)	3 (42.9)
**Breastfeeding Maintenance 42–44 wk** f (%)		
Yes	12 (50.0)	12 (50.0)
No	5 (50.0)	5 (50.0)
**Breastfeeding Maintenance 52–55 wk** f (%)		
Yes	9 (52.9)	8 (47.1)
No	4 (36.4)	7 (63.6)
NC	4 (66.7)	2 (33.3)
**SAB Respiratory Distress**		
Before intervention	2 (1-3)	2 (2-3)
After intervention	0 (0-1)	0 (0-2)

EG, experimental group; CG, control group; M, mean; SD, standard deviation; f, frequency; NC, not collected; SAB: Silverman-Andersen Bulletin.

Regarding posture and muscle tone, except for ankle dorsiflexion, groups were comparable before the interventions. After the intervention, the scores for popliteal angle and leg recoil were higher in the experimental group than in the control group, suggesting greater physiological muscle tone. No other differences between groups for posture and muscle tone were found (Table 4).

**Table 4:** Mean and standard deviation values of posture and tonus scores before and after intervention (n = 34).

Outcomes	EG	CG	*p-*value	Cohen’s d
**Posture**				
Before	2.06 ± 0.43	1.94 ± 0.43	0.43	0.28
After	3.06 ± 0.24	2.88 ± 0.33	0.09	0.62
**Arm Recoil**				
Before	2.06 ± 0.56	1.76 ± 0.83	0.23	0.43
After	3.12 ± 0.48	2.88 ± 0.33	0.11	0.58
**Leg Recoil**				
Before	1.94 ± 0.43	1.88 ± 0.33	0.66	0.16
After	2.94 ± 0.75	2.47 ± 0.51	0.04^*^	0.73
**Scarf Sign**				
Before	1.47 ± 0.72	1.24 ± 0.56	0.29	0.36
After	2.35 ± 0.49	2.35 ± 0.49	1.00	0.00
**Popliteal Angle**				
Before	1.82 ± 0.39	1.65 ± 0.61	0.32	0.33
After	2.65 ± 0.49	2.24 ± 0.44	0.01^*^	0.88
**Heel to Ear**				
Before	2.12 ± 0.33	1.88 ± 0.48	0.11	0.58
After	3.00 ± 0.35	2.53 ± 0.51	0.09	1.08
**Ankle Dorsiflexion**				
Before	1.82 ± 0.39	1.41 ± 0.51	0.01^*^	0.90
After	2.52 ± 0.51	2.35 ± 0.49	0.31	0.34
**Head Lag**				
Before	0.76 ± 0.75	1.00 ± 0.71	0.35	0.33
After	2.00 ± 0.50	1.94 ± 0.75	0.79	0.09
**Ventral Suspension**				
Before	0.94 ± 0.75	0.59 ± 0.62	0.14	0.51
After	2.12 ± 0.60	1.71 ± 0.69	0.07	0.64
**Head Elevation**				
Before	1.24 ± 0.44	0.94 ± 0.90	0.23	0.42
After	2.00 ± 0.79	1.71 ± 0.77	0.28	0.37
**Total**				
Before	15.76 ± 2.22	14.06 ± 3.49	0.10	0.58
After	25.94 ± 3.07	23.41 ± 2.87	0.02^*^	0.85

EG, experimental group; CG, control group; *significance at α = 5%; d, Cohen’s d.

For behavioral state, both groups showed a predominance of sleep state immediately before, immediately after, and at the 30-minute follow-up assessment on each intervention day. Specifically, at 30 minutes after the end of the intervention, a higher alert state was observed in the experimental group compared to controls, but only on days 5 (p = 0.001), 7 (p = 0.02), and 8 (p < 0.001) (S1 Table).

No differences were observed between the groups for the length of hospital stay, either for the total length of hospitalization (t(32)=0.74; p = 0.47; d = 0.25) or for the period after the end of the intervention (t(32)=1.22; p = 0.23; d = 0.42) (Table 3).

Before starting the interventions, all newborns were being fed via nasogastric tube. No differences were found between the groups in the establishment of breastfeeding at hospital discharge (X^2^(1)=0.18; p = 1.00; Phi = 0.73), nor in the maintenance of breastfeeding at 42–44 (X^2^(1)=0.18; p = 1.00; Phi = 0.00) and 52–55 (X^2^(1)=1.54; p = 0.46; Phi = 0.21) weeks post-term (Table 3).

### Monitoring outcomes

Concerning vital signs, specifically heart rate, respiratory rate, peripheral oxygen saturation, and body temperature, there were sporadic differences between the groups, which were clinically insignificant. The newborns remained clinically stable throughout the interventions (S2 Table). Respiratory distress was not observed throughout the interventions (U’s < 106.5; p’s > 0.11; r’s > 0.27) (Table 2). No adverse events or signs of clinical deterioration were observed.

## Discussion

This study reports pilot evidence on the effects of a 10-session physical therapy program combined with kangaroo skin-to-skin contact compared to kangaroo skin-to-skin contact alone, administered over 15 days, on neuromotor, clinical outcomes, and vital signs of preterm newborns hospitalized at approximately 34–35 weeks postmenstrual age. Overall, all newborns showed weight gain during the protocol and maintained their vital signs within normal and stable parameters. Additionally, no signs of respiratory and behavioral distress or evident adverse effects were observed. Therefore, based on these outcomes, the intervention was clinically safe.

On the other hand, although the proposed handling techniques were intended to facilitate the newborns’ spontaneous movements, no treatment effects on the quality of GMs were found. This is in line with the results by Fjørtoft et al. [23], in which a parent-administered motor intervention between 34–37 postmenstrual weeks was insufficient to change the quality of FMs compared to standard care. In the study by Ma et al. [22], preterm newborns who underwent early multisensory intervention during hospitalization and after discharge showed no improvements in GMs in the WM age, but improvements in the FMs, compared to routine care. On the other hand, Khurana et al. [24] found a decreased proportion of newborns with poor repertoire GMs more in the neonatal physical therapy group, who received approximately 17 sessions of multisensory intervention for 90 minutes/day, 6 days/week until discharge, than in the routine care group. This suggests that a longer duration of intervention, potentially extended beyond the immediate post-discharge period, may be necessary to achieve more substantial effects on GMs. In our study, the newborns received the intervention across 10 sessions before discharge. Therefore, it is possible that a higher dose, either more frequent sessions prior to discharge [24], or longer duration, such as the continuation of the intervention post-discharge [22], might have produced different outcomes in GMs in this study. Previous research indicates that interventions initiated in the neonatal unit and extended into the home environment, particularly those involving active parental engagement, are associated with the most substantial improvements in early motor and cognitive outcomes before 12 months of age [1].

Besides a limited duration of the intervention, other possible explanations for the lack of intervention effects on the GMs could include mechanisms of neuroplasticity in preterm newborns and the intrinsic variability in GMs. To produce motor variability, supraspinal projections activate, inhibit, and modulate CPG activity, as well as the sensory feedback from the spontaneous movement itself [21,33–35]. In brain injury, the modulation of CPG by these projections results in less variable and abnormal movement [33]. However, almost 90% of our total sample progressed to the presence of FMs, which suggests a normal outcome and a low risk of brain injury. Given the clinical stability of the newborns, with no presence of important neurological risk factors such as grade III or IV peri-intraventricular hemorrhages, we suggest that the intrinsic neuroplastic capacity and the endogenous developmental processes of the brain, even in an immature nervous system in the challenging extra-uterine environment, were sufficient to allow for favorable GM development, regardless of the intervention. Moreover, even under stable conditions, GMs’ variability can fluctuate within and across days, which reflects normal neurodevelopmental processes and ongoing neural reorganization [19,21,33]. This natural intrinsic variability might have masked potential subtle intervention effects, particularly considering the short duration of our intervention. This reinforces that future studies should extend our proposed intervention for the home setting, after discharge.

On the other hand, we found treatment effects for two patterns of muscle tone (leg recoil and popliteal angle). This suggests that the sensory-motor intervention associated with skin-to-skin contact was better at improving the physiological flexor tone of the lower limbs than skin-to-skin contact alone. Previous studies [15,16,24,36–39] have demonstrated that sensory-motor interventions have beneficial effects on neuromuscular development in preterm newborns when assessed using tools based on Dubowitz et al. [29]. According to Amiel-Tison [40], the preterm flexor tone in the limbs observed in the popliteal angle begins 4–6 weeks earlier in the legs than in the arms, and leg recoil is detected 2–3 weeks earlier than arm recoil. This might explain why changes were not observed in the upper limb patterns. We suggest that the intervention benefited the caudocephalad tonus maturation expected in typical development. The newborns likely benefited from the body handling, midline postures, and enriched opportunities to perform spontaneous body movements, which might have facilitated the physiological tonus development.

The fact that all newborns in the study received kangaroo skin-to-skin contact may have contributed to equalizing the groups in terms of weight gain, hospitalization duration, breastfeeding outcomes, and, perhaps, even GMs. There is wide evidence that preterm newborns who undergo skin-to-skin contact tend to have shorter hospital stays [9,41] and better breastfeeding both at hospital discharge and post-discharge [10,42,43]. Skin-to-skin contact has also been associated with better weight gain in low birthweight preterm newborns [41]. Recent research has shed light on the neurobiological mechanisms through which skin-to-skin contact benefits preterm newborns, particularly highlighting the role of C-tactile fibers – mechanoreceptors activated by gentle, affective touch. They are hypothesized to play a central role in modulating stress responses and promoting caregiver-newborn bonding, which supports their physiological stability. This may further benefit clinical outcomes and early neurodevelopment [4–6]. The present study adds to the body of evidence supporting skin-to-skin contact as a core element of kangaroo mother care, even within standard neonatal practices. This can motivate future research to clarify the relationship between affective touch during skin-to-skin contact and early clinical and neuromotor outcomes.

We acknowledge the limitations of this study. First, the results may not be generalizable to other populations of preterm newborns with different clinical characteristics or other hospital settings. Moreover, it was not possible to control for interventions such as diet or medication use. We also acknowledge that additional potential confounding factors, such as maternal involvement, breastfeeding practices, and maternal stress level, were not controlled for, despite their possible influence on neurodevelopmental outcomes. Another limitation was the lack of blinding of the examiner to group allocation when assessing posture and muscle tone, and behavioral state, which increased the risk of bias in the evaluation of these variables. Therefore, the findings related to these outcomes should be interpreted with caution. Caution is also advised when interpreting the p-values associated with multiple testing, as observed for posture and muscle tone. Additionally, although a sample size calculation was adhered to, the number of participants can be considered small. In light of these limitations, alongside the clinical relevance and the potential yet non-confirmatory nature of the findings, this research should be regarded as a pilot study. Future trials should include a larger cohort to enhance both the internal and external validity of the findings. Despite these limitations, by mimicking an intervention adopted in the clinical practice of physical therapists, this study provides new insights for evidence-based neonatal intervention practice.

### Clinical implications

Ten sessions of 15-minute sensory-motor intervention associated with 60-minute skin-to-skin contact over 15 days can be recommended for newborns at 34–35 weeks postmenstrual age and with an average initial weight of 1700 grams if the goal is to favor the muscle tone in the lower limbs. However, we do not recommend the intervention if the sole therapeutic objectives are to improve the newborns’ quality of GMs, body weight gain, and breastfeeding, or reduce the length of hospitalization, as these outcomes can also be achieved by receiving skin-to-skin contact alone within routine hospital care.

## Conclusion

The sensory-motor intervention combined with kangaroo skin-to-skin contact was a clinically safe technique for stable preterm newborns, as no adverse effects were observed, and vital signs and clinical outcomes remained favorable. The intervention provided no advantages for the quality of GMs in the writing or fidgety movements phases; however, the maturation of lower limb tone might have been positively affected. A confirmatory larger trial is needed to further examine these neuromotor findings.

## Supporting information

S1 Table Proportions of newborns in the categories of behavioral state, adjusted mean differences between groups (M, estimates) and 95% confidence intervals (CI) according to the day of intervention and the time of assessment (n = 34). Categories were merged into: (A) sleep – inactive sleep, active sleep, drowsiness; (B) alert (inactive alert, active alert), and (C) crying.
(DOCX)

S2 Table Mean values and standard deviation of heart rate (HR), respiratory rate (RR), peripheral oxygen saturation (SpO2), and body temperature (BT), according to the day of intervention and the time of assessment (n = 34). *group differences (p < 0,05).
(DOCX)

S1 Checklist CONSORT Checklist. (PDF)

S1 File Project_English. (PDF)

S2 File Project_OriginalPt. (PDF)
