# Supplementary material for: Effect of sensory-motor intervention associated with skin-to-skin contact on neuromotor and clinical outcomes of preterm newborns: A randomized controlled trial
Source: PLoS One. 2025 Sep 12;20(9):e0332269. doi: 10.1371/journal.pone.0332269 (PMC12431241; doi:10.1371/journal.pone.0332269)
Supplement: S1 Table — Categories were merged into: (A) sleep – inactive sleep, active sleep, drowsiness; (B) alert (inactive alert, active alert), and (C) crying. (DOCX) [file pone.0332269.s001.docx]

**S1 Table. Proportions of newborns in the categories of behavioral state, adjusted mean differences between groups (M, estimates) and 95% confidence intervals (CI) according to the day of intervention and the time of assessment (n=34). Categories were merged into: (A) sleep - inactive sleep, active sleep, drowsiness; (B) alert (inactive alert, active alert), and (C) crying.**

| **Day** | **Groups** | **Before** | **M (95% CI)** | **After** | **M (95% CI)** | **30 min** | **M (95% CI)** |
| --- | --- | --- | --- | --- | --- | --- | --- |
| Day 1 | EG | A (72.2%)  B (27.8%)  C (0) | 0 | A (72.2%)  B (22.2%)  C (5.6%) | 0 | A (100%)  B (0)  C (0) | 0 |
|  | CG | A (75%)  B (25%)  C (0) |  | A (87.5%)  B (12.5%)  C (0) |  | A (62.5%)  B (37.5%)  C (0) |  |
| Day 2 | EG | A (72.2%)  B (22.2%)  C (5.6%) | 0.11 (-0.16-0.38) | A (83.3%)  B (16.7%)  C (0) | -0.17  (-0.43-0.10) | A (72.2%)  B (27.8%)  C (0) | 0.28 (0.00-0.55) |
|  | CG | A (100%)  B (0)  C (0) |  | A (87.5%)  B (12.5%)  C (0) |  | A (81.2%)  B (18.8%)  C (0) |  |
| Day 3 | EG | A (94.4%)  B (5.6%)  C (0) | -0.22  (-0.49-0.05) | A (66.7%)  B (33.3%)  C (0) | 0.00 (-0.26-0.26) | A (77.8%)  B (22.2%)  C (0) | 0.22 (-0.05-0.49) |
|  | CG | A (87.5%)  B (12.5%)  C (0) |  | A (81.3%)  B (18.7%)  C (0) |  | A (68.8%)  B (31.2%)  C (0) |  |
| Day 4 | EG | A (72.2%)  B (27.8%)  C (0) | 0.00 (-0.27-0.27) | A (72.2%)  B (27.8%)  C (0) | -0.06  (-0.32-0.21) | A (77.8%)  B (22.2%)  C (0) | 0.22 (-0.05-0.49) |
|  | CG | A (87.5%)  B (12.5%)  C (0) |  | A (93.8%)  B (6.2%)  C (0) |  | A (81.3%)  B (18.7%)  C (0) |  |
| Day 5 | EG | A (72.2%)  B (27.8%)  C (0) | 0.00 (-0.27-0.27) | A (77.8%)  B (16.7%)  C (5.5%) | -0.06  (-0.32-0.21) | A (55.6%)  B (44.4%)  C (0) | 0.44 (0.17-0.72)* |
|  | CG | A (87.5%)  B (12.5%)  C (0) |  | A (81.3%)  B (18.7%)  C (0) |  | A (75%)  B (25%)  C (0) |  |
| Day 6 | EG | A (100%)  B (0)  C (0) | -0.28  (-0.55-0.01) | A (83.3%)  B (16.7%)  C (0) | -0.17  (-0.43-0.10) | A (88.9%)  B (11.1%)  C (0) | 0.11 (-0.16-0.38) |
|  | CG | A (81.3%)  B (6.3%)  C (12.4%) |  | A (100%)  B (0)  C (0) |  | A (81.3%)  B (18.7%)  C (0) |  |
| Day 7 | EG | A (88.9%)  B (11.1%)  C (0) | -0.17  (-0.43-0.10) | A (72.2%)  B (27.8%)  C (0) | -0.06  (-0.32-0.21) | A (66.7%)  B (33.3%)  C (0) | 0.33 (0.06-0.61)* |
|  | CG | A (75%)  B (25%)  C (0) |  | A (81.3%)  B (18.7%)  C (0) |  | A (68.8%)  B (31.2%)  C (0) |  |
| Day 8 | EG | A (100%)  B (0)  C (0) | -0.28  (-0.55-0.01) | A (77.8%)  B (22.2%)  C (0) | -0.11  (-0.37-0.15) | A (55.6%)  B (33.3%)  C (11.1%) | 0.56 (0.28-0.83)* |
|  | CG | A (93.8%)  B (6.2%)  C (0) |  | A (68.8%)  B (31.2%)  C (0) |  | A (75%)  B (25%)  C (0) |  |
| Day 9 | EG | A (88.8%)  B (5.6%)  C (5.6%) | -0.11  (-0.38-0.16) | A (72.2%)  B (27.8%)  C (0) | -0.06  (-0.32-0.21) | A (94.4%)  B (5.6%)  C (0) | 0.05 (-0.22-0.33) |
|  | CG | A (87.4%)  B (6.3%)  C (6.3%) |  | A (75%)  B (25%)  C (0) |  | A (81.3%)  B (18.7%)  C (0) |  |
| Day 10 | EG | A (100%)  B (0)  C (0) | -0.28  (-0.55-0.01) | A (72.2%)  B (27.8%)  C (0) | -0.06  (-0.32-0.21) | A (83.3%)  B (16.7%)  C (0) | 0.17 (-0.10-0.44) |
|  | CG | A (81.3%)  B (18.7%)  C (0) |  | A (93.8%)  B (6.2%)  C (0) |  | A (81.3%)  B (18.7%)  C (0) |  |

* p-value < 0.05.
