# Supplementary material for: Effect of sensory-motor intervention associated with skin-to-skin contact on neuromotor and clinical outcomes of preterm newborns: A randomized controlled trial
Source: PLoS One. 2025 Sep 12;20(9):e0332269. doi: 10.1371/journal.pone.0332269 (PMC12431241; doi:10.1371/journal.pone.0332269)
Supplement: S2 Table — *group differences (p < 0,05). (DOCX) [file pone.0332269.s002.docx]

**S2 Table**. **Mean values and standard deviation of heart rate (HR), respiratory rate (RR), peripheral oxygen saturation (SpO2), and body temperature (BT), according to the day of intervention and the time of assessment (n=34).**

| **Before After 30 min** | | | |
| --- | --- | --- | --- |
| **SpO2 Mean (desvio padrão) Média (desvio padrão) Média (desvio padrão)** | | | |
| Day 1 | 97.18 (2.31) | 97.82 (1.48) | 97.47 (1.59) |
| Day 2 | 97.35 (2.00) | 98.15 (1.37) | 97.62 (2.18) |
| Day 3 | 97.32 (2.40) | 98.12 (1.49) | 98.00 (1.68) |
| Day 4 | 97.39 (1.81) | 97.96 (1.43) | 97.48 (1.54) |
| Day 5 | 97.42 (2.21) | 97.58 (1.80) | 97.63 (2.01) |
| Day 6 | 96.84 (2.09) | 97.37 (1.98) | 97.05 (1.75) |
| Day 7 | 97.27 (2.25) | 97.70 (1.60) | 97.85 (1.52) |
| Day 8 | 97.65 (1.67) | 97.91 (1.41) | 97.70 (1.66) |
| Day 9 | 97.83 (1.59) | 97.88 (1.34) | 98.93 (1.93) |
| Day 10 | 97.49 (1.86) | 97.74 (1.56) | 97.71 (1.40) |
| **HR** | | | |
| Day 1 | 146.32 (15.85) | 146.12 (12.06) | 146.56 (15.21) |
| Day 2 | 152.74 (16.61) | 149.53 (12.87) | 148.97 (12.56) |
| Day 3 | 149.76 (13.46) | 148.09 (11.89) | 150.88 (11.87) |
| Day 4 | 152.13 (12.36) | 147.47 (14.23) | 147.43 (12.32) |
| Day 5 | 153.05 (14.34) | 143.79 (14.13) | 149.27 (14.28)^*^ |
| Day 6 | 148.38 (14.77)^*^ | 146.74 (12.97)^*^ | 147.48 (13.57)^*^ |
| Day 7 | 147.51 (17.07)^*^ | 142.56 (13.12) | 144.45 (14.86)^*^ |
| Day 8 | 149.15 (12.46)^*^ | 148.29 (11.75) | 152.49 (8.56) |
| Day 9 | 151.46 (14.59) | 148.17 (9.14) | 148.39 (9.28) |
| Day 10 | 147.95 (11.71) | 146.86 (8.52) | 150.14 (9.29) |
| **RR** | | | |
| Day 1 | 49.74 (6.17) | 42.85 (5.40) | 45.85 (5.78) |
| Day 2 | 48.76 (7.90) | 42.47 (5.88) | 45.00 (4.28) |
| Day 3 | 48.18 (7.37) | 42.88 (5.49) | 46.26 (5.53) |
| Day 4 | 48.11 (5.56) | 41.20 (3.86) | 43.64 (4.63) |
| Day 5 | 47.30 (6.14) | 41.84 (4.94) | 44.62 (4.92) |
| Day 6 | 47.44(6.37) | 42.07 (3.75) | 45.18 (5.12) |
| Day 7 | 46.14 (5.92) | 43.29 (4.93) | 44.72 (4.70) |
| Day 8 | 46.61 (6.11) | 42.78 (5.72) | 45.51 (5.69) |
| Day 9 | 46.17 (4.61) | 40.56 (4.46) | 43.46 (4.54) |
| Day 10 | 45.75 (3.51) | 41.26 (4.97) | 43.97 (3.43) |
| **BT** | | | |
| Day 1 | 36.58 (0.31) | 36.65 (0.20) |  |
| Day 2 | 36.72 (0.35)^*^ | 36.75 (0.26) |  |
| Day 3 | 36.72 (0.28)^*^ | 36.78 (0.24) |  |
| Day 4 | 36.72 (0.24)^*^ | 36.84 (0.40) |  |
| Day 5 | 36.70 (0.32) | 36.74 (0.14) |  |
| Day 6 | 36.64 (0.23) | 36.71 (0.16) |  |
| Day 7 | 36.67 (0.24) | 36.76 (0.19) |  |
| Day 8 | 36.64 (0.27) | 36.72 (0.19) |  |
| Day 9 | 36.67 (0.24) | 36.74 (0.17) |  |
| Day 10 | 36.63 (0.23) | 36.70 (0.12) |  |
| *group differences (p<0,05). | | | |
