# Supplementary material for: Effect of sensory-motor intervention associated with skin-to-skin contact on neuromotor and clinical outcomes of preterm newborns: A randomized controlled trial
Source: PLoS One. 2025 Sep 12;20(9):e0332269. doi: 10.1371/journal.pone.0332269 (PMC12431241; doi:10.1371/journal.pone.0332269)
Supplement: S1 File — (PDF) [file pone.0332269.s004.pdf]

Universidade Federal de Mato Grosso do Sul
Programa de Pós-Graduação em Saúde e Desenvolvimento da Região Centro-Oeste

Mariane de Oliveira Nunes Reco

Project Submitted to the Ethics Committee

Longitudinal Repercussions of a Physiotherapeutic Intervention Protocol Associated with Kangaroo Positioning in Preterm Newborns: A Randomized Controlled Clinical Trial

Campo Grande
2018

Mariane de Oliveira Nunes Reco

Project Submitted to the Ethics Committee

Longitudinal Repercussions of a Physiotherapeutic Intervention Protocol Associated with Kangaroo Positioning in Preterm Newborns: A Randomized Controlled Clinical Trial

Project submitted to the Ethics Committee and presented to the Graduate Program in Health and Development in the Central-West Region at the Federal University of Mato Grosso do Sul as a requirement for the Ph.D. selection process.
Advisor: Prof. Dr. Daniele de Almeida Soares Marangoni

Campo Grande
2018

Abstract

Preterm newborns are vulnerable to perinatal conditions due to their organic immaturity and associated external factors. This association can lead to neurological impairments, affecting motor development, respiratory mechanics, and the establishment of breastfeeding. Considering the current approach to the humanization of neonatal care, as well as the recent Brazilian epidemiological need for studies focused on early intervention in hospitalized newborns at risk or with established neurological problems, the necessity for therapeutic investigations that help prevent and minimize morbidities in this population becomes evident. The primary objective of this study is to evaluate the short- and medium-term effects of a physiotherapeutic intervention protocol associated with kangaroo positioning on the neuromotor behavior and clinical outcomes of preterm newborns in a neonatal unit. A randomized controlled trial with a parallel group design and balanced distribution ratio will be conducted. A total of 34 preterm newborns, with more than 72 hours of postnatal life and stable clinical conditions, admitted to Neonatal Intermediate Care Units will participate in this study. They will be randomly divided into two groups: a) Experimental Group (EG), which will receive the physiotherapeutic intervention protocol associated with kangaroo positioning; and b) Control Group (CG), which will receive only the kangaroo position. The protocol will be implemented over 10 consecutive days, with each session of the physiotherapeutic intervention lasting 15 minutes and 60 minutes of kangaroo positioning, during the period from 34 to 36 weeks and 6 days of postmenstrual age. Primary outcomes will include weight gain at the end of the protocol; Prechtl's general movements assessment at 36 weeks postmenstrual and 12 weeks post-term. Secondary outcomes will include posture and muscle tone, behavioral state, time to establish and maintain breastfeeding, duration of hospitalization, and vital signs. This study will provide experimental support for evidence-based practice, and its results may be used to guide feasible intervention protocols for the routines of neonatal unit teams in Brazilian hospitals.

Keywords: Early stimulation. Kangaroo method. Preterm newborn. Child development.

1 Introduction

Preterm birth occurs when delivery happens before completing 37 weeks of gestation or less than 259 days, counted from the first day of the last menstrual period (WORLD HEALTH ORGANIZATION, 2010), resulting in the birth of babies with biological immaturity for extrauterine life.

The frequency of preterm birth has been increasing worldwide, including in Brazil. The estimated global prevalence of preterm birth in the country for the period 2011-2012 was 11.5% (LEAL et al., 2016). According to the World Health Organization (BLENCOWE, 2013), Brazil is among the ten countries that most contribute to the rising number of preterm newborns globally. The Central-West region ranks fifth among Brazilian regions, with an incidence of 6.2% of preterm births in the country (LEAL et al., 2016). The state of Mato Grosso do Sul recorded a proportion of 11.8% of preterm births in 2015 (SINASC, 2017). These data draw the attention of public policies since perinatal conditions responsible for infant mortality in Brazil are particularly common in this newborn population (BRASIL, 2011a).

Gestational age and birth weight are some of the biological variables involved in newborn delivery. Preterm newborns with low birth weight (< 2500 g) are especially vulnerable to perinatal conditions due to several organic and external factors. Organic immaturity, particularly of the pulmonary and neurological systems, compromises gas exchange, increasing the risk of death and morbidities related to neurological impairments (BRASIL, 2011a). The inefficient production of surfactant (LEMOS; MAUX; PAIVA; 2013), associated with structural immaturity, imposes significant limitations on early extrauterine breathing (FRIEDRICH; CORSO; JONES, 2005). These newborns may experience variability in gas exchange, leading to periods of hypoxia and hyperoxia, hypo- or hypercapnia, altering the brain blood flow autoregulatory capacity and increasing the risk of brain injuries (LIEM; GREISEN, 2010). This condition can compromise brain oxygenation and, consequently, the organization and functioning of the central nervous system during a period of significant development (ALBERTINE, 2012; HAGBERG; JACOBSSON, 2005).

Neuromotor system immaturity is another intrinsic barrier to the normal development of preterm newborns. Due to this immaturity and environmental factors, preterm newborns may not experience adequate movement. Without appropriate experiences, they are less capable of perceiving stimuli, and with altered perception, they are less able to learn (DE GROOT, 2000) and perform spontaneous movements against gravity (GRENIER et al., 2003; VAIVRE-DOURET et al., 2004). The human fetus exhibits specific movement patterns generated

endogenously, emerging between the 9th and 12th postmenstrual weeks and continuing after birth, regardless of when birth occurs. These early spontaneous motor patterns include the so-called general movements, which are present from early fetal life until the 5th month after term age (EINSPIELER; PRECHTL, 2005; EINSPIELER et al., 1997).

During the preterm period, normal general movements involve the whole body, occur frequently, and last long enough to be observed. They follow a variable sequence of arm, leg, neck, and trunk movements, increasing and decreasing in intensity, strength, and speed, with gradual onset and end. Rotations along the limb axes and small directional changes make these movements fluid and elegant, giving the impression of complexity and variability (EINSPIELER; PRECHTL, 2005). From term age until the 6th or 9th post-term week, general movements take on an elliptical form and are characterized by moderate-speed movements with smaller amplitude, closer to the midline, known as writhing movements. From the preterm period to the 9th post-term week, motor patterns differing from the described general movements are considered abnormal and can be classified as poor repertoire, characterized by monotonous movement sequences; cramped-synchronized, involving rigid movements lacking the normal smooth and fluent character; or chaotic, characterized by large-amplitude movements devoid of fluency and elegance (EINSPIELER; PRECHTL, 2005; EINSPIELER et al., 1997).

Around the 6th to 9th post-term week, a shift occurs in movement patterns of the limbs, trunk, and head, as writhing movements gradually disappear while new movements with lower amplitude, moderate speed, and variable acceleration emerge in all directions. These movements, present continuously while the baby is awake, are called fidgety movements. These movements are typically continuous and most evident from the 9th to the 15th-20th post-term week, gradually disappearing as intentional movements begin to emerge. Abnormal or absent fidgety movements indicate a higher risk of later neurological dysfunctions, whereas normal fidgety movements are predictive of normal development (EINSPIELER; PEHARZ; MARSCHIK, 2016; EINSPIELER; PRECHTL, 2005; EINSPIELER et al., 1997).

Preterm newborns, due to biological and environmental factors, may experience limited spontaneous movement during their stay in neonatal care units (BRACEWELL, MARLOW, 2002) when compared to full-term newborns (FALLANG et al., 2005). These newborns may exhibit monotonous, rigid, or irregular movement sequences due to impaired postural control (FALLANG et al, 2005), losing the characteristics of fluency, complexity, and variability of spontaneous movements, as well as displaying less evident fidgety movements (EINSPIELER; PEHARZ; MARSCHIK, 2016).

Associated with organic factors, external or environmental factors necessary for the clinical care of preterm newborns in intensive care units can cause deficits in motor, sensory, mental, and emotional aspects. External factors such as maternal separation and inadequate sensory stimulation, sometimes with insufficient stimuli or excessive ones, enhance the risks for morbidities and negatively impact proper development.

All references to the initial relationships between the mother and preterm newborn are altered in the face of the separation of the dyad in the neonatal unit. This separation limits the formation of the mother-child bond, hinders the breastfeeding process (AMIN et al., 2000; BRASIL, 2009; SANTORO JUNIOR; MARTINEZ, 2007), and interferes with the organization and development of brain circuits involved in language and social communication (CALDAS, 2016). Thus, the preterm newborn is initially susceptible to the deprivation of the benefits of breastfeeding and to socio-communicative problems in their development. As an aggravating factor, preterm newborns, during the early stages of central nervous system development, are routinely subjected to painful procedures and excessive sensory stimulation in neonatal units (CARBAJAL et al., 2005; SHAREK et al., 2006; SIMONS et al., 2003). Such stimuli can lead to changes in pain processing, behavioral changes, and reduced volume in sensory brain areas (GASPARDO; LINHARES; MARTINEZ, 2005; LAGO et al., 2009; NEWNHAM; INDER; MILGROM, 2009).

Along the developmental trajectory, in addition to risk factors, which expose newborns to multiple and continuous adverse events, there are also protective factors for development. When the response to these events is frequent, intense, or prolonged and is associated with the absence or scarcity of protective factors, the negative impact, particularly on the neurological system, can be extremely toxic. There is a link between exposure to early adversities, leading to toxic stress, and subsequent deficiencies in learning, behavior, and physical and mental well-being (SHONKOFF; GARMER, 2012). Moreover, prolonged hospitalization negatively influences the motor (PANCERI et al., 2012) and socio-communicative (CALDAS, 2017) development of babies hospitalized for more than thirty days, placing the preterm newborn population under considerable vulnerability to problems in social, cognitive, and motor development. The consequences of this exposure may be transient, but they can also result in chronic motor development dysfunctions that extend beyond the neonatal period (FORMIGA; LINHARES, 2009).

In recent decades, the effects of prematurity on motor development have been reported in several studies, which pointed to significant differences in the motor development of preterm and term newborns, with lower scores in motor evaluation outcomes for preterm babies

(FORMIGA et al., 2015; FUENTARIA; SILVEIRA; PROCIANOY, 2017). For example, preterm babies showed a tendency to exhibit disadvantages in manual skills behavior between 3 and 8 months of corrected age (HEATHCOCK; LOBO; GALLOWAY, 2008; SOARES-MARANGONI; VON HOFSTEN; TUDELLA, 2012), less skill in more refined manual tasks (SOARES-MARANGONI; CUNHA; TUDELLA, 2014), lower ability to sit (FORMIGA; CEZAR; LINHARES, 2010), and greater motor delay in sitting and standing postures at 8 months of corrected age (PIN et al., 2009). In school age, children born preterm are more susceptible to developmental impairments in motor, behavioral, and academic performance areas (MOREIRA; MAGALHÃES; ALVES, 2014), typically showing worse motor coordination and a higher incidence of cerebral palsy than those born at term (ODD et al., 2013). These children may exhibit reduced corticomotor excitability in brain areas associated with motor control, even without cerebral injury (PITCHER et al., 2012).

Preterm newborns also have greater vulnerability to socio-communicative problems (CALDAS et al., 2016; GUINCHAT et al., 2012) when compared to term newborns (DE SHUYMER et al., 2011; SHAH et al., 2013). Some studies report that preterm newborns may exhibit atypical behavioral characteristics, such as difficulty in maintaining visual attention before three months, longer processing time in response to stimuli (SHAH et al., 2013), less active exploration of objects, and reduced joint activities with the mother (DAWSON et al., 2012).

These studies demonstrate that the factors associated with prematurity can result in motor development problems in the medium and long term, thus reaffirming the need for evaluation, monitoring, stimulation, and early intervention measures with an emphasis on preventing these problems in preterm newborns (FORMIGA; PEDRAZZANI; TUDELLA, 2004).

To minimize the negative effects of prematurity on child development, changes were made in hospital practices to better adapt the baby to extrauterine life. Since the 1990s, the Ministry of Health has incorporated the Kangaroo Method into health policies as a strategy for humanizing neonatal care and promoting family citizenship, thereby maximizing protective factors for the development of preterm newborns (BRASIL, 2013; 2014). The Kangaroo Method includes humanized care, skin-to-skin contact between the newborn and their parents, environmental control, pain reduction, family care, and support from the health care team (BRASIL, 2016). Despite these efforts, hospital units still expose newborns to discomfort and pain within their routine care (REICHERT; LINS; COLLET, 2007), leading to stress, increased energy expenditure, risk of hypoxia, and breastfeeding difficulties (GRUNAU, 2002).

Therefore, interdisciplinary therapeutic measures are necessary to improve the comfort of preterm newborns (GASPARDO; LINHARES; MARTINEZ, 2005) to favor the success of breastfeeding and the motor and socio-communicative development of this population. One therapeutic measure used within the Kangaroo Method to promote the care of newborns in neonatal units is the Kangaroo Position, which provides a containment experience simulating the intrauterine environment from which the newborn has been deprived. The Kangaroo Position involves keeping the newborn in a vertical position, skin-to-skin, against the parent's chest, with the baby wearing only a diaper. Kangaroo positioning begins early and increases progressively, at the family's free choice, for as long as both parties find it enjoyable and sufficient. However, each time the baby is placed in this position, it is recommended that the baby stays for at least one hour to receive its benefits. It should be performed in a guided, safe manner and with the support of a trained healthcare team (BRASIL, 2013; 2016). The Kangaroo Position positively influences the newborn's cardiorespiratory function. The slightly inclined vertical position during Kangaroo positioning favors respiratory mechanics, increasing diaphragm efficiency and lung function, with improvements in respiratory patterns and a reduction in grunting (LUDINGTON-HOE et al., 1999), peripheral oxygen saturation (BERA et al., 2014; HUNT, 2008; LUDINGTON-HOE; FERREIRA; GOLDSTEIN, 1998; SOUKKA et al., 2014), reduction in respiratory rate (BERA et al., 2014; FOHE; KROPF; AVENARIUS, 2000), and maintenance of heart rate within normal ranges (BEGUM et al., 2008; BERA et al., 2014).

Most studies have pointed out the benefits of the Kangaroo Position on the motor development and behavior of preterm newborns in the short, medium (BARRADAS et al., 2006; DAVID et al., 2012; DINIZ et al., 2013; MOTA; SÁ; FROTA, 2005; PADILHA; STEIDL; BRAZ, 2014), and long term (FELDMAN; ROSENTHAL; EIDELMAN, 2014; SCHNEIDER et al., 2012), generalized movements of the newborn (REINAUX, 2005), establishment of exclusive breastfeeding (JAYARAMAN et al., 2017; SANTOS; AZEVEDO FILHO, 2016; PADILHA; STEIDL; BRAZ, 2014), improvement in mother-infant interaction (NUNES et al., 2017), better weight gain and growth (EVEREKLIAN; POSMONTIER, 2017; SHARMA; MURKI; PRATAP, 2016), and reduction in stress and pain (MAIA; AZEVEDO; GONTIJO, 2011; RUSH, 2016; SANTOS; AZEVEDO FILHO, 2016). Preterm newborns subjected to Kangaroo Position show an increase in physiological flexor tone (DINIZ et al., 2013), assuming a posture of greater limb flexion, associated with greater trunk flexion when assessed using the Dubowitz method (BARRADAS et al., 2006; GHISI; IWABE; TORELLO, 2004; MOTA; SÁ; FROTA, 2005). Some authors also suggest that Kangaroo positioning may

have a positive effect on motor development in preterm newborns when assessed using the Alberta Infant Motor Scale (DAVID et al., 2012).

On the other hand, other studies investigating the effects of the Kangaroo Position on generalized movements of preterm newborns found no significant differences in these movements before and after positioning (CONSTANTINOU et al., 1999). Another study, using the Test of Infant Motor Performance (TIMP), observed that babies subjected to the same positioning initially presented motor performance above expectations but showed delays in hip and lower limb movements during antigravity movements and standing position at 44-48 weeks of corrected age (REINAUX, 2005), suggesting that another intervention could be considered in conjunction with the Kangaroo Position. Shaikh and Namrata (2017) compared two groups of preterm newborns: one group received therapeutic massage associated with Kangaroo Position, and the other received only the Kangaroo Position. The authors concluded that the group combining therapeutic massage with the Kangaroo Position showed more significant improvement in physiological and behavioral states compared to the group that received only the Kangaroo Position.

Due to its numerous benefits and being a safe, low-cost intervention, the Kangaroo Mother Method can be implemented in any hospital and provides the multiprofessional team with an important additional resource in the care and attention of preterm infants and their families (BOUNDY et al., 2016; PADILHA; STEIDL; BRAZ, 2014). When designing interventions for Kangaroo Mother care, contextual factors and sociocultural norms need to be taken into consideration (CHAN et al., 2016). The physiotherapist in the neonatal unit, as part of the multiprofessional team, should focus on the physiological, respiratory, and motor needs of the preterm newborn, establishing an appropriate relationship and interaction between the baby and the family, as well as stimulating and clarifying the importance of the Kangaroo Position (AZEVEDO; CALIXTO; ABREU, 2017; BRADY; SMITH, 2015; SWEENEY et al., 2010).

Another important strategy to improve the development of preterm newborns are early intervention programs, initiated still in the hospital environment, with the aim of enhancing the child's social, cognitive, and motor development. Early intervention can be seen as a set of neurodevelopmental evaluation actions and therapies practiced through motor and sensory stimulation with the goal of ensuring adequate development for preterm newborns, promoting an efficient transition from the hospital environment to the community and home (GARCIA; GEPHART, 2013; OBERG et al., 2012). The preterm newborn is unable to maintain postural organization due to their muscle hypotonia and difficulty in self-organization. This, combined

with the positioning in the wide environment of the incubator/cradle and the action of gravity, facilitates the fixation of the newborn in an extended posture, favoring the development of muscle retractions, which may lead to delays in motor development (CARVALHO; SIQUEIRA, 2013) and spontaneous movement (CABRAL; SCHETTINO; POMPEU, 2015; EINSPIELER; PEHARZ; MARSCHIK, 2016). Early intervention handling aims to facilitate the maturational adjustment of the flexor chain (FERREIRA; SANTOS, 2016), in order to stimulate the body organization to favor spontaneous global movement (RAMACHANDRAN; DUTTA, 2013). Intervention in the first few months of life helps in the modulation of muscle tone, improving posture, enhancing motor skills (MARLOW, 2004), and consequently providing appropriate motor experiences.

Early intervention programs for preterm newborns have a positive influence on cognitive and motor outcomes during childhood, with persistent cognitive benefits during preschool age (SPITTLE, 2015; VANDERVEEN et al., 2009). A systematic review with meta-analysis compiled evidence on motor intervention for preterm newborns and suggested that interventions that extend beyond the neonatal care period may have an impact on the motor development of preterm infants, with the strongest effects observed before 6 months of age, particularly when interventions were specifically directed at motor skills (HUGHES; REDSELL; GLAZEBROOK, 2016). Another review linked the components of early intervention (education and psychosocial support for parents and support for child development) to maternal psychosocial aspects and observed that interventions including psychosocial support resulted in better outcomes for mothers of preterm infants (BENZIES, 2013). The integration of the family is necessary for the success of early intervention programs (FORMIGA; PEDRAZZANI; TUDELLA, 2010; OBERG et al., 2012; SWEENEY, 2010). In general, early intervention aims to promote the baby’s interaction with the environment through sensorimotor stimuli, leading to responses closer to the normal pattern and inhibiting the learning of inappropriate postures and movements (GONÇALVES, 2012). These stimuli must be carefully designed based on a thorough evaluation of the preterm newborn and follow their neuroplastic phases, meaning the amount of stimuli should be closely related to the capacity, interest, and possibilities of each newborn (FORMIGA; PEDRAZZANI; TUDELLA, 2010).

Among the standardized instruments for identifying atypical motor patterns, we highlight Prechtl's method for assessing General Movements (GMA). It allows a qualitative assessment of movements, is non-invasive and non-intrusive, and is used as a diagnostic tool for early detection of brain dysfunction, considered an advance in predicting

neurodevelopmental impairment (EINSPIELER; PRECHTL, 2005; EINSPIELER; PEHARZ; MARSCHIK, 2016; NOVAK et al., 2013). For newborns discharged from neonatal intensive care units, it is important to use reliable scales and methods with proven sensitivity and specificity to properly direct this population to early intervention programs.

Based on the context outlined and considering that there is insufficient evidence using a physiotherapeutic intervention protocol associated with Kangaroo Position in neonatal units, some questions arise: a) Is the combination of a physiotherapeutic protocol with Kangaroo Position more effective in improving clinical outcomes, including body weight, breastfeeding, behavioral state, length of hospital stay? b) Is the combination of a physiotherapeutic protocol with Kangaroo Position more effective in improving posture and tone, and normalizing general movements, in newborns with poor motor repertoire than Kangaroo Position alone?

Considering the evidence that early intervention (SYMINGTON; PINELLI, 2016) and Kangaroo Position (NUNES et al., 2017) improve self-regulation and the mother-infant bond, it is hypothesized that the application of the physiotherapeutic intervention protocol associated with Kangaroo Position over fifteen days in stable preterm newborns hospitalized in a neonatal unit will lead to an improvement in clinical outcomes while safely maintaining vital signs. As a consequence, and based on the beneficial effects of Kangaroo Position (SANTOS; FILHO, 2016) and early intervention (MARLOW, 2004) on body posture, muscle tone, and spontaneous movement, it is expected that these newborns will show better motor patterns immediately after the protocol, and at 12 weeks post-term, compared to control newborns.

2 Justification

This study will investigate the various effects (physiological, neurobehavioral, and motor) of a physiotherapeutic intervention protocol associated with Kangaroo Positioning in a neonatal unit at the University Hospital of the Federal University of Mato Grosso do Sul. The specific aim is to investigate the effect of this protocol on the establishment and maintenance of breastfeeding, respiratory discomfort, muscle tone, body posture, and, particularly, the impact on motor development during the hospital period and the critical months after hospital discharge.

Given the absence of scientific evidence correlating a physiotherapeutic intervention protocol with Kangaroo Positioning using a randomized controlled clinical trial method, this project will provide experimental foundations for evidence-based practice through a feasible protocol for neonatal unit teams’ routines. The results of this project may provide scientific

support for implementing the physiotherapeutic intervention protocol associated with Kangaroo Positioning as a procedure to support the protection of child development and the humanized care of preterm newborns.

This project aims to establish an early intervention protocol at the hospital level, and consequently, it will contribute to the Brazilian epidemiological need for more studies focused on early stimulation (BRAZIL, 2016) in hospitalized newborns at risk or with established neurological problems.

3 Objective

3.1 General objective

To investigate the short- and medium-term effects of a physiotherapy intervention protocol combined with Kangaroo Positioning over ten days on the neuromotor behavior and clinical outcomes of stable preterm newborns hospitalized in a neonatal unit.

3.2 Specific objectives

To assess the general movements of preterm newborns who received the physiotherapy intervention protocol associated with the kangaroo position and compare them with the preterm control newborns before and after the protocol period, as well as at 12 weeks post-term.

To evaluate the weight gain in preterm newborns who received the physiotherapy intervention protocol associated with the kangaroo position compared to the preterm control newborns.

To analyze posture and muscle tone in preterm newborns who received the physiotherapy intervention protocol associated with the kangaroo position compared to preterm control newborns.

To assess the behavioral state in preterm newborns who received the physiotherapy intervention protocol associated with the kangaroo position compared to preterm control newborns.

To examine the time to establish and maintain breastfeeding in preterm newborns who received the physiotherapy intervention protocol associated with the kangaroo position compared to preterm control newborns.

To evaluate the length of hospital stay in preterm newborns who received the physiotherapy intervention protocol associated with the kangaroo position compared to preterm control newborns.

To assess respiratory distress scores in preterm newborns who received the physiotherapy intervention protocol associated with the kangaroo position compared to preterm control newborns.

To examine physiological parameters (vital signs) in preterm newborns who received the physiotherapy intervention protocol associated with the kangaroo position compared to preterm control newborns.

To compare the effects between preterm newborns who received the physiotherapy intervention protocol associated with the kangaroo position and preterm control newborns.

4 Methodology

4.1 Design

This project is characterized as a controlled, randomized, longitudinal, parallel-group clinical/therapeutic trial.

4.2 Participants

This study will involve 34 preterm newborns, born with a gestational age of 34 weeks or less, between 34 and 36 weeks and 6 days postmenstrual age, hospitalized in the Neonatal Intermediate Care Unit (UCIN). To estimate the minimum number of participants, considering a difference of at least 50% between the groups regarding the quality of general movements (GMs) after the intervention using the chi-square test, the suggested sample size is 13 participants per group (80% power; α = 5%). Considering potential losses during the follow-up, two groups of 17 participants will be included. The newborns will be randomly allocated into two groups: the experimental group (n=17), which will receive the physiotherapy intervention protocol associated with the kangaroo position, and the control group (n=17), which will receive only the kangaroo position.

4.3 Inclusion criteria

Preterm newborns will be included in the study if they meet the following criteria: gestational age at birth equal to or less than 34 weeks, more than 72 hours of postnatal life, hospitalized in the Neonatal Intermediate Care Unit (UCIN), clinically stable, general

movements with a poor repertoire, no need for invasive or non-invasive mechanical ventilation, may require oxygen support via nasal cannula, residents of Campo Grande-MS (Brazil).

Medical records, nursing notes, physiotherapy records, and the recommendations from neonatologists and the multidisciplinary team will be used as the reference for the selection or exclusion of patients based on the criteria described above.

4.4 Exclusion criteria

Preterm newborns will be excluded from the study if their gestational age at birth is equal to or greater than 34 weeks and 1 day, or if they have any of the following conditions: congenital malformations, chromosomal syndromes, active infections under treatment (with altered blood count or positive blood culture), neurological problems (such as grade III or IV intraventricular hemorrhage or brain malformations), neonatal asphyxia (with an Apgar score of less than 7 at the fifth minute), or congenital infections. Additionally, newborns will be excluded if they have any contraindications to performing the physiotherapy intervention protocol or the kangaroo position.

They will also be excluded if their parents or guardians do not consent to the newborn’s participation in the study or request their withdrawal. If the newborn experiences adverse reactions to the physiotherapy protocol or kangaroo positioning, such as clinical instability during the procedure (e.g., cyanosis, crying, lethargy, signs of pain, generalized erythema) or in the short and medium term (e.g., worsening of the clinical condition, feeding intolerance, or behavioral disturbances), they will be excluded. Moreover, newborns may also be excluded if complications arise that prevent the application of the protocol, such as the need for invasive or non-invasive mechanical ventilation, oxygen therapy via a Hood Box, or the requirement of surgical procedures.

4.5 Recruitment and data collection locations

The newborns will be recruited from the Neonatal Intermediate Care Units (UCIN) at the Hospital Universitário Maria Aparecida Pedrossian (HUMAP) of the Universidade Federal de Mato Grosso do Sul (UFMS) and from the UCIN at the Hospital Regional de Mato Grosso do Sul (HRMS). During the hospitalization period, data collection will take place in the UCIN itself, and after discharge, at the Laboratório de Estudos em Neuropediatria (LABEN) at the Clínica Escola Integrada of UFMS or in the home environment.

4.6 Equipment and materials

The following equipment will be used: pulse oximeter (Dixtal Biomédica®) to measure heart rate and peripheral oxygen levels, as well as a digital thermometer to measure body temperature before the intervention. A maternity gown will also be used for the mother, along with a moldable cotton band to contain the newborn next to the mother. These materials will be part of the routine usage in the Neonatal Intermediate Care Unit (UCIN).

The following assessment tools or scales will be used: Silverman-Andersen Scoring System (BSA), Prechtl's Qualitative Assessment of General Movements (GMA), adapted Brazelton' Scale, and the Neonatal Neuromotor Screening (TNN).

Two digital video cameras (Sony®), mounted on tripods, will be used to record the evaluations, one for the hospital setting and the other for the clinic setting. The videos will be transferred to a notebook computer and stored on an external hard drive (HD).

4.7 Assessment tools

4.7.1. Prechtl's General Movements Assessment (GMA)

General movements (GMs) make up the main spontaneous motor repertoire in the first months of a baby's life, being present from nine weeks of gestational age until approximately twenty weeks of postnatal life. They involve fluent and graceful movements with variable sequences of the neck, trunk, upper extremities, and lower extremities (PRECHTL, 1990; EINSPIELER; PRECHTL, 2005; EINSPIELER; PEHARZ; MARSCHIK, 2016). The presence, quality, and intensity of these movements provide information about the functional integrity of the newborn's central nervous system (HADDERS-ALGRA, 2014), suggesting that it is one of the most predictive tools for early detection of brain dysfunction before the corrected age of five months (EINSPIELER; PEHARZ; MARSCHIK, 2016; EINSPIELER; PRECHTL, 2005; EINSPIELER et al., 1997; NOVAK et al., 2017). The evaluation of GMs using the Prechtl method classifies the GMs as: a) Writhing Movements, which can be "normal," "poor repertoire," "cramped-synchronized," or "chaotic"; and b) Fidgety Movements, which can be normal, abnormal, or absent (EINSPIELER; PRECHTL, 2005; EINSPIELER et al., 1997). Manipulation of the baby is not allowed during the assessment, and newborns are positioned in a supine position, wearing only diapers or short clothes that allow freedom of movement (EINSPIELER; PRECHTL, 2005). The evaluation lasts about 5 minutes and must be filmed for analysis

4.7.2 Neonatal Neuromotor Screening (TNN)

A Neonatal Neuromotor Screening (TNN) was developed by Gonçalves (2012), based on the Dubowitz Neurological Signs Scale protocol (1970), and is used as a tool for the early diagnosis of neuromotor disorders in both term and preterm newborns (BIAZUS et al., 2016; GONÇALVES, 2010). It consists of 18 items and evaluates: posture, passive tone, active tone, primitive reflexes, and automatic body adjustment reactions. The application of the protocol should preferably be done when the baby is in an alert state, starting with the observation of posture, followed by the assessment of passive tone in the upper limbs, lower limbs, active tone, primitive reflexes, and alignment reactions. The final score is the sum of the scores obtained in each of the evaluated neurological signs items and is classified as hypotonia, normotonia, or hypertonia. The diagnostic conclusion is obtained by adding these scores along with the clinical data of the baby. It is easy to apply and takes, on average, 10 minutes, with the baby lying in the crib or examination table (GONÇALVES, 2012) (Figure 1)

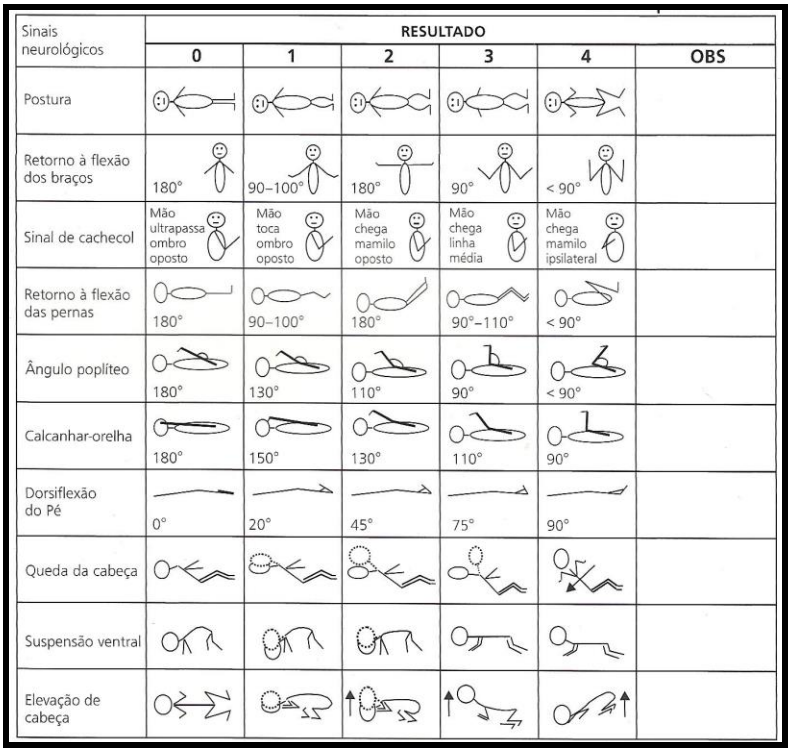

Figure 1. Illustration of the neurological signs of the Neonatal Neuromotor Screening. Source: Gonçalves (2012).

4.7.3 Brazelton’s Neonatal Behavioral State

The adapted Brazelton’s Neonatal Behavioral Assessment Scale was used to assess behavioral state. The scale categorizes the newborn's behavior based on their current state,

ranging from 1 to 6. State 1: deep sleep, still with steady breathing; State 2: light or active sleep, eyes closed with occasional body movements; State 3: drowsy, intermittently opening and closing the eyes; State 4: quietly alert, minimal physical activity; State 5: fully awake with energetic movements (active alert); State 6: crying (BRAZELTON et al., 1976).

4.7.4 Silverman-Andersen Score - BSA

To quantify the degree of respiratory distress/discomfort and monitor its evolution, the Silverman-Andersen Score (BSA) will be used. This is a clinical method that helps estimate the severity of pulmonary impairment. The score is calculated by summing the points (ranging from 0 to 2) assigned to the following parameters: intercostal retraction, xiphoid retraction, nasal flaring, and expiratory grunting. A total score below 5 indicates mild respiratory difficulty, while a score of 10 indicates the maximum degree of pulmonary dyspnea (SILVERMAN; ANDERSEN, 1956; BRAZIL, 2011b) (Figure 2).

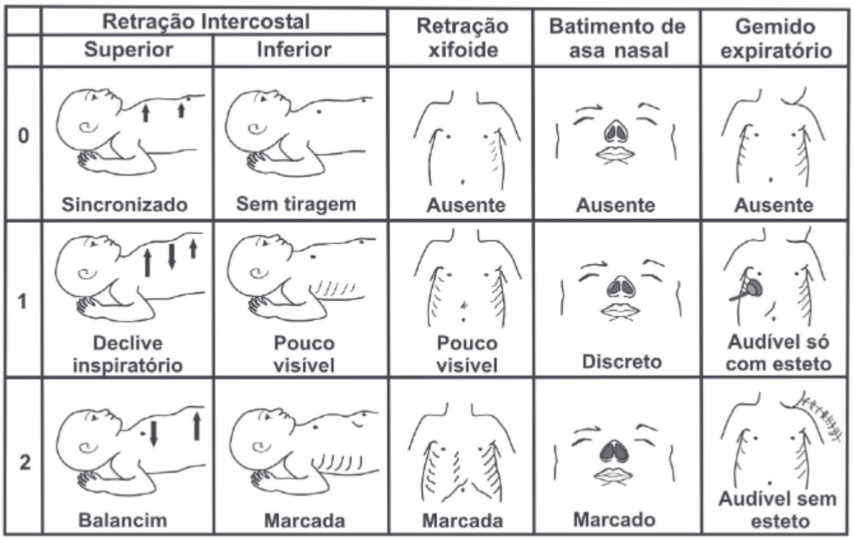

Figure 2. Illustration of the Silverman-Andersen Score. Source: Brazil (2011b).

4.8 Procedures

4.8.1 Procedures of ethics and study registration

The study will be submitted to the Ethics Committee of the Federal University of Mato Grosso do Sul, following the Guidelines and Regulatory Standards for Research Involving Human Beings (Res. 466/2012) from the National Health Council. Before the recruitment of subjects begins, the project will be registered in the Brazilian Clinical Trials Registry.

4.8.2 Procedures of randomization and allocation

The distribution of newborns into groups will be random, following a prior computational randomization in blocks of 4 babies for each group. The allocation to the groups will be concealed from the researchers involved through individual opaque, sealed, and sequentially numbered envelopes. The envelopes will be opened before the experimental or control protocol is performed, according to the inclusion of the babies in the study. The evaluations for GMs will be filmed and scored later, without identifying the allocation of the newborns in the groups, allowing for a subsequent blinded assessment.

4.8.3 Procedures for participant recruitment

First, newborns from the UCIN who meet the study's eligibility criteria will be recruited. Afterward, the mother or guardian will be approached and invited to participate, with an explanation of the study's objectives and procedures. If they agree, the participation of the newborn will be authorized by the mother or guardian through the signing of the Informed Consent Form.

4.8.4 Data collection and testing procedures

The clinical history data of the newborns will initially be recorded on a medical history form, collecting information regarding the newborn's eligibility criteria for the study. Data of both the newborn and the mothers will be obtained from medical records, nursing notes, physiotherapy records, and the child's health booklet.

The number of days from the start of the protocol until the establishment of breastfeeding will be recorded, along with the period of breastfeeding maintenance in days until hospital discharge. The continuation of exclusive breastfeeding after discharge will also be assessed at 12 weeks post-term. For these records, the mother and the multidisciplinary team will be consulted. Additionally, the daily body weight of the newborns will be recorded until the completion of the protocol, as well as the total length of hospital stay.

4.8.4.1. Assessments at the UCIN

The preterm newborns in both the experimental and control groups will be evaluated by the researcher in the NICU regarding respiratory discomfort at two time-points: I) before the

start of the protocol (experimental or control), with the baby still in the incubator/crib (Day 1); and II) after the completion of the protocol with the baby positioned in the incubator/crib (Day 15).

Physiological parameters (heart rate, respiratory rate, peripheral oxygen saturation, body temperature) and behavioral state will be assessed at three-time points daily: I) immediately before the protocol (experimental or control), with the baby still in the incubator/crib; II) 60 minutes after the start of the kangaroo position (experimental or control), while the baby remains in the position; and III) 30 minutes following, with the baby repositioned in the incubator/crib.

GMs and neuromotor screening (posture and tone) will be evaluated at two time-points: I) one day before the start of the protocol; and II) one day after the completion of the protocol.

4.8.4.2. Assessments after discharge

After hospital discharge, the GMs of babies from both groups will be assessed at 12 weeks post-term. GMA will be applied. The assessments will take place between feedings (after 1 to 1.5 hours) and should not coincide with vaccination days. The babies must be in an active alert state, i.e., state 4 (eyes open, no crying, but exhibiting gross movements) (BRAZELTON et al., 1976). The assessments will be conducted on a mat, with the baby preferably wearing only a diaper. If the baby is not cooperative, showing crying or restlessness, the assessment will be interrupted, the baby will be calmed, and the assessment will resume. If the baby remains restless, a new date will be scheduled.

The assessments will be filmed and scored later. The camera will be mounted on a tripod with sufficient distance and angle to capture the baby's full-body movements. Post-discharge assessments may take place through home visits or at the Neuropediatrics Study Laboratory (LABEN) at the Integrated School Clinic-UFMS, as per the parents'/guardians' preference. Training and agreement indices will be conducted between at least two experienced researchers/observers for assessment using the instruments, aiming for a minimum agreement index of 80%.

4.8.5 Experimental protocol

The experimental protocol will consist of a physiotherapeutic intervention protocol associated with the kangaroo position.

4.8.5.1 Physiotherapeutic intervention protocol

The physiotherapeutic intervention protocol will be applied only to the experimental group. The intervention will consist of the following handling techniques: I) Lumbosacral pumping; II) Posterior stretching; III) Cervical muscle stretching; IV) Thoraco-humeral dissociation; V) Therapeutic exercise to feel the head with the hands; and VI) Positioning in supine to allow spontaneous movements of the preterm newborn. The protocol begins with lumbosacral pumping while the newborn is in a supine position with the head slightly flexed. The therapist's right hand is positioned on the lumbosacral region, and the left hand supports the anterior pelvic region. Using the right hand's middle and ring fingers, the therapist gently pulls in a caudal direction (GONÇALVES, 2012; BUSQUET-VANDERHEYDEN, 2009); without losing pelvic retroversion, the newborn is moved to a lateral position, and posterior stretching is performed with the right hand on the sacral region and the left hand positioned under the occipital bone, performing subtle, passive traction on both sides of the vertebral axis during expiration (LIMA, 2012; BUSQUET-VANDERHEYDEN, 2009). After this handling, cervical stretching is performed with the therapist's right hand holding the shoulder and the left hand supporting the occipital and temporal regions. The newborn is raised to approximately 30°, the head and neck are gently slid to the right side, while the left shoulder is softly lowered. The shoulder is released, and the head is returned to the midline. The hands are reversed, and the movement is performed on the opposite side (DUARTE et al., 2011); immediately after this handling, thoraco-humeral dissociation is performed, where the therapist uses one hand to hold the shoulder region and makes circular movements in a postero-anterior direction (LIMA, 2012); in the exercise to feel the head with the hands, the newborn remains in a supine position, with the cervical spine straightened, trunk and lower limbs flexed, and the newborn's hands are moved to the parietal region, sliding the palms of the hands forward and laterally over the face, rubbing them in front (DUARTE et al., 2011); to finish, the newborn will be positioned in a supine position in the incubator/crib, allowing spontaneous movements with the nest slightly away. The protocol will last 15 minutes, and immediately afterward, the newborn will be placed in the kangaroo position with the mother for 60 minutes.

4.8.5.2 Kangaroo Position protocol

In the kangaroo position, the newborn should be placed only in a diaper in a vertical or elevated diagonal position, between the mother's breasts, with the head laterally positioned, upper and lower limbs in flexion and adduction. The mother should not wear a bra and should be dressed in a gown with a front opening. After positioning the baby, the mother should cover

him with the gown, and then the dyad should be secured with a moldable cotton band for additional safety (BRASIL, 2013).

The experimental protocol will begin when the baby reaches 34 weeks of postmenstrual age, has been alive for more than 72 hours, weighs over 1100g, and is clinically stable, respecting signs of stress (DUARTE et al., 2011). It will be conducted during the following 15 days. By the end of the protocol, each newborn will have received 15 sessions of the physiotherapeutic protocol combined with the kangaroo position. The interventions will be scheduled during the intervals between feeding times (oral or via tube). Stress factors such as noise and lighting will be minimized during the experimental and control protocols. The procedure will only be conducted after the newborn has been fed, waiting at least 60 minutes after feeding.

4.8.3 Control protocol

The newborns in the control group will undergo the same kangaroo positioning procedures described earlier but will not receive the physiotherapy intervention protocol. Thus, the newborn will be positioned in the incubator/crib before being placed in the kangaroo position with the mother.

4.9 Outcomes

4.9.1 Primary outcomes

- **Weight Gain**: daily body weight recording as a parameter for weight tracking of preterm newborns during the application of the protocol (experimental and control).
- **Neuromotor Behavior**: assessed by the classifications of GMs achieved using the GMA (EINSPIELER et al., 1997).

4.9.2 Secondary outcomes

- **Posture and Muscle Tone**: behavior regarding posture and muscle tone, assessed by the classification achieved using the neurological signs evaluation method from the neonatal neuromotor screening (GONÇALVES, 2012).
- **Behavioral State**: Behavioral state according to the adapted Brazelton scale (BRAZELTON et al., 1976).

- **Time to Establish Breastfeeding**: defined as the interval in days between the start of the protocol and successful breastfeeding at the breast. Success in breastfeeding will be considered according to the main key points for proper positioning and latch during the feeding: the baby's face facing the breast, with the nose at nipple level, the baby's body close to the mother's, with the baby's head and torso aligned; the baby well supported; more areola visible above the baby's mouth; mouth wide open; lower lip turned outward; and the chin touching the breast (BRASIL, 2011a).
- **Exclusive Breastfeeding Maintenance**: defined as the continuation of exclusive breastfeeding at corrected ages of 2 weeks post-term, 12 weeks post-term, and at 6 months, being dichotomous.
- **Hospitalization Time**: defined as the average number of days the newborn stays in the hospital environment.
- **Degree of Respiratory Distress** obtained by summing the score according to the BSA (SILVERMAN; ANDERSEN, 1956; BRASIL, 2011b) on each day of the protocol.
- **Physiological Parameters** (heart rate, respiratory rate, peripheral oxygen saturation, body temperature): vital signs evaluated and recorded before and after the protocol (experimental and control) each day.

4.10 Data Analysis

For the statistical analysis of the data, the statistical package SPSS 23.0 will be used. To analyze the dependent variables, the Levene's Test for Homogeneity of Variance and the Shapiro–Wilk Test for Normality will be applied. Once the assumptions are met, parametric and non-parametric tests may be applied depending on the homogeneity and normality conditions of the sample. Descriptive statistics will first be performed to characterize the sample. Treatment effects will be obtained by comparing the groups

To test differences between groups (treatment effects) for categorical variables (quality of GMs, behavioral state, success, and maintenance of breastfeeding), the chi-square test will be applied. To test differences between groups concerning body weight, the independent t-test will be used. For other continuous variables, t-tests or the Mann–Whitney test will be used to calculate differences between the groups. Adjusted means and 95% confidence intervals may also be calculated using the mixed linear model (GLM) for continuous variables, considering the interaction terms of group x days of evaluation.

To calculate the effect size of the intervention, a 95% confidence interval will be adopted, and either Cohen's d or r ($r = \text{z-score} / \sqrt{\text{total sample}}$) will be considered, depending

on whether parametric or non-parametric tests are used. A significance level of α=5% will be considered for all analyses.

5. Risks and Benefits

This study presents no physical or psychological risks to the newborn; however, there may be signs of sensory overload such as: color fluctuations (paleness, perioral cyanosis, among others), cardiorespiratory changes (bradycardia, irregular breathing, apnea, irregular respiratory rate), changes in state (hiccups, yawning, salivation, startles, crying, irritability), and signs of withdrawal when the newborn is handled. If signs of overload are detected, the handling should be interrupted to allow the newborn to organize, using maneuvers such as positioning and firm touch.

As for the benefits, the research will provide early stimulation/intervention to hospitalized preterm newborns, enhancing the baby's interaction with the environment through sensory-motor stimuli, which the baby interprets as pleasurable (approach signals). The kangaroo position is a safe and low-cost intervention, which can be performed in any hospital and provides the multidisciplinary team with an important additional resource in the care and attention of preterm newborns and their families, making them active participants in the treatment and promoting an efficient transition from the hospital environment to the home environment.

5. Budget

The study budget includes expenses for equipment, consumables, and printing, as shown in the following table:

Material/Equipment	Quantity	Unit Value	Total Value
Video camera	02	R$1500,00	R$3000,00
Tripod with head	02	R$700,00	R$1.400,00
Notebook	01	R$3.500,00	R$3.500,00
External hard drive (1TB)	01	R$440,00	R$440,00
Baby toys	07	R$15,00	R$105,00
Foam mat	02	R$80,00	R$160,00
Oximeter	01	R$ 2.000,00	R$ 2.000,00
Thermometer	01	R$ 15,00	R$ 15,00
Prints	2000	R$ 0,20	R$ 400,00
**TOTAL**	-	-	**R$11.020,00**

The equipment and materials have already been acquired and are part of the Laboratory of Neuropediatrics (LABEN). However, if necessary, the costs will be covered by the researcher's personal resources. The project will also be submitted to calls for proposals and funding agencies.

6. Project Schedule

**Steps**	**Start**	**Final**
Submission of the project to the Ethics and Research Committee of the Federal University of Mato Grosso do Sul	27/09/2018	27/09/2018
Literature update	27/09/2018	31/07/2021
Submission to REBEC (Brazilian Registry of Clinical Trials)	27/11/2018	30/11/2018
Start of recruitment and data collection	01/18/2018	30/06/2020
Writing of articles	01/08/2019	01/08/2021
Presentation at scientific events	01/09/2019	31/08/2021
Data tabulation	30/06/2020	30/07/2020
Statistical analysis and discussion of results	30/07/2020	30/10/2020
Pre-defense/Qualification	01/11/2020	30/11/2020
Discussion of results	30/10/2020	30/06/2021
Public presentation and defense	01/09/2021	30/09/2021
Submission of the final version	01/11/2021	30/11/2021

The schedule may be subject to changes due to unforeseen circumstances.

4. Relevance and Economic and Feasibility of the Project Execution

There is a great acceptance and application of early intervention and kangaroo positioning in Brazilian neonatal units due to the beneficial physiological effects observed in clinical practice. However, there is a scarcity of studies investigating the association of a physiotherapeutic intervention protocol, composed of maneuvers and therapeutic exercises, with kangaroo positioning. In this sense, the present study will contribute scientific support for evidence-based practice on this topic. Its results may be used to implement the

physiotherapeutic protocol associated with kangaroo positioning in the neonatal unit of the Hospital Universitário Maria Aparecida Pedrossian (HUMAP) – UFMS and in the Hospital Regional de Mato Grosso do Sul (HRMS), serving as a model for other hospitals in Brazil and worldwide, considering international publications and disclosures. Additionally, as this project will be carried out during a Doctoral Program, it will have an impact on human resource development at HUMAP - UFMS.

It is noteworthy that, taking into account the current approach to neonatal care humanization, as well as the recent Brazilian epidemiological need for more studies focused on early stimulation in hospitalized newborns at risk or with established neurological problems, the need for therapeutic investigations that help prevent and minimize morbidities becomes evident, favorably impacting the healthcare system's economy.

Regarding the financial feasibility of carrying out the project, the necessary materials and equipment for the research within the hospital, such as the oximeter and thermometer, have already been acquired and are individually used in the routine care of newborns in the Neonatal Unit at HUMAP-UFMS and HRMS - and will be used for the research. The video cameras, tripods, mats, notebook, external hard drive, and toys have already been acquired previously and belong to the Laboratory of Studies in Neuropediatrics (LABEN), coordinated by Prof. Dr. Daniele de Almeida Soares-Marangoni, making the research financially feasible.

References

ADAMS-CHAPMAN, I. Neurodevelopmental outcome of the late preterm infant. **Clinics in Perinatology**, v. 33, n. 4, p. 947-964, Dec. 2006.

ALBERTINE, K. H. Brain injury in chronically ventilated preterm neonates: collateral damage related to ventilation strategy. **Clinics in Perinatology**, v. 39, n. 3, p.727-40, Sept. 2012.

AMIN, S. B.; MERLE, K. S.; ORLANDO, M. S.; DALZELL, L. E.; GUILLET, R. Brainstem maturation in premature infants as a function of enteral feeding type. **Pediatrics**, v. 106, n. 2, p. 318-322, Sept. 2000.

ANAND, K. J. Effects of perinatal pain and stress. **Progress in Brain Research**, v. 122, p. 117-129, Feb. 2000.

AZEVEDO, V. M. G. O.; CALIXTO, A. F.; ABREU, L. R. Efeitos da posição canguru no sistema cardiorrespiratório de recém-nascidos pré-termo. *In*: ASSOCIAÇÃO BRASILEIRA DE FISIOTERAPIA CARDIORRESPIRATÓRIA E FISIOTERAPIA EM TERAPIA INTENSIVA; MARTINS, J. A., ANDRADE, L. B., RIBEIRO, S. N. S. (Org.). **PROFISIO**

Programa de atualização em fisioterapia pediátrica e neonatal: cardiorrespiratória e terapia intensiva. Ciclo 6. Porto Alegre: Artmed Panamericana, 2017. v. 3, p.119-149.

BARTOCCI, M.; BERGQVIST, L. L.; LAGERCRANTZ, H.; ANAND, K. J. Pain activates cortical areas in the preterm newborn brain. **Pain**, v. 122, n. 1-2, p. 109-117, May 2006.

BEGUM, E. A.; BONNO, M.; OHTANI, N.; YAMASHITA, S.; TANAKA, S.; YANAMOTO, H.; KAWAI, M.; KOMADA, Y. Cerebral oxygenation responses during kangaroo care in low birth weight infants. **BioMedCentral Pediatrics**, v. 51, n. 8, Nov. 2008.

BENZIES, K.M.; MAGILL-EVANS, J.E.; HAYDEN, K. A.; BALLANTYNE, M. Keu components of early intervention programs for preterm infants and their parents: a systematic review and meta-analysis. **BioMedCentral Pregnancy and Childbirth,** v. 13, suppl. 1, n. 10, Jan. 2013.

BERA, A.; GHOSH, J.; SINGH, A. K.; HAZRA, A.; SOM, T.; MUNIAN, D. Effect of kangaroo mother care on vital physiological parameters of the low birth weight infants. **Indian Journal of Community Medicine :** official publication of indian association of preventive & social medicine, v. 39, n. 4, p. 245-9, Oct./Dec. 2014.

BIAZUS, G. F.; KUPKE, C. C.; MATOS, S. S.; JANDT, S. R. Avaliação fisioterapêutica em neonatos que apresentaram asfixia perinatal e que foram submetidos à hipotermia terapêutica. **Revista Fisioterapia Saúde Funcional**, v. 5, n. 1, p. 59-68, Jan./Jul. 2016.

BLENCOWE, H.; COUSENS, S.; CHOU, D.; OESTERGAARD, M.; SAY, L.; MOLLER, A.; KINNEY, M. Born Too Soon: The global epidemiology of 15 million preterm births. **Reproductive Health**, v. 10, supl. 1, S2, p. 1-14, Nov. 2013.

BOUNDY, E. O., DASTJERDI, R.; SPIEGELMAN, D.; FAWZI, W. W.; MISSMER, S.A.; LIEBERMAN, E.; KAJEEPETA, S.; WALL, S.; CHAN, G. J. Kangaroo mother care and neonatal outcomes: a meta-analysis. **Pediatrics,** v. 137, n. 1, p. 1-16, Jan. 2016.

BRASIL. Ministério da Saúde**.** Secretaria de Atenção à Saúde. Departamento de Ações Programáticas Estratégicas. **Atenção humanizada ao recém-nascido de baixo peso:** Método Canguru – manual técnico. Brasília: Ministério da Saúde, 2017.

BRASIL. Ministério da Saúde. Secretaria de Atenção à Saúde. Departamento de Ações Programáticas Estratégicas. **Guia de orientações para o Método Canguru na atenção básica:** cuidado compartilhado. Brasília: Ministério da Saúde, 2016.

BRASIL. Ministério da Saúde. Secretaria de Atenção à Saúde**. Protocolo de atenção à saúde e resposta à ocorrência de microcefalia relacionada à infecção pelo vírus Zika.** Brasília: Ministério da Saúde, 2016.

BRASIL. Ministério da Saúde. Secretaria de Atenção à Saúde. Departamento de Ações Programáticas Estratégicas. Departamento de Atenção Básica. **Aleitamento materno, distribuição de leites e fórmulas infantis em estabelecimentos de saúde e a legislação**. Brasília : Ministério da Saúde, 2014.

BRASIL. Ministério da Saúde. Secretaria de Atenção à Saúde. Departamento de Ações Programáticas Estratégicas. **Atenção humanizada ao recém-nascido de baixo peso:** Método Canguru – Manual Técnico. Brasília: Ministério da Saúde, 2013.

BRASIL. Ministério da Saúde. Secretaria de Atenção à Saúde. Departamento de Ações Programáticas Estratégicas. **Atenção à saúde do recém-nascido:** guia para os profissionais de saúde. Cuidados com o recém nascido pré-termo. Brasília: Ministério da Saúde, 2011a.

BRASIL. Ministério da Saúde. Secretaria de Atenção à Saúde. Departamento de Ações Programáticas Estratégicas. **Atenção à saúde do recém-nascido:** guia para os profissionais de saúde. Problemas respiratórios, cardiocirculatórios, metabólicos, neurológicos, ortopédicos e dermatológicos. Brasília: Ministério da Saúde, 2011b.

BRASIL. Ministério da Saúde. **Saúde da Criança: nutrição infantil e, aleitamento materno e alimentação complementar.** Caderno de Atenção Básica, nº 23. Brasília, DF: 2009.

BRAZELTON, T.B.; PARKER, W.B.; ZUCKERMAN, B. **Importance of behavioral assessment of the neonate**. Curr. Probl. Pediatr. 1976, 7, 1–82.

BUSQUET-VANDERHEYDEN, M. **O bebê em suas mãos:** método das cadeias fisiológicas. 1. ed. Barueri : Manole, 2009.

CABRAL, L. A., SCHETTINO, R. C., POMPEU, L. P. Estratégias favorecedoras do desenvolvimento neuropsicomotor de recém-nascidos pré-termo: da UTI ao ambulatório de seguimento. *In*: ASSOCIAÇÃO BRASILEIRA DE FISIOTERAPIA CARDIORRESPIRATÓRIA E FISIOTERAPIA EM TERAPIA INTENSIVA; MARTINS, J. A., NICOLAU, C. M., ANDRADE, L. B. (Org.). **PROFISIO Programa de atualização em fisioterapia pediátrica e neonatal:** cardiorrespiratória e terapia intensiva. Ciclo 4. Porto Alegre: Artmed/Panamericana, 2015. v.1, p.95-127.

CALDAS, I. F. R. Desenvolvimento sociocomunicativo: interação mãe-bebê e fatores de risco. *In*: ASSOCIAÇÃO BRASILEIRA DE FISIOTERAPIA CARDIORRESPIRATÓRIA E FISIOTERAPIA EM TERAPIA INTENSIVA; MARTINS, J. A., SCHIVINSKI, C. I. S., RIBEIRO, S. N. S. (Org.). **PROFISIO Programa de atualização em fisioterapia pediátrica e neonatal:** cardiorrespiratória e terapia intensiva. Ciclo 6. Porto Alegre: Artmed/Panamericana, 2017. v.3, p. 29-50.

CALDAS, I. F. R. Fatores de risco e desenvolvimento sociocomunicativo em prematuros. **Revista Psicologia:** Teoria e Prática, São Paulo, v. 18, n. 2, p. 129-141, Maio/Ago. 2016.

CARBAJAL, R.; LENCLEN, R.; JUCIE, M.; PAUPE, A.; BARTON, B. A., ANAND, K. J. Morphine does not provide adequate analgesia for acute procedural pain among preterm neonates. **Pediatrics**, v.115, n. 6, p.1494-500, Mar. 2005.

CARVALHO, M. G. S., SIQUEIRA, J. C. F. Estimulação suplementar para recém-nascidos de alto risco. *In*: ASSOCIAÇÃO BRASILEIRA DE FISIOTERAPIA CARDIORRESPIRATÓRIA E FISIOTERAPIA EM TERAPIA INTENSIVA; NICOLAU, C. M., ANDRADE, L.B. (Org.). **PROFISIO Programa de atualização em fisioterapia pediátrica e neonatal:** cardiorrespiratória e terapia intensiva. Ciclo 2. Porto Alegre: Artmed/Panamericana, 2013. v. 3, p. 117-153.

DAWSON, G.; JONES, E. J. H.; MERKLE, K.; VENEMA, K.; LOWY, R.; FAJA, S.; KAMARA, D.; MURIAS, M.; GREENSON, J.; WINTER, J.; SMITH, M.; ROGERS, S. J. ; WEBB, S. J. Early Behavioral Intervention Is Associated With Normalized Brain Activity in Young Children With Autis. **Journal American Academy Child of Adolescent Psychiatry**, v. 51, n. 11, p.1150-1159, Nov. 2012.

DE GROOT, L. Posture and mobility in preterm infants. **Developmental Medicine e Child Neurology**, v. 42, n. 1, p. 65-8, Jan. 2000.

DE SCHUYMER, L.; DE GROOTE, I.; STRIANO, T.; STHAL, D.; ROYERES, H. Dyadic and triadic skills in preterm and full term infants: a longitudinal study in the first year. **Infant Behavior Development**., v. 34, p. 179-88, Feb. 2011.

DUARTE, D. T. R.; VANZO, L. C.; COPPO, M. R. C.; STOPIGLIA, M. S. Estimulação sensório-motora no recém-nascido. *In*: SARMENTO, G. J. V.; DE CARVALHO, F. A.; PEIXE, A. A. F. (Org.). **Fisioterapia Respiratória em Pediatria e Neonatologia**. 2. ed. Barueri: Manole, 2011.

DUBOWITZ, L. M.; DUBOWITZ, V.; GOLDERG, C. Clinical Assessment of gestational age in the newborn infant. **Journal Pediatrics**, v. 77, n. 1, p. 1-10, July 1970.

EINSPIELER, C.; PRECHT, H. F. R.; FERRARI, F.; CIONI, G.; BOS, A. F. The qualitative assessment of general movements in preterm, term and young infants - review of the methodology. **Early Human Development,** v. 50, n.1, p. 47-60, Nov. 1997.

EINSPIELER, C.; PEHARZ, R.; MARSCHIK, P. B. Fidgety movements – tiny in appearance, but huge in impact. **Jornal Pediatria.** v. 92, n. 3, supl. 1, p. 64-70, May/June 2016.

EINSPIELER, C.; PRECHTL, H. F. R. Prechtl's assessment of general movements: a diagnostic tool for the functional assessment of the young nervous system. **Mental Retardation and Developmental Disabilites Research Reviews**., v. 11, n. 1, p. 61-67, Apr. 2005.

FELDMAN, R., ROSENTHAL, Z., EIDELMAN, A. I. Maternal-preterm skin-to-skin contact enhances child physiologic organization and cognitive control across the first 10 years of life. **Biological Psychiatry,** v. 75, n. 1, p. 56-64, Jan. 2014.

FERREIRA, H.C., SANTOS, R.S. Posição prona em pediatria e neonatologia. *In*:ASSOCIAÇÃO BRASILEIRA DE FISIOTERAPIA CARDIORRESPIRATÓRIA E FISIOTERAPIA EM TERAPIA INTENSIVA; MARTINS, J. A., NICOLAU, C. M., ANDRADE, L. B. (Org.). **PROFISIO Programa de atualização em fisioterapia pediátrica e neonatal:** cardiorrespiratória e terapia intensiva. Ciclo 5. Porto Alegre: Artmed/Panamericana, 2016. v. 2, p. 9-57.

FOHE, K., KROPF, S., AVENARIUS, S. Skin-to-skin contact improves gas exchange in premature infants. **Journal Perinatology**, v. 5, n. 5, p. 311-5, July/Aug. 2000.

FORMIGA C. K. M. R.; PEDRAZZANI, E. S.; TUDELLA, E. **Intervenção Precoce com Bebês de Risco**. São Paulo: Atheneu, 2010.

FORMIGA, C. K. M. R.; LINHARES, M. B. M. Avaliação do desenvolvimento inicial de crianças nascidas pré-termo. **Revista da Escola de Enfermagem da USP**, v. 43, n. 2, p. 472-480, jun. 2009.

FORMIGA, C. K. M. R.; PEDRAZZANI, E. S.; TUDELLA, E. Desenvolvimento motor de lactentes pré-termo participantes de um programa de intervenção fisioterapêutica precoce. **Revista Brasileira de Fisioterapia,** v. 8, n. 3, p. 239-245, set./dez. 2004.

FORMIGA, C. K. M. R.; TUDELLA, E; MARQUES, L. R. FAGUNDES, R. R., DO AMARAL, L. E. F.; LINHARES, M. B. M. Desenvolvimento motor de bebês pré-termo e a termo de 0 a 6 meses de idade. **Pediatria Moderna**, v.51, n.12, p. 422-426, dez. 2015.

FORMIGA, C. K. M. R.; CEZAR, M. E. N.; LINHARES, M. B. M. Avaliação longitudinal do desenvolvimento motor e da habilidade de sentar em crianças nascidas prematuras. **Fisioterapia e Pesquisa**, v. 17, n. 2, p. 102-107, jun. 2010.

FRIEDRICH, L., CORSO, A. L., JONES, M. H. Prognóstico pulmonar em prematuros. **Jornal de Pediatria,** v. 81, Supl 1, p. S79-S88, 2005.

FUENTEFRIA, R. N.; SILVEIRA, R.C.; PROCIANOY, R.S. Motor development of preterm infants assessed by the Alberta Infant Motor Scale: systematic review article. **Jornal de Pediatria**, v. 93, n. 4, p. 328-342, July/Aug. 2017.

GARCIA, C.; GEPHART, S. M. The effectiveness of early intervention programs for NICU graduates. **Advances in Neonatal Care**, v.13, n. 4, p. 272-8, Aug. 2013.

GARCIA, J. M.; GHERPELLI, J. L.; LEONE, C. R. The role of spontaneous general movement assessment in the neurological outcome of cerebral lesions in preterm infants. **Jornal de Pediatria**, v. 80, n. 4, p. 296-304, July/Aug. 2004.

GASPARDO, M. C., LINHARES, M. B. M., MARTINEZ, F. E. A eficácia da sacarose no alívio de dor em neonatos: revisão sistemática da literatura. **Jornal de Pediatria**, v. 81, n. 6, p. 435-442, Nov. 2005.

GONÇALVES, M. C. P. Practicality and effectiveness of the physical examination protocol for neonatal neuromotor scanning. **Fiep Bulletin.** Special Edition- Article II, v. 80, p. 431-35, 2010.

GONÇALVES, M. C. P. **Prematuridade:** desenvolvimento neurológico e motor: avaliação e tratamento. Rio de Janeiro: Revinter, 2012.

GRENIER, I. R.; BIGSBY, R.; VERGARA, E. R.; LESTER, B. M. Comparison of motor self-regulatory and stress behaviors of preterm infants across body positions. **The American Journal of Occupational Therapy**, v. 57, n. 3, p. 289-97, May/June. 2003.

GRUNAU, R. Early pain in preterm infants: a model of long-term effects. **Clinics in Perinatology**, v.29, n. 3, p. 373-394, Oct. 2002.

GRYBOSKI, J. D. Suck and swallow in the premature infant. **Pediatrics**, v. 43, n. 1, p. 96-102, Jan. 1969.

GUIHARD-COSTA, A. M.; LARROCHE, J. C. Differential growth between the fetal brain and its infratentorial part. **Early Human Development**, v. 23, n. 1, p. 27-40, June 1990.

GUINCHAT. V.; THORSEN, P.; LAURENT, C.; CANS, C.; BODEAU, N.; COHEN, D. Pre, peri- and neonatal risk factors for autism**. Acta Obstetriciaet et Gynecologica Scandinavica,** v. 91, n. 3, p. 287-300, Mar. 2012.

HADDERS-ALGRA, M. Early diagnosis and early intervention in cerebral palsy**. Frontiers Neurology**, v. 5, n. 185, p. 1-13, Sept. 2014.

HAGBERG, H., JACOBSSON, B. Brain injury in preterm infants - what can the obstetrician do? **Early Human Development**, v. 81, n. 3, p. 231–235, Mar. 2005.

HEATHCOCK, J. C.; LOBO, M.; GALLOWAY, J. C. Movement training advances the emergence of reaching in infants born at less than 33 weeks of gestational age. **Physical Therapy**, v. 88, n. 3, p. 310–322, Mar. 2008.

HUNT, F. The importance of kangaroo care on infant oxygen saturation levels and bonding. **Journal of Neonatal Nursing,** v.14, n. 2, p. 47-51, Apr. 2008.

JOHNSTON, C.C. Kangaroo mother care diminishes pain from heel lance in very preterm neonates: a crossover trial. **BMC Pediatrics**, v. 8, n. 13, p. -9, Apr. 2008.

KINNEY, H.C. The near-term (late preterm) human brain and risk for periventricular leukomalacia: A review. **Seminars in Perinatology**, v. 30, n. 2, p. 81-88, Apr. 2006.

LAGO, P.; GARETTI, E.; MERAZZI, D.; PIERAGOSTINI, L.; ÂNCORA, G. PIRELLI, A.; BELLIENI, C. V. Pain Study Group of the Italian Society of Neonatology. Guideline for procedural pain in the newborn. **Acta Paediatrica**., v. 98, n. 6, p. 932-939, June 2009.

LAMY-FILHO, F.; DA SILVA, A. A. M.; LAMY, Z. C.; GOMES, M. A. S. M.; MOREIRA, M. E. L.; GRUPO DE AVALIAÇÃO DO MÉTODO CANGURU; REDE BRASILEIRA DE PESQUISAS NEONATAIS . Evaluation of the neonatal outcomes of the kangaroo mother method in Brazil. **The Journal of Pediatrics**, v.84, n. 5, p. 428-435, Sept./Oct. 2008.

LEAL, M. C.; ESTEVES-PEREIRA, A. P.; NAKAMURA-PEREIRA, M.; TORRES, J. A; TEMA-FILHA, M.; DOMINGUES, R. M. S. M.; DIAS, M. A. B.; MOREIRA, M. E.; GAMA, S. G. Prevalence and risk factors related to preterm birth in Brazil. **Reproductive Health**, v.13, Suppl 3, n. 127, p. 163-174, Oct. 2016.

LEMOS, A.; MAUX, D. A. S. X.; PAIVA, G. S. Assistência ventilatória em patologias neonatais. *In*: ASSOCIAÇÃO BRASILEIRA DE FISIOTERAPIA CARDIORRESPIRATÓRIA E FISIOTERAPIA EM TERAPIA INTENSIVA; NICOLAU, C. M.; ANDRADE, L. B. (Org.). **PROFISIO Programa de atualização em fisioterapia pediátrica e neonatal:** cardiorrespiratória e terapia intensiva. Ciclo 2. Porto Alegre: Artmed/Panamericana, 2013. v.3, p. 47-87.

LIEM, K. D.; GREISEN, G. Monitoring of cerebral haemodynamics in newborn infants. **Early Human Development**., v. 86, n. 3, p. 155-8, Mar. 2010.

LIMA, M. P. Bases do Método Reequilíbrio Tóraco-abdominal. *In*: SARMENTO, G. J. V. (Org.). **O ABC da Fisioterapia Respiratória**. Barueri: Manole, 2009. p. 197-211.

LUDINGTON-HOE, S. M.; ANDERSON, G. C.; SIMPSON, S.; HOLLINGSEAD, A.; ARGOTE, L. A.; REY, H. Birth-related fatigue in 34-36-week preterm neonates: rapid recove y with very early kangaroo (Skin-to-Skin) care. **Journal Obstetric Gynecologic and Neonatal Nursing**, v. 28, n. 1, p. 94-103, Jan./Feb. 1999.

LUDINGTON-HOE, S. M, FERREIRA,C.N, GOLDSTEIN,M.R. Kangaroo care with a ventilated preterm infant. **Acta Paediatrica**, v. 87, n. 6, p. 711-3, Jun. 1998.

MARLOW, N. Neurocognitive outcome after very preterm birth. **Archives Disease Childhood Fetal and Neonatal**, v. 89, n. 3, p. 224-8, May 2004.

NEWNHAM, C. A., INDER, T. E., MILGROM, J. Measuring preterm cumulative stressors within the NICU: The neonatal infant stressor scale. **Early Human Development**, v. 85, n. 9, p. 549-555, Sept. 2009.

NOVAK, I.; MORGAN, C.; ADDE, L.; LACKMAN, J.; BOYD, R.N.; BRUNSTROM-HERNANDEZ, J.; CIONI, G.; DAMIANO, D.; DARRAH, J.; ELIASSON, A.; DE VRIES, L. S.; EINSPIELER, C.; FAHEY, M.; FEHLINGS, D.; FERRIERO, D. M.; FETTERS, L.; FIORI, S.; FORSSBERG, H.; GORDON, A. M.; GREAVES, S.; GUZZETA, A. HADDERS-ALGRA, M.; HARBOURNE, R. KAKOOZA-MWESIGE, A.; KARLSSON, P.; KRUMLINDE-SUNDHOLM, L.; LATAL, B.; LOUGHRAN-FOWLDS, A.; MAITRE, N.; MCINTYRE, S.; NORITZ, G.; PENNINGTON, L.; ROMEO, D. M.; SHEPHERD, R.; SPITTLE, A. J.; THORNTON, M.; VALENTINE, J.; WALKER, K.; WHITE, R.; BADAWI, N. Early, Accurate Diagnosis and Early Intervention in Cerebral Palsy: Advances in Diagnosis and Treatment. **JAMA Pediatrics**, v. 171, n. 9, p. 897-907, Sept. 2017.

OBERG, G.K.; CAMPBELL, S. K.; GIROLAMI, G. L.; USTAD, T.; JORGENSEN, L.; KAARESEN, P. I. Study protocol: an early intervention program to improve motor outcome in preterm infants: a randomized controlled trial and a qualitative study of physiotherapy performance and parental experiences. **BMC Pediatrics,** v. 15, n. 12, p. 1-9, Feb. 2012.

ODD, D.; EVANS, D.; EMOND, A. Preterm birth, age at school entry and educational performance. **PLoS One,** v. 8, n. 10, Oct. 2013.

PADILHA, H. F.; STEIDL, E. M. S.; BRAZ, M. M. Efeitos do método mãe-canguru em recém-nascidos pré-termo. **Fisioterapia Brasil**, v. 15, n. 2, Mar./Abr. 2014.

PIN, T.W.; DARRER, T.; ELDRIDGE, B.; GALEA, M. P. Motor development from 4 to 8 months corrected age in infants born at or less than 29 weeks' gestation. **Developmental Medicine and Child Neurology,** v. 51, p. 739-45, Mar. 2009.

PIPER, M. C.; DARRAH. J. **Motor assessment of the developing infant**. Philadelphia: W. B. Saunders Company, 1994.

PITCHER, J. B.; SCHNEIDER, L.A.; BURNS, N. R.; DRYSDALE, J. L.; HIGGINS, R. D.; NETTELBECK, T. J.; HASLAN, R. R.; ROBINSON, J. S. Reduced corticomotor excitability and motor skills development in children born preterm. **The Journal of Physiology,** v. 590, n. 22, p. 5827-5844, Nov. 2012.

PRECHTL, H. F. Qualitative changes of spontaneous movements in fetus and preterm infant are a marker of neurological dysfunction. **Early Human Development**, v. 23, n. 3, p. 151-158, Sept. 1990.

RAMACHANDRAN, S.; DUTTA, S. Early developmental care interventions of preterm very low birth weight infants. **Indian Pediatrics**, v. 50, n. 8, p.765-770, Aug. 2013.

REICHERT, A. P. S.; LINS, R. N. P.; COLLET, N. Humanização do cuidado da UTI Neonatal. **Revista Eletrônica de Enfermagem**, v. 9, n. 1, p. 200-213, Jan./Fev. 2007.

REINAUX, C. M. A. **Evolução motora de recém-nascidos pré-termo submetidos ao método mãe-canguru.** 2005. 149 f. Dissertação (Mestrado em Fisioterapia) - Universidade Metodista de Piracicaba, Piracicaba, 2005.

SACCANI, R., VALENTINE, N. C. Reference curves for the Brazilian Alberta Infant Motor Scale: percentiles for clinical description and follow-up over time. **Jornal de Pediatria**, v. 88, n. 1, p. 40-47, Jan./Feb. 2012.

SACCANI, R.; VALENTINI, N. C.; PEREIRA, K. R. G. New Brazilian developmental curves and reference values for the Alberta infant motor scale. **Infant Behavior & Development**, v. 45, p. 38-46, Nov. 2016.

SANTORO JÚNIOR, W.; MARTINEZ, F. E. Effect of intervention on the rates of breastfeeding of very low birth weight newborns. **Jornal de Pediatria**, v. 83, n. 6, p. 541-546, Nov./Dez. 2007.

SANTOS, M. H.; AZEVEDO FILHO, F. M. Benefícios do método mãe canguru em recém-nascidos pré-termo ou baixo peso: uma revisão de literatura. **Universitas**: Ciências da Saúde, v.14, n. 1, p. 67-76, Jan./Jun. 2016.

SCHNEIDER, C.; CHARPAK, N.; RUIZ-PELAEZ, J.G.; TESSIER, R. Cerebral motor function in very premature-at-birth adolescents: a brain stimulation exploration of kangaroo mother care effects. **Acta Paediatrica**, v. 101, n. 10, p. 1045-1053, Oct. 2012.

SHAH, P. E.; ROBBINS, N.; COELHO, R. B.; POEHIMANN, J. The paradox of prematurity: the behavioral vulnerability of late preterm infants and the cognitive susceptibility of very preterm infants at 36 months post-term. **Infant Behavior & Development,** v. 36, n. 1, p. 50-62, Feb. 2013.

SHAIKH, A. G.; NAMRATA, P. Effectiveness of Massage Therapy as an Adjunct to Kangaroo Mother Care on Physiological and Behavioural Status of Low Birth Weight Preterm Infants. **Indian Journal of Physiotherapy and Occupational Therapy**, v. 11, n. 2, p. 103-8, Apr./June 2017.

SHAREK, P. J.; POWERS, R.; KOEHN, A.; ANAND, K. J. Evaluation and development of potentially better practices to improve pain management of neonates. **Pediatrics,** v. 118, Suppl 2, p. 78-86, Nov. 2006.

SHONKOFF, J. P.; GARNER, A. S. American Academy of Pediatrics Technical Report. The lifelong effects of early childhood adversity and toxic stress. **Pediatrics**, v. 129, n. 1, p. 232-244, Jan. 2012.

SILVA, E. S.; NUNES, M. L. The influence of gestational age and birth weight in the clinical assessment of the muscle tone of healthy term and preterm newborns. **Arquivos de Neuropsiquiatria**, v. 63, n. 4, p. 956-962, Dez. 2005.

SILVERMAN, W. A.; ANDERSEN, D. H. A controlled clinical trial of effects of water mist on obstructive respiratory signs, death rate and necropsy findings among premature infants. **Pediatrics**, v. 17, n. 1, p. 1–10, Jan. 1956.

SIMONS, S.H.; VAN DIJK, M.; ANAND, K. S.; ROOFTHOOFT, D; VAN LINGEN, R. A.; TIBBOEL, D. Do we still hurt newborn babies? A prospective study of procedural pain and analgesia in neonates. **Archives Pediatrics Adolescent Medicine**., v. 157, n. 11, p. 1058-64, Nov. 2003.

SISTEMA DE INFORMAÇÕES DE NASCIDOS VIVOS (SINASC). **Estatísticas vitais,** 2017. Disponível em: <http://tabnet.datasus.gov.br/cgi/deftohtm.exe? sinasc/cnv/nvms.def>. Acesso em: 31 out. 2017.

SOARES-MARANGONI, D. A.; TEDESCO, N. M.; NASCIMENTO, A. L.; DE ALMEIDA, P. R.; PEREIRA, C. N. S. General movements and motor outcomes in two infants exposed to Zika virs: brief report. **Developmental Neurorehabilitation**, 2018. DOI: 10.1080/17518423.2018.1437843

SOARES, D. A.; VON HOFSTEN, C.; TUDELLA, E. Development of exploratory behavior in late preterm infants. **Infant Behavior and Development,** v. 35, n. 4, p. 912–915, Dec. 2012.

SOARES, D. A.; CUNHA, A. C.; TUDELLA, E. Differences between late preterm and full-term infants: Comparing effects of a short bout of practice on early reaching behavior. **Research in Developmental Disabilities**, v. 35, n. 11, p. 3096-3107, Nov. 2014.

SOUKKA, H.; GRONROOS, L.; LEPPASALO, J.; LEHTONEN. L. The effects of skin-to skin care on the diaphragmatic electrical activity in preterm infants. **Early Human Development**, v. 90, n. 9, p. 531-534, Sept. 2014.

SPITTLE, A.; ORTON, J.; ANDERSON, P.; BOYD, R.; DOYLE, L.W. Early developmental intervention programmes post-hospital discharge to prevent motor and cognitive impairments in preterm infants. **Cochrane Database Systematic Reviews,** n. 12, p. 1-103, Oct. 2012.

SPITTLE, A.; ORTON, J.; ANDERSON, P.; BOYD, R.; DOYLE, L.W. Early developmental intervention programmes provided post hospital discharge to prevent motor and cognitive impairment in preterm infants. **Cochrane Database Systematic Reviews**, n.11, p. 1-110, 2015.

VALENTINI, N. C; SACCANI, R. Brazilian Validation of the Alberta Infant Motor Scale. **Physical Therapy**, v. 92, n. 3, p. 440-7, Mar. 2012.

VANDERVEEN, J. A.; BASSLER, D.; ROBERTSON, C. M. T.; KIRPALANI, H. Early interventions involving parents to improve neurodevelopmental outcomes of premature infants: a meta-analysis. **Journal Perinatology**, v. 29, n. 5, p. 343-351, May 2009.

VAIVRE-DOURET, L.; ENNOURI, K.; JRAD, I.; GARREC, C.; PAPIERNIK, E. Effect of positioning on the incidence of abnormalities of muscle tone in low-risk, preterm infants. **European Journal of Paediatric Neurology**, v. 8, n. 1, p. 21-34, Jan. 2004.

VENTURELLA, C. B; ZANANDREA, G.; SACCANI, R.; VALENTINI, N. C. Desenvolvimento motor de crianças entre 0 e 18 meses de idade: Diferenças entre os sexos. **Motricidade**, v. 9, n. 2, p. 3-12, Abr. 2013.

WORLD HEALTH ORGANIZATION (WHO). Library Cataloguing in Publication Data. **International statistical classification of diseases and related health problems**. - 10th rev., v. 2, WAO, 2010.
