# Supplementary material for: Effect of sensory-motor intervention associated with skin-to-skin contact on neuromotor and clinical outcomes of preterm newborns: A randomized controlled trial
Source: PLoS One. 2025 Sep 12;20(9):e0332269. doi: 10.1371/journal.pone.0332269 (PMC12431241; doi:10.1371/journal.pone.0332269)
Supplement: S2 File — (PDF) [file pone.0332269.s005.pdf]

Universidade Federal de Mato Grosso do Sul
Programa de Pós-Graduação em Saúde e Desenvolvimento da Região Centro-Oeste

Mariane de Oliveira Nunes Reco

Projeto Submetido ao Comitê de Ética

Repercussões Longitudinais de um Protocolo de Intervenção Fisioterapêutica Associado ao Posicionamento Canguru em Recém-Nascidos Pré-Termo: Ensaio Clínico Controlado Randomizado

Campo Grande
2018

Mariane de Oliveira Nunes Reco

Projeto Submetido ao Comitê de Ética

Repercussões Longitudinais de um Protocolo de Intervenção Fisioterapêutica Associado ao Posicionamento Canguru em Recém-Nascidos Pré-Termo: Ensaio Clínico Controlado Randomizado

Projeto submetido a Comitê de Ética e apresentado ao Programa de Pós-Graduação em Saúde e Desenvolvimento na Região Centro-Oeste da Universidade Federal de Mato Grosso do Sul como requisito para o processo seletivo de Doutorado.

Orientadora: Prof[a]. Dr[a]. Daniele de Almeida Soares Marangoni

Campo Grande
2018

Resumo

Recém-nascidos pré-termo são vulneráveis a afecções perinatais devido a sua imaturidade orgânica e fatores externos associados. Essa associação pode levar a comprometimentos neurológicos, interferindo no desenvolvimento motor, na mecânica respiratória e no estabelecimento da amamentação. Considerando a abordagem atual de humanização do cuidado neonatal, bem como a recente necessidade epidemiológica brasileira de estudos voltados á intervenção precoce em recém-nascidos hospitalizados com risco ou alterações neurológicas estabelecidas, torna-se clara a necessidade de investigações terapêuticas que colaborem para prevenir e minimizar morbidades nesta população. Este estudo tem como objetivo primário verificar os efeitos, em curto e médio prazos, de um protocolo de intervenção fisioterapêutica associado a posição canguru no comportamento neuromotor e desfechos clínicos de recém-nascidos pré-termo em unidade neonatal. Será realizado um ensaio clínico randomizado, com desenho de grupos paralelos e razão de distribuição balanceada e participarão deste estudo 34 recém-nascidos pré-termo, com mais de 72 horas de vida pós-natal, internados em Unidades de Cuidados Intermediários Neonatal, com quadro clínico estável. Eles serão subdivididos em dois grupos de forma aleatória: a) grupo experimental (GE), que receberá o protocolo de intervenção fisioterapêutica associado ao posicionamento canguru; e b) grupo controle (GC), que receberá somente a posição canguru. O protocolo será realizado durante 10 dias consecutivos, com duração de 15 minutos em cada sessão do protocolo de intervenção fisioterapêutica e 60 minutos de posição canguru, no período de 34 a 36 semanas e 6 dias de idade pós-menstrual. Serão considerados desfechos primários: ganho de peso ao término do protocolo; movimentos gereralizados de Prechtl com 36 semanas pós-menstrual e 12 semanas pós-termo. Postura e tônus muscular, estado comportamental, tempo de estabelecimento e manutenção da amamentação, tempo de hospitalização e sinais vitais serão desfechos secundários. O presente estudo oferecerá fundamentação experimental para prática baseada em evidências e seus resultados poderão ser utilizados para guiar protocolos de intervenção viáveis para as rotinas das equipes nas unidades neonatais de hospitais brasileiros.

Palavras-chaves: Estimulação precoce. Método canguru. Recém-nascido prematuro. Desenvolvimento infantil.

1 Introdução

O nascimento prematuro ocorre quando o parto acontece com menos de 37 semanas completas de gestação ou menos de 259 dias, contados a partir do primeiro dia de menstruação (WORLD HEALTH ORGANIZATION, 2010), resultando no nascimento de bebês com imaturidade biológica para a vida extrauterina.

A frequência de parto prematuro tem aumentado em todo o mundo, inclusive no Brasil. A prevalência global de parto prematuro no país para o período 2011-2012 foi estimada em 11,5% (LEAL *et al*., 2016), sendo o mesmo posicionado, segundo a Organização Mundial de Saúde (BLENCOWE, 2013), entre os dez países que mais contribuem para o aumento do número de recém-nascidos pré-termo no mundo. A região Centro-Oeste ocupa a quinta posição no hanking das regiões brasileiras, com incidência de 6,2% de nascimentos prematuros no país (LEAL *et al*., 2016). O estado de Mato Grosso do Sul apresentou em 2015 uma proporção de 11,8% de partos prematuros (SINASC, 2017). Esses dados despertam atenção das políticas públicas pois as afecções perinatais responsáveis pela mortalidade infantil no Brasil são especialmente comuns nessa população de recém-nascidos (BRASIL, 2011a).

A idade gestacional e o peso ao nascimento são algumas das variáveis biológicas que abrangem o nascimento do recém-nascido. Em especial, recém-nascidos pré-termo com baixo peso ao nascimento (< 2500 g), são vulneráveis a afecções perinatais devido a uma série de fatores orgânicos e externos associados. A imaturidade orgânica, principalmente dos sistemas pulmonar e neurológico, compromete as trocas gasosas potencializando o risco de óbito e morbidades relacionadas a comprometimentos neurológicos (BRASIL, 2011a). A ineficiente produção de surfactante (LEMOS; MAUX; PAIVA; 2013) associada à imaturidade estrutural impõe importantes limitações à respiração extrauterina precoce (FRIEDRICH; CORSO; JONES, 2005). Nesses recém-nascidos podem ocorrer variabilidade nas trocas gasosas levando a períodos de hipóxia e de hiperóxia, de hipo ou de hipercapnia, alterando a capacidade autorregulatória do fluxo sanguíneo cerebral aumentando o risco de lesões cerebrais (LIEM; GREISEN, 2010). Tal fato pode comprometer a oxigenação encefálica e, consequentemente, a organização e funcionamento do sistema nervoso central em período de grande desenvolvimento (ALBERTINE, 2012; HAGBERG; JACOBSSON, 2005).

A imaturidade do sistema neuromotor é outra barreira intrínseca ao desenvolvimento normal do recém-nascido pré-termo. Em decorrência desta imaturidade e de fatores ambientais, o recém-nascido pré-termo pode não ter a experiência de movimento adequada, e

sem experiências adequadas é menos capaz de perceber estímulos e, com uma percepção alterada, é menos capaz de aprender (DE GROOT, 2000) e de realizar movimentos espontâneos contra a gravidade (GRENIER *et al*., 2003; VAIVRE-DOURET *et al*., 2004). O feto humano possui padrões de movimentos específicos gerados de forma endógena que emergem entre a 9ª e a 12ª semana pós-menstrual e continuam após o nascimento, independente de quando ocorre o mesmo. Fazem parte desse repertório motor espontâneo precoce os chamados movimentos generalizados, presentes do início da vida fetal até o 5º mês após idade de termo (EINSPIELER; PRECHTL, 2005; EINSPIELER *et al*., 1997). Durante o período pré-termo, os movimentos generalizados normais envolvem o corpo como um todo, ocorrem com certa frequência e permanecem tempo suficiente para serem observados. Possuem uma sequência variável de movimentos dos braços, pernas, pescoço e tronco, aumentam e diminuem de intensidade, força e velocidade e apresentam início e fim graduais. As rotações ao longo do eixo dos membros e as pequenas mudanças na direção tornam estes movimentos fluentes e elegantes e dão a impressão de complexidade e variabilidade (EINSPIELER; PRECHTL, 2005). Já no período de termo até a 6ª ou 9ª semana pós-termo, os movimentos generalizados apresentam uma forma elíptica e são caracterizados por movimentos de menor amplitude, com velocidade moderada, mais próximos da linha média, chamados de *writhing movements*. Do período pré-termo até a 9ª semana pós-termo, os padrões motores que diferem dos movimentos generalizados descritos são chamados de anormais e podem ser classificados em *poor repertorie*, apresentando sequência de movimento monótona; em *cramped-synchronized*, movimentos rígidos com ausência do caráter normal suave e fluente ou; *chaotic*, movimentos de grande amplitude, desprovidos de fluência e elegância (EINSPIELER; PRECHTL, 2005; EINSPIELER *et al*., 1997).

Por volta da 6ª a 9ª semana de idade pós-termo ocorre uma mudança nesse padrão de movimento de membros, tronco e cabeça, e os *writhing movements* gradualmente desaparecem enquanto emergem movimentos com amplitude mais baixa, velocidade moderada, aceleração variável, em todas as direções, presentes continuamente enquanto o bebê está acordado, chamados de *fidgety movements*. Estes movimentos normalmente estão contínuos e mais evidentes da 9ª a 15ª-20ª semana pós-termo e desaparecem de forma gradual quando os movimentos intencionais começam a aparecer. Movimentos de *fidgety* anormais ou ausentes indicam um maior risco de disfunções neurológicas posteriores, ao passo que, movimentos *fidgety* normais são preditivos de desenvolvimento normal (EINSPIELER; PEHARZ; MARSCHIK, 2016; EINSPIELER; PRECHTL, 2005; EINSPIELER *et al*., 1997). Recém-nascidos pré-termo, em decorrência de fatores biológicos e ambientais, podem apresentar uma

limitação da movimentação espontânea ao longo do período de permanência em unidade de cuidados neonatais (BRACEWELL, MARLOW, 2002) quando comparados a recém-nascidos a termo (FALLANG *et al*., 2005). Estes recém-nascidos podem apresentar uma sequência de movimentação monótona, rígida ou irregular em consequência de seu controle postural prejudicado (FALLANG *et al*, 2005), perdendo a característica de fluência, complexidade e variabilidade dos movimentos espontâneos, bem como apresentar movimentos *fidgety* menos evidentes (EINSPIELER; PEHARZ; MARSCHIK, 2016).

Associados aos fatores orgânicos, os fatores externos ou ambientais necessários à assistência clínica do recém-nascido pré-termo nas unidades de terapias intensivas podem provocar déficits nos aspectos motores, sensoriais, mentais e emocionais. Fatores externos como separação materna e estimulação sensorial inadequada, ora com estímulos insuficientes, ora excessivos, potencializam os riscos para morbidades e repercutem negativamente para o correto desenvolvimento.

Todas as referências de relações iniciais da mãe e recém-nascido pré-termo ficam alteradas frente à separação da díade em unidade neonatal. Esta separação limita a formação do vínculo mãe-filho, dificulta o processo de amamentação (AMIN *et al*., 2000; BRASIL, 2009; SANTORO JUNIOR; MARTINEZ, 2007) e interfere na organização e desenvolvimento de circuitos cerebrais envolvidos na linguagem e na comunicação social (CALDAS, 2016). Desta forma, o recém-nascido pré-termo está inicialmente suscetível à privação dos benefícios do aleitamento materno e à alterações sociocomunicativas para seu desenvolvimento.

Como agravante, os recém-nascidos pré-termo, em um período inicial de desenvolvimento do sistema nervoso central, são normalmente submetidos a procedimentos dolorosos e de estimulação sensorial excessiva rotineiramente nas unidades neonatais (CARBAJAL *et al*., 2005; SHAREK *et al*, 2006; SIMONS *et al*., 2003). Tais estímulos podem levar a alterações no processamento da dor, a alterações comportamentais e a diminuição do volume de áreas sensoriais do cérebro (GASPARDO; LINHARES; MARTINEZ, 2005; LAGO *et al*., 2009; NEWNHAM; INDER; MILGROM, 2009).

Na trajetória do desenvolvimento, além dos fatores de risco, os quais expõem os recém-nascidos a múltiplos e contínuos eventos adversos, também estão presentes os fatores de proteção ao desenvolvimento. Quando a resposta a esses eventos é frequente, intensa ou duradoura e associada à ausência ou escassez de fatores protetores, o impacto negativo principalmente no sistema neurológico pode ser extremamente tóxico. Existe uma ligação entre exposição a adversidades precoces, levando ao estresse tóxico, e deficiências posteriores na aprendizagem, no comportamento e no bem-estar físico e mental (SHONKOFF; GARMER,

2012). Além disso, o tempo prolongado de hospitalização influencia de forma negativa o desenvolvimento motor (PANCERI *et al.*, 2012) e sociocomunicativo (CALDAS, 2017) de bebês internados por mais de trinta dias, o que coloca a população de recém-nascidos pré-termo sob considerável vulnerabilidade a alterações no desenvolvimento social, cognitivo e motor. As consequências desta exposição podem ser transitórias, mas podem também resultar em disfunções crônicas do desenvolvimento motor que não se restringem ao período neonatal (FORMIGA; LINHARES, 2009).

Nas últimas décadas os efeitos da prematuridade sobre o desenvolvimento motor foram relatados em diversos estudos, os quais apontaram a existência de diferenças significativas no desenvolvimento motor de recém-nascidos pré-termo e termo, com descrição de escores mais baixos nos resultados da avaliação motora de bebês pré-termo (FORMIGA *et al.*, 2015; FUENTARIA; SILVEIRA; PROCIANOY, 2017). Por exemplo, bebês pré-termo tiveram uma tendência a apresentar alterações no comportamento de habilidades manuais entre os 3 e 8 meses de idade corrigida, (HEATHCOCK; LOBO; GALLOWAY, 2008; SOARES-MARANGONI; VON HOFSTEN; TUDELLA, 2012), menor habilidade em tarefas manuais mais refinadas (SOARES-MARANGONI; CUNHA; TUDELLA, 2014), menor habilidade de sentar (FORMIGA; CEZAR; LINHARES, 2010), maior atraso motor nas posturas sentada e em pé aos 8 meses de idade corrigida (PIN et al., 2009). Na idade escolar, crianças nascidas pré-termo estão mais susceptíveis a prejuízos de desenvolvimento nas áreas motora, de comportamento e de desempenho escolar (MOREIRA; MAGALHÃES; ALVES, 2014), geralmente apresentam pior coordenação motora e maior índice de paralisia cerebral do que as nascidas a termo (ODD *et al.*, 2013). Essas crianças podem apresentar uma diminuição na excitabilidade corticomotora em áreas cerebrais associadas ao controle motor, mesmo não havendo lesão cerebral (PITCHER *et al.*, 2012).

Recém-nascidos pré-termo possuem também, maior vulnerabilidade à alterações sociocomunicativas (CALDAS *et al*, 2016; GUINCHAT *et al.*, 2012) quando comparados aos recém-nascidos a termo (DE SHUYMER *et al*, 2011; SHAH *et al.*, 2013). Alguns estudos relatam que recém-nascidos pré-termo podem exibir características comportamentais atípicas, tais como dificuldade em manter a atenção visual antes dos três meses, maior tempo para processar informações frente ao estímulo (SHAH *et al.*, 2013), menor exploração ativa de objetos, bem como diminuição nas atividades conjuntas com a mãe (DAWSON *et al.*, 2012).

Esses estudos demonstram que os fatores associados a prematuridade podem resultar em alterações do desenvolvimento motor em médio e longo prazos, reafirmando assim, a necessidade de medidas de avaliação, acompanhamento, estimulação e intervenção precoces

com ênfase na prevenção dessas alterações em recém-nascidos pré-termo (FORMIGA; PEDRAZZANI; TUDELLA, 2004).

A fim de minimizar os efeitos negativos da prematuridade no desenvolvimento infantil foram realizadas modificações na prática hospitalar com o objetivo de melhor adaptar o bebê à vida extrauterina. O Ministério da Saúde a partir da década de 1990 incorporou às políticas de saúde o Método Canguru como uma estratégia de humanização da assistência neonatal e no princípio de cidadania da família, maximizando assim, os fatores de proteção ao desenvolvimento do recém-nascido pré-termo (BRASIL, 2013; 2014). O Método Canguru inclui o cuidado humanizado, o contato pele a pele entre recém-nascido e seus pais, controle ambiental, redução da dor, cuidado com a família e suporte da equipe de saúde (BRASIL, 2016). Apesar dos esforços, as unidades hospitalares ainda submetem o recém-nascido ao risco de desconforto e dor dentro de sua rotina de cuidados assistenciais (REICHERT; LINS; COLLET, 2007), gerando *stress*, aumento do gasto energético, risco de hipóxia e dificuldades na amamentação (GRUNAU, 2002). Desta forma, são necessárias medidas terapêuticas interdisciplinares associadas à melhora do conforto do recém-nascido pré-termo (GASPARDO; LINHARES; MARTINEZ, 2005) para favorecer o sucesso da amamentação e o desenvolvimento motor e sociocomunicativo dessa população.

Uma das medidas terapêuticas, utilizadas dentro do Método Canguru, para favorecer o cuidado do recém-nascido nas unidades neonatais é a Posição Canguru, a qual promove uma experiência de contenção simulando o ambiente intrauterino do qual o recém-nascido foi privado. A posição canguru consiste em manter o recém-nascido na posição vertical, em contato pele a pele, junto ao peito dos pais, estando a criança somente de fraldas. Inicia-se o posicionamento canguru de forma precoce e crescente, por livre escolha da família, pelo tempo que ambos entenderem ser prazeroso e suficiente, mas, a cada vez que o bebê for para a posição recomenda-se que permaneça por, no mínimo, uma hora, para que possa receber seus benefícios. Deve ser realizada de maneira orientada, segura e acompanhada de suporte assistencial por uma equipe adequadamente treinada (BRASIL, 2013; 2016).

A posição canguru influencia positivamente a função cardiorrespiratória do recém-nascido. A posição vertical levemente inclinada durante a realização do posicionamento canguru favorece a mecânica respiratória, aumentando a eficiência do diafragma e a função pulmonar, com melhora no padrão respiratório e na redução da gemência (LUDINGTON-HOE *et al*. 1999), na saturação periférica de oxigênio (BERA *et al*., 2014; HUNT, 2008; LUDINGTON-HOE; FERREIRA; GOLDSTEIN, 1998; SOUKKA *et al*., 2014), na redução da frequência respiratória (BERA *et al*., 2014; FOHE; KROPF; AVENARIUS, 2000), e na

manutenção da frequência cardíaca dentro do intervalo de normalidade (BEGUM *et al*., 2008; BERA *et al*., 2014).

A maioria dos estudos têm apontado os benefícios da posição canguru no desenvolvimento motor e no comportamento de recém-nascidos pré-termo em curto, médio (BARRADAS *et al*., 2006; DAVID *et al*. 2012; DINIZ *et al*., 2013; MOTA; SÁ; FROTA, 2005; PADILHA; STEIDL; BRAZ, 2014) e longo prazos (FELDMAN; ROSENTHAL; EIDELMAN, 2014; SCHNEIDER *et al*., 2012) , nos movimentos generalizados do recém-nascido (REINAUX, 2005), no estabelecimento do aleitamento materno exclusivo (JAYARAMAN *et al*., 2017; SANTOS; AZEVEDO FILHO, 2016; PADILHA; STEIDL; BRAZ, 2014), na melhora da interação mãe-filho (NUNES et al., 2017), na melhora do ganho de peso ponderal e crescimento (EVEREKLIAN; POSMONTIER, 2017; SHARMA; MURKI; PRATAP, 2016) e na redução do estresse e da dor (MAIA; AZEVEDO; GONTIJO, 2011; RUSH, 2016; SANTOS; AZEVEDO FILHO, 2016;). Recém-nascidos pré-termo submetidos a posição canguru apresentam um aumento do tônus flexor fisiológico (DINIZ *et al*., 2013), assumindo uma postura de maior flexão de membros, associada a uma maior flexão do tronco quando avaliados pelo método Dubowitz (BARRADAS *et al*., 2006; GHISI; IWABE; TORELLO, 2004; MOTA; SÁ; FROTA, 2005). Alguns autores sugerem, ainda, que o posicionamento canguru pode apresentar um efeito positivo sobre o desenvolvimento motor dos recém-nascidos pré-termo quando avaliados por meio da escala Alberta Infant Motor Scale (DAVID *et al*., 2012). Por outro lado, outros estudos, ao investigarem os efeitos da posição canguru nos movimentos generalizados de recém-nascidos pré-termo, não encontraram diferenças significativas nesses movimentos antes e após o posicionamento (CONSTANTINOU *et al*., 1999). Outro estudo, utilizando o Test of Infant Motor Performance (TIMP), observou que os bebês submetidos ao mesmo posicionamento, inicialmente apresentaram um desempenho motor acima do esperado, mas que na idade de 44-48 semanas de idade corrigida apresentaram um atraso na movimentação de quadris e membros inferiores nos movimentos antigravitacionais e na posição em pé (REINAUX, 2005), sugerindo que outra intervenção poderia ser considerada em associação ao posicionamento canguru. Shaikh e Namrata (2017) compararam dois grupos de recém-nascidos pré-termo, um grupo recebeu massagem terapêutica associada a posição canguru e outro, recebeu somente a posição canguru. Os autores concluíram que o grupo que associou a massagem terapêutica com a posição canguru apresentou melhora mais significativa no estado fisiológico e comportamental quando comparado ao grupo que recebeu somente a posição canguru.

Em virtude dos diversos benefícios e por ser uma intervenção segura e de baixo custo, o método mãe-canguru pode ser implantado em qualquer hospital e proporciona à equipe multiprofissional um importante recurso adicional na assistência e atenção aos prematuros e suas famílias (BOUNDY *et al*, 2016; PADILHA; STEIDL; BRAZ, 2014). Ao projetar intervenções de cuidados de mãe canguru, fatores contextuais e normas socioculturais precisam ser levados em consideração (CHAN *et al*., 2016). O fisioterapeuta na unidade neonatal, como parte da equipe multiprofissional, deve visar às necessidades fisiológicas, respiratórias e motoras do recém-nascido pré-termo, estabelecendo uma adequada relação e interação do bebê com a família bem como estimulando e esclarecendo sobre a importância da posição canguru (AZEVEDO; CALIXTO; ABREU, 2017; BRADY; SMITH, 2015; SWEENEY *et al*., 2010).

Outra estratégia importante para melhorar o desenvolvimento do recém-nascido pré-termo são os programas de intervenção precoce, iniciados ainda no ambiente hospitalar, com o objetivo de aprimorar o desenvolvimento social, cognitivo e motor da criança. A intervenção precoce pode ser vista como um conjunto de ações de avaliação do neurodesenvolvimento e de terapias praticadas por meio de estimulação motora e sensorial com o objetivo de garantir um desenvolvimento adequado aos recém nascidos pré-termo promovendo uma transição eficiente do ambiente hospitalar para a comunidade e para o domicílio (GARCIA; GEPHART, 2013; OBERG *et al*., 2012). O recém-nascido pré-termo é incapaz de manter uma organização postural devido a sua hipotonia muscular e a dificuldade de auto-organização. Este fato, associado ao posicionamento no ambiente amplo da incubadora/berço e à ação da gravidade, facilita a fixação deste recém-nascido em uma postura extensora, favorecendo assim, o desenvolvimento de retrações musculares, podendo levar a atrasos no desenvolvimento motor (CARVALHO; SIQUEIRA, 2013) e na movimentação espontânea deste recém-nascido (CABRAL; SCHETTINO; POMPEU, 2015; EINSPIELER; PEHARZ; MARSCHIK, 2016). Busca-se, através dos manuseios da intervenção precoce, o favorecimento do ajuste maturacional da cadeia flexora (FERREIRA; SANTOS, 2016), a fim de estimular a organização corporal para favorecer a movimentação global espontânea (RAMACHANDRAN; DUTTA, 2013). A intervenção realizada nos primeiros meses de vida auxilia na modulação do tônus muscular, melhorando a postura, favorecendo as habilidades motoras (MARLOW, 2004) e, consequentemente, proporcionando experiências motoras adequadas.

Os programas de intervenção precoce para recém-nascidos pré-termo têm uma influência positiva nos resultados cognitivos e motores durante a infância, com benefícios cognitivos persistentes na idade pré-escolar (SPITTLE, 2015; VANDERVEEN *et al*., 2009). Uma revisão sistemática com meta-análise compilou evidências sobre a intervenção motora em

recém-nascidos pré-termo e sugeriu que as intervenções que continuam além do período de cuidados neonatais podem ter impacto no desenvolvimento motor de recém-nascidos prematuros, com os efeitos mais fortes observados antes dos 6 meses de idade, particularmente quando as intervenções direcionavam-se especificamente às habilidades motoras (HUGHES; REDSELL; GLAZEBROOK, 2016). Outra revisão vinculou os componentes da intervenção precoce (educação e apoio psicossocial aos pais e suporte ao desenvolvimento da criança) a aspectos psicossociais maternos e observou que as intervenções que incluíram apoio psicossocial resultaram em melhores resultados para as mães de bebês prematuros (BENZIES, 2013). A integração da família é necessária para que os resultados dos programas de intervenção precoce sejam bem sucedidos (FORMIGA; PEDRAZZANI; TUDELLA, 2010; OBERG *et al.*, 20
12; SWEENEY, 2010).

De forma geral, na intervenção precoce, almeja-se a interação do bebê com o ambiente por meio de estímulos sensório-motores, levando à obtenção de respostas próximas ao padrão de normalidade e à inibição da aprendizagem de posturas e movimentos inadequados (GONÇALVES, 2012). Estes estímulos devem ser elaborados com base na avaliação minuciosa do recém-nascido pré-termo e obedecer as fases neuroplásticas do mesmo, ou seja, a quantidade de estímulos deve estar estreitamente relacionada à capacidade, ao interesse e às possibilidades de cada recém-nascido (FORMIGA; PEDRAZZANI; TUDELLA, 2010).

Entre os instrumentos padronizados de avaliação do desenvolvimento motor e identificação de padrões motores atípicos, podemos destacar a Alberta Infant Motor Scale (AIMS), considerada um instrumento válido e confiável para avaliação do recém-nascido de risco nos primeiros 18 meses de vida (FUENTEFRIA; SILVEIRA; PROCIANOY, 2017; PIPER; DARRA, 1994). Além disso, o método de Prechtl para avaliação dos Movimentos Generalizados (GMA) permite uma avaliação qualitativa dos movimentos, é não invasivo e não intrusivo, utilizado como ferramenta diagnóstica para a detecção precoce de disfunção cerebral, considerado um avanço na predição do comprometimento neurodesenvolvimental (EINSPIELER; PRECHTL; 2005; EINSPIELER; PEHARZ; MARSCHIK, 2016; NOVAK *et al.*, 2017). A avaliação do desenvolvimento neuromotor faz parte do protocolo de seguimento de recém-nascidos egressos de unidades de terapia intensiva neonatais, sendo importante o uso de escalas e métodos confiáveis, de comprovada sensibilidade e especificidade para o correto direcionamento desta população para os programas de intervenção precoce.

Com base no contexto exposto e considerando que não há evidências suficientes que utilizem um protocolo de intervenção fisioterapêutica associado a posição canguru em

unidades neonatais, algumas questões despertam interesse: a) A combinação de um protocolo fisioterapêutico com a posição canguru é mais eficaz na melhoria dos desfechos clínicos, incluindo peso corporal, amamentação, estado comportamental e tempo de internação? b) A combinação de um protocolo fisioterapêutico com a posição canguru é mais eficaz na melhoria da postura e tônus muscular, e na normalização dos movimentos gerais, em recém-nascidos com repertório motor deficitário do que a Posição Canguru isolada?

Considerando as evidências de que a intervenção precoce (SYMINGTON; PINELLI, 2016) e a Posição Canguru (NUNES et al., 2017) melhoram a autorregulação e o vínculo mãe-bebê, hipotetiza-se que a aplicação do protocolo de intervenção fisioterapêutica associado à Posição Canguru por quinze dias em recém-nascidos pré-termo estáveis hospitalizados em uma unidade neonatal levará a uma melhoria nos desfechos clínicos, mantendo com segurança os sinais vitais. Como consequência, e com base nos efeitos benéficos da posição canguru (SANTOS; FILHO, 2016) e da intervenção precoce (MARLOW, 2004) sobre a postura corporal, tônus muscular e movimento espontâneo, espera-se que esses recém-nascidos apresentem melhores padrões motores imediatamente após o protocolo e com 12 semanas pós-termo, em comparação aos recém-nascidos do grupo controle.

2 Justificativa e Antecedentes

Este estudo investigará os diversos efeitos (fisiológicos e neurocomportamentais e motores) de um protocolo de intervenção fisioterapêutica associado ao posicionamento canguru em unidade neonatal no Hospital Universitário da Universidade Federal de Mato Grosso do Sul, com intuito específico de investigar o efeito do mesmo protocolo no estabelecimento e na manutenção da amamentação, no desconforto respiratório, no tônus muscular, na postura corporal e, especialmente, nas repercussões no desenvolvimento motor durante o período hospitalar e nos meses críticos após a alta hospitalar. Visto a ausência de evidências científicas correlacionando um protocolo de intervenção fisioterapêutica ao posicionamento canguru, utilizando método de ensaio clínico controlado randomizado, o presente projeto oferecerá fundamentação experimental para prática baseada em evidências por meio de um protocolo viável para as rotinas das equipes de unidades neonatais. Os resultados do presente projeto poderão fornecer suporte científico para implementação do protocolo de intervenção fisioterapêutica associado ao posicionamento canguru como procedimento de apoio à proteção do desenvolvimento infantil e cuidado humanizado do recém-nascido pré-termo.

O presente projeto buscará estabelecer um protocolo de intervenção precoce em nível hospitalar e consequentemente, estará contribuindo com a necessidade epidemiológica brasileira de maiores estudos voltados à estimulação precoce (BRASIL, 2016) em recém-nascidos hospitalizados com risco ou alterações neurológicas estabelecidas.

3 Objetivos

3.1 Objetivo geral

Investigar efeitos em curto e médio prazos da aplicação de um protocolo de intervenção fisioterapêutica associado ao posicionamento canguru ao longo de dez dias sobre o comportamento neuromotor e desfechos clínicos de recém-nascidos pré-termo estáveis internados em unidade neonatal.

3.2 Objetivos específicos

- Verificar os movimentos generalizados de recém-nascidos pré-termo que receberam o protocolo de intervenção fisioterapêutica associado ao posicionamento canguru e em recém-nascidos pré-termo controle antes e após o tempo de protocolo, bem como às 12 semanas pós-termo.
- Verificar o ganho de peso corporal em recém-nascidos pré-termo que receberam o protocolo de intervenção fisioterapêutica associado ao posicionamento canguru e em recém-nascidos pré-termo controle.
- Verificar a postura e tônus em recém-nascidos pré-termo que receberam o protocolo de intervenção fisioterapêutica associado ao posicionamento canguru e em recém-nascidos pré-termo controle.
- Verificar o estado comportamental em recém-nascidos pré-termo que receberam o protocolo de intervenção fisioterapêutica associado ao posicionamento canguru e em recém-nascidos pré-termo controle.
- Verificar o tempo de sucesso no estabelecimento e manutenção do aleitamento materno em recém-nascidos pré-termo que receberam o protocolo de intervenção fisioterapêutica associado ao posicionamento canguru e em recém-nascidos pré-termo controle.
- Verificar o tempo de hospitalização em recém-nascidos pré-termo que receberam o protocolo de intervenção fisioterapêutica associado ao posicionamento canguru e em recém-nascidos pré-termo controle.

- Verificar os escores de desconforto respiratório em recém-nascidos pré-termo que receberam o protocolo de intervenção fisioterapêutica associado ao posicionamento canguru e em recém-nascidos pré-termo controle.
- Verificar os parâmetros fisiológicos (sinais vitais) em recém-nascidos pré-termo que receberam o protocolo de intervenção fisioterapêutica associado ao posicionamento canguru e em recém-nascidos pré-termo controle.
- Comparar os efeitos entre recém-nascidos pré-termo que receberam o protocolo de intervenção fisioterapêutica associado ao posicionamento canguru e recém-nascidos pré-termo controle.

4 Metodologia

4.1 Desenho

Este projeto caracteriza um ensaio clínico/terapêutico controlado, randomizado, longitudinal, de grupos paralelos.

4.2 Participantes

Participarão deste estudo 34 recém-nascidos pré-termo, nascidos com idade gestacional igual ou inferior a 34 semanas, no período de 34 a 36 semanas e 6 dias de idade pós-menstrual, hospitalizados em Unidade de Cuidados Intermediários Neonatal (UCIN). Para estimar o número mínimo de participantes, considerando uma diferença de pelo menos 50% entre os grupos quanto à qualidade dos GMs após a intervenção utilizando o teste qui-quadrado, o número sugerido de amostras é de 13 participantes por grupo (poder de 80%; $\alpha = 5\%$). Considerando possíveis perdas durante o acompanhamento, incluiremos dois grupos de 17 participantes. Os recém-nascidos serão alocados aleatoriamente em dois grupos: *grupo experimental* (n=17), que receberá o protocolo de intervenção fisioterapêutica associado a posição canguru; e *grupo controle* (n=17), que receberá somente posição canguru.

4.3 Critérios de inclusão

Serão incluídos recém-nascidos pré-termo com idade gestacional ao nascer igual ou menor do que 34 semanas, com mais de 72 horas de vida pós-natal, internados em UCIN, com quadro clínico estável, com movimentos generalizados de característica pobre repertório, sem

necessidade de ventilação mecânica invasiva ou não invasiva, podendo apresentar necessidade de suporte de oxigênio por cateter nasal, residentes em Campo Grande-MS.

Os prontuários médicos, de enfermagem, de fisioterapia e a indicação dos neonatologistas e da equipe multiprofissional serão considerados como referência para a seleção ou não-inclusão dos pacientes no estudo segundo os critérios anteriormente descritos.

4.4 Critérios de exclusão

Serão excluídos do estudo os recém-nascidos pré-termo com idade gestacional ao nascer igual ou maior do que 34 semanas e 1 dia e que, apresentem pelo menos uma das seguintes condições: malformações congênitas, síndromes cromossômicas, infecções em tratamento (alteração de hemograma e hemocultura positiva), alterações neurológicas (hemorragia intracraniana grau III ou IV e malformações cerebrais), anóxia neonatal (Apgar menor que 7 no quinto minuto) e infecções congênitas. Além disso, serão excluídos os pacientes com outras contra-indicações à realização do protocolo de intervenção fisioterapêutica e posição canguru, bem como aqueles cujos pais e/ou responsáveis não concordarem com a participação do recém-nascido no estudo ou que solicitem a retirada do recém-nascido do estudo.

Serão excluídos também do estudo recém-nascidos que apresentarem reações adversas ao protocolo fisioterapêutico e ao posicionamento canguru, como instabilidade clínica durante o procedimento (cianose, choro, letargia, fácies de dor, hiperemia generalizada, entre outros) ou em curto e médio prazos (piora do quadro clínico, intolerância à alimentação ou distúrbios comportamentais). Também poderão ser excluídos aqueles que apresentarem intercorrências que impossibilitem a aplicação do protocolo, como necessidade de suporte ventilatório mecânico invasivo ou não invasivo ou suporte de oxigenoterapia por meio de Caixa de Hood, além de procedimentos cirúrgicos.

4.5 Locais de recrutamento e coleta de dados

Os recém-nascidos serão recrutados nas Unidades de Cuidados Intermediários Neonatal (UCIN) do Hospital Universitário Maria Aparecida Pedrossian (HUMAP), da Universidade Federal de Mato Grosso do Sul (UFMS) e nas UCIN do Hospital Regional de Mato Grosso do Sul (HRMS). Durante o período de internação a coleta de dados ocorrerá na própria UCIN e, após a alta, no Laboratório de Estudos em Neuropediatria (LABEN) na Clínica Escola Integrada da UFMS ou ambiente domiciliar.

4.6 Equipamentos e materiais

Serão utilizados pulso-oxímetro (Dixtal Biomédica®) para aferir a frequência cardíaca e os níveis periféricos de oxigênio, bem como termômetro digital periférico para aferir a temperatura corporal, antes da intervenção. Também será utilizada uma camisola para uso materno e uma faixa de algodão moldável para contenção do recém-nascido junto à mãe. Estes materiais serão os de uso de rotina na UCIN

Os seguintes instrumentos ou escalas de avaliação serão utilizados: Boletim de Silverman-Andersen (BSA), Avaliação Qualitativa dos Movimentos Generalizados de Prechtl (GMA), Escala Adaptada de Brazelton, e Triagem Neuromotora Neonatal (TNN).

Duas câmeras filmadoras digitais (Sony®), acopladas a tripés, serão utilizadas para filmar as avaliações, sendo uma para o ambiente hospitalar e outra para o ambiente da clínica. Os vídeos serão transferidos para um computador *notebook* e armazenados em disco rígido externo (HD).

4.7 Instrumentos de avaliação

4.7.1 Avaliação qualitativa dos movimentos generalizados de Prechtl (GMA)

Os movimentos generalizados (GMs) compõem o principal repertório motor espontâneo nos primeiros meses de vida do bebê, estando presentes desde nove semanas de idade gestacional até aproximadamente vinte semanas de vida pós-natal. Abrangem movimentos fluentes e elegantes com sequência variável, do pescoço, tronco, extremidades superiores e extremidades inferiores (PRECHTL, 1990; EINSPIELER; PRECHTL, 2005; EINSPIELER; PEHARZ; MARSCHIK, 2016). A presença, a qualidade e a intensidade destes movimentos fornece informações sobre a integridade funcional do sistema nervoso central do recém-nascido (HADDERS-ALGRA, 2014), sugerindo ser uma das ferramentas mais preditivas para detectar precocemente a disfunção cerebral antes da idade corrigida de cinco meses (EINSPIELER; PEHARZ; MARSCHIK, 2016; EINSPIELER; PRECHTL, 2005; EINSPIELER *et al*., 1997; NOVAK *et al*., 2017). A avaliação dos GMs utilizando o método de Prechtl classifica os GMs como: a) *Writhing Movements*, podendo ser "normais", "pobre repertório", "*cramped-synchonized*" ou "caóticos"; e b) *Fidgety Movements*, podendo ser normais, anormais ou ausentes (EINSPIELER; PRECHTL, 2005; EINSPIELER *et al*., 1997). Não é permitida a manipulação do bebê durante a avaliação, sendo os recém-nascidos posicionados em supino, usando somente fraldas ou roupas curtas que ofereçam liberdade de

movimentos (EINSPIELER; PRECHTL, 2005). A avaliação dura cerca de 5 minutos e deve ser filmada para análise.

4.7.2 Triagem Neuromotora Neonatal - TNN

A Triagem Neuromotora Neonatal (TNN) foi desenvolvida por Gonçalves (2012), com base no protocolo da Escala dos Sinais Neurológicos de Dubowitz (1970), sendo utilizada como instrumento de avaliação para diagnóstico precoce de distúrbios neuromotores de recém-nascidos termo e pré-termo (BIAZUS *et al*., 2016; GONÇALVES, 2010). A TNN é composta por 18 itens e avalia: postura, tônus passivo, tônus ativo, reflexos primitivos e reações corporais de reajuste automático. A aplicação do protocolo deve ser de preferência com o bebê em estado de alerta, iniciando-se pela observação da postura, seguida da avaliação do tônus passivo dos membros superiores, membros inferiores, avaliação do tônus ativo, reflexos primitivos e reações de alinhamento. A pontuação final é composta pela soma dos escores obtidos em cada um dos itens dos sinais neurológicos avaliados e classificado em hipotonia, normotonia e hipertonia. A conclusão diagnóstica é obtida através da soma desses escores associada aos dados clínicos do bebê. É de fácil aplicação e dura, em média, 10 minutos, estando o bebê no berço ou mesa de exame (GONÇALVES, 2012) (Figura 1).

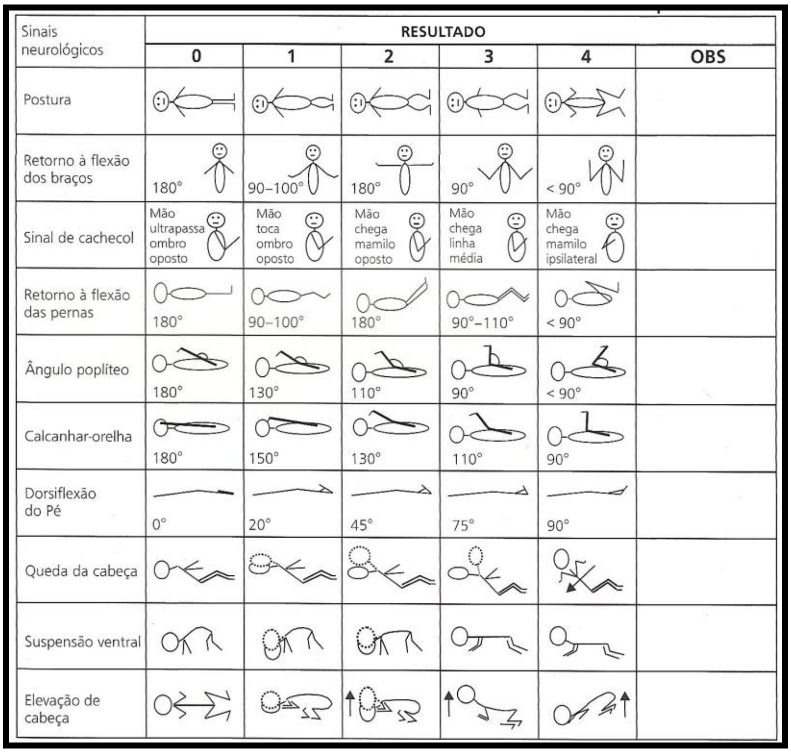

Sinais neurológicos	RESULTADO					
	0	1	2	3	4	OBS
Postura						
Retorno à flexão dos braços	180°	90–100°	180°	90°	< 90°	
Sinal de cachecol	Mão ultrapassa ombro oposto	Mão toca ombro oposto	Mão chega mamilo oposto	Mão chega linha média	Mão chega mamilo ipsilateral	
Retorno à flexão das pernas	180°	90–100°	180°	90°–110°	< 90°	
Ângulo poplíteo	180°	130°	110°	90°	< 90°	
Calcanhar-orelha	180°	150°	130°	110°	90°	
Dorsiflexão do Pé	0°	20°	45°	75°	90°	
Queda da cabeça						
Suspensão ventral						
Elevação de cabeça						

Figura 1. Ilustração dos sinais neurológicos da Triagem Neuromotora Neonatal. Fonte: Gonçalves (2012).

4.7.3 Escala Adaptada Neonatal de Brazelton

A Escala Adaptada de Avaliação do Comportamento Neonatal de Brazelton foi utilizada para avaliar o estado comportamental. A escala classifica o comportamento do recém-nascido com base em seu estado atual, variando de 1 a 6. Estado 1: sono profundo, imóvel, com respiração regular; Estado 2: sono leve ou ativo, olhos fechados com movimentos corporais ocasionais; Estado 3: sonolência, abrindo e fechando os olhos intermitentemente; Estado 4: alerta tranquilo, com mínima atividade física; Estado 5: completamente desperto, com movimentos vigorosos (alerta ativo); Estado 6: choro (BRAZELTON et al., 1976).

4.7.4 Boletim de Silverman-Andersen - BSA

Para quantificar o grau de desconforto respiratório e acompanhar a sua evolução será utilizado o Boletim de Silverman-Andersen – BSA sendo este, um método clínico útil para estimar a gravidade do comprometimento pulmonar. Este boletim gera um escore correspondente a soma das notas de 0 a 2 atribuídas aos parâmetros: retração intercostal, retração xifóide, batimento de asa nasal, e gemido expiratório. Uma somatória das notas de todos os parâmetros inferior a 5 aponta dificuldade respiratória leve; quando igual a 10, indica grau máximo de dispnéia pulmonar (SILVERMAN; ANDERSEN, 1956; BRASIL, 2011b) (Figura 2).

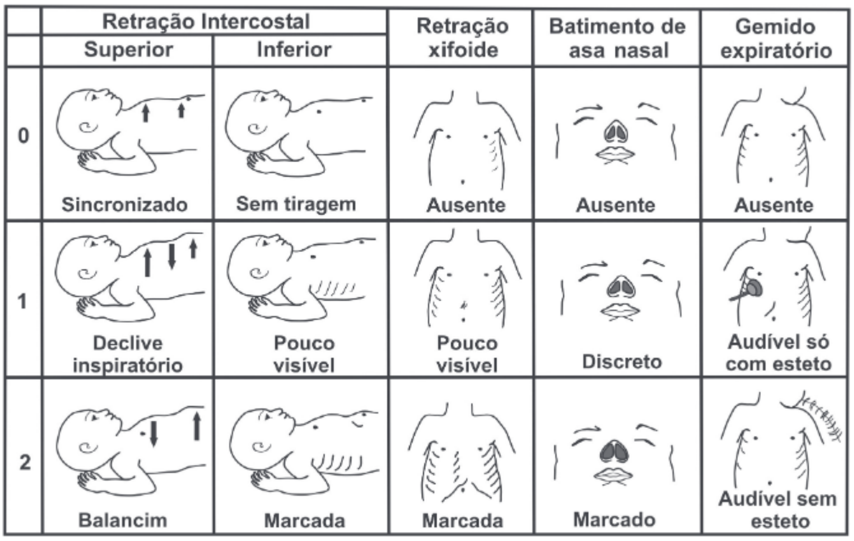

Figura 2. Ilustração do Boletim de Silverman – Andersen. Fonte: Brasil (2011b)

4.8 Procedimentos

4.8.1 Procedimentos de ética e registro do estudo

O estudo será encaminhado ao Comitê de Ética da Universidade Federal de Mato Grosso do Sul, obedecendo as Diretrizes e Normas Regulamentadoras das Pesquisas Envolvendo Seres Humanos (Res. 466/2012), do Conselho Nacional de Saúde). Antes do início do recrutamento dos sujeitos o projeto será registrado no Registro Brasileiro de Ensaios Clínicos.

4.8.2 Procedimentos de randomização e alocação

A distribuição dos recém-nascidos nos grupos será aleatória, seguindo randomização computacional prévia em blocos de 4 bebês para cada grupo. A alocação para os grupos será ocultada dos pesquisadores envolvidos através de envelopes opacos individuais, selados e numerados sequencialmente. Os envelopes serão abertos antes da realização do protocolo experimental ou controle, de acordo com a inclusão dos bebês no estudo. As avaliações serão filmadas e pontuadas posteriormente, sem identificação da alocação do recém-nascido nos grupos, o que possibilitará avaliação posterior em condição cega.

4.8.3 Procedimentos de recrutamento dos participantes

Primeiramente, serão recrutados os recém-nascidos da UCIN que satisfaçam aos critérios de elegibilidade do estudo. Logo após, a mãe ou o responsável serão abordados e convidados a participar, sendo explicados os objetivos e procedimentos que serão realizados no estudo. Se houver concordância, a participação do recém-nascido deverá ser autorizada pela mãe ou responsável por meio de assinatura do Termo de Consentimento Livre e Esclarecido.

4.8.4 Procedimentos de coleta de dados e teste

Os dados de história clínica do recém-nascido serão primeiramente anotados em ficha de anamnese, obtendo-se dados referentes aos critérios de elegibilidade dos recém-nascidos. Os dados do recém-nascido e das mães serão obtidos a partir dos prontuários médicos, de enfermagem, da fisioterapia e da Caderneta da Criança.

Será registrado o número de dias desde o início do protocolo até o dia do estabelecimento do aleitamento materno, bem como o período de sua manutenção, em dias, até a alta hospitalar. Também será verificada a manutenção do aleitamento materno exclusivo após

a alta, na idade de 12 semanas pós-termo. Para esses registros, a mãe e a equipe multiprofissional serão consultadas. Também será registrado o peso corporal diário dos recém-nascidos até o término do protocolo, bem como o tempo total de hospitalização.

4.8.4.1. Avaliações na UCIN

Os recém-nascidos pré-termo de ambos os grupos experimental e controle serão avaliados pelo pesquisador na UCIN quanto às variáveis fisiológicas (frequência cardíaca, frequência respiratória, saturação periférica de oxigênio, temperatura corporal) e estado comportamental em 3 momentos diariamente: I) imediatamente antes do protocolo (experimental ou controle), com o bebê ainda na incubadora/berço; II) 60 minutos após o início da posição canguru (experimental ou controle), enquanto o bebê permanece na posição; e III) aos 30 minutos seguintes com o bebê posicionado na incubadora/berço.

Os GMs e a triagem neuromotora (postura e tônus) serão avaliados em 2 momentos: I) um dia anterior ao início do protocolo; II) um dia depois do término do protocolo.

4.8.4.2. Avaliações após a alta hospitalar

Após a alta hospitalar, os GMs dos bebês de ambos os grupos será com 12 semanas pós-termo, por meio do GMA. Essas avaliações serão realizadas entre as alimentações (após 1 hora a 1 hora e 30 minutos) e não deverão coincidir com dias de vacinação. Os bebês deverão estar no estado de alerta ativo, ou seja, estado 4 (com olhos abertos, sem choro, mas exibindo movimentos grosseiros) (BRAZELTON et al., 1976). As avaliações serão realizadas sobre um colchonete, permanecendo o bebê preferencialmente apenas com fralda. Caso o bebê não esteja colaborativo, apresentando choro ou inquietação, a avaliação será interrompida, o bebê acalmado e a avaliação reiniciada. Permanecendo o bebê inquieto, será marcada outra data.

As avaliações serão filmadas e pontuadas posteriormente. A câmera será instalada em um tripé com distância e ângulo suficientes para visualizar a movimentação do bebê em corpo inteiro. As avaliações após a alta hospitalar poderão ser realizadas por meio de visita domiciliar ou no Laboratório de Estudos em Neuropediatria (LABEN) na Clínica Escola Integrada-UFMS, a critério dos pais/responsáveis. Serão realizados treinamento e índice de concordância entre pelo menos dois pesquisadores/observadores experientes para avaliação com os instrumentos, desejando-se o mínimo de 80% de índice.

4.8.5 Protocolo experimental

O protocolo experimental será composto de um protocolo de intervenção fisioterapêutica associado a posição canguru.

4.8.5.1 Protocolo de intervenção fisioterapêutica

O protocolo de intervenção fisioterapêutica será aplicado apenas no grupo experimental. A intervenção consistirá dos seguintes manuseios: I) Pompagem lombossacral; II) Alongamento posterior; III) Alongamento da musculatura cervical; IV) Dissociação tóraco-umeral; V) Exercício terapêutico de sentir a cabeça com as mãos e, VI) Posicionamento em decúbito dorsal permitindo movimentos espontâneos do recém-nascido pré-termo. Inicia-se o protocolo pelo manuseio da pompagem lombossacral com o recém-nascido em decúbito dorsal e cabeça ligeiramente flexionada, terapeuta com a mão direita posicionada na região lombossacra e a esquerda dando apoio na região anterior da pelve, realiza delicadamente com os dedos anular e médio da mão direita uma tração no sentido caudal (GONÇALVES, 2012; BUSQUET-VANDERHEYDEN, 2009); sem perder o posicionamento pélvico em retroversão muda-se o recém-nascido para decúbito lateral e faz-se o manuseio de alongamento posterior mantendo a mão direita na região sacral e mão esquerda posicionada sob a escama occipital realizando uma sutil e passiva tração, sempre na expiração, em ambos os lados do eixo vertebral (LIMA, 2012; BUSQUET-VANDERHEYDEN, 2009); logo após este manuseio realiza-se o alongamento da região cervical com a mão direita do terapeuta envolvendo o ombro e a mão esquerda apoiando a região occipital e temporal mantendo o recém-nascido elevado a aproximadamente a 30º, deslizar a cabeça e o pescoço para a lateral direita, enquanto o ombro esquerdo é suavemente rebaixado, soltar o ombro e retornar a cabeça para linha média, inverter as mãos e realizar o movimento para o outro lado (DUARTE *et al*., 2011); imediatamente após este manuseio será realizado a dissociação tóraco-umeral, onde o terapeuta envolve com uma das mãos a região do ombro e realiza movimentos circulares em direção póstero-anterior (LIMA, 2012); no exercício de sentir a cabeça com as mãos o recém-nascido permanece em decúbito dorsal, a coluna cervical deve ser retificada, tronco e membros inferiores flexionados, leva-se as mãos do recém-nascido até a região parietal deslizando as palmas das mãos anteriormente e lateralmente sobre a face esfregando-as à frente (DUARTE *et al*., 2011) e, para finalizar, o recém-nascido será posicionado em decúbito dorsal na incubadora/berço para realizar movimentos espontâneos com o ninho um pouco afastado. O protocolo terá duração de 15 minutos e logo após o recém-nascido será colocado em posição canguru com sua mãe por 60 minutos.

4.8.5.2 Protocolo posição canguru

Na posição canguru o recém-nascido deverá ser colocado somente de fraldas em posição vertical ou diagonal elevada, entre as mamas da mãe, com a cabeça lateralizada, membros superiores e inferiores em flexão e adução. A mãe deverá estar sem sutiã e vestida com uma camisola com abertura anterior. Após o posicionamento do bebê a mãe deve envolvê-lo com a camisola e logo após deve-se envolver a díade com uma faixa de algodão moldável para maior segurança (BRASIL, 2013).

O protocolo experimental terá início quando o bebê completar 34 semanas de idade pós-menstrual, mais de 72 horas de vida, peso acima de 1100g, estável clinicamente, respeitando-se os sinais de estresse (DUARTE *et al*., 2011) e será conduzido durante os 15 dias seguintes. Logo, ao final do protocolo, cada recém-nascido terá recebido 15 sessões do protocolo fisioterapêutico associado a posição canguru. As intervenções serão programadas para o intervalo entre os horários de alimentação (via oral ou sonda).

Serão minimizados fatores de estresse como ruídos e luminosidade durante a realização do protocolo experimental e controle. O procedimento será realizado somente após o recém-nascido ter sido alimentado, aguardando pelo menos 60 minutos após a mamada.

4.8.3 Protocolo controle

Os recém-nascidos do grupo controle serão submetidos aos mesmos procedimentos do posicionamento canguru descritos anteriormente e não receberão o protocolo de intervenção fisioterapêutica. Desta forma, o recém-nascido estará posicionado na incubadora/berço antes de ser posicionado em canguru com a mãe.

4.9 Desfechos

4.9.1 Desfechos primários

- Ganho de Peso: registro do peso corporal diário como parâmetro de acompanhamento ponderal do recém-nascido pré-termo durante a aplicação do protocolo (experimental e controle).
- Comportamento Neuromotor verificado pelas classificações alcançadas nos GMs segundo a GMA (EINSPIELER *et al*., 1997).

4.9.2 Desfechos secundários

- Postura e Tônus muscular: comportamento quanto a postura e tônus muscular, verificados pela classificação alcançada seguindo o método de avaliação dos sinais neurológicos da triagem neuromotora neonatal (GONÇALVES, 2012).
- Estado Comportamental: estado comportamental segundo a escala adaptada de Brazelton (BRAZELTON et al., 1976).
- Tempo para Estabelecimento do Aleitamento Materno: definido como o intervalo de tempo, em dias, entre o início do protocolo e o sucesso da amamentação no seio. O sucesso da amamentação será considerado conforme os principais pontos-chaves preconizados para posicionamento e pega adequados durante a mamada: face do bebê de frente para a mama, com nariz na altura do mamilo, corpo do bebê próximo ao da mãe, bebê com cabeça e tronco alinhados; bebê bem apoiado; mais aréola visível acima da boca do bebê; boca bem aberta; lábio inferior virado para fora; e queixo tocando a mama (BRASIL, 2011a).
- Manutenção do Aleitamento Materno Exclusivo: definido como a permanência do aleitamento materno exclusivo na idade corrigida de 2 semanas pós-termo, 12 semanas pós-termo e aos 6 meses, sendo dicotômica.
- Tempo de Internação Hospitalar: definido como o tempo médio em dias de permanência do recém-nascido dentro do ambiente hospitalar.
- Grau de Desconforto Respiratório: obtido pela soma da pontuação segundo o BSA (SILVERMAN; ANDERSEN, 1956; BRASIL, 2011b) em cada dia de protocolo.
- Parâmetros Fisiológicos: sinais vitais (frequência cardíaca, frequência respiratória, saturação periférica de oxigênio, temperatura corporal) avaliados e registrados antes e após a realização do protocolo (experimental e controle), em cada dia.

4.10 Análise de dados

Para a análise estatística dos dados será utilizado suporte do pacote estatístico SPSS 23.0. Para análise das variáveis dependentes será aplicado o Teste de Levene de Homogeneidade de Variância e o teste Shapiro-Wilk de Normalidade. Uma vez que as suposições sejam atendidas, poderão ser aplicados testes paramétricos e não-paramétricos dependendo das condições de homogeneidade e normalidade da amostra. Primeiramente, será realizada estatística descritiva para caracterizar a amostra. Os efeitos de tratamento serão obtidos pelas comparações entre os grupos.

Para testar as diferenças entre os grupos (efeitos do tratamento) para variáveis categóricas (qualidade dos GMs, estado comportamental, sucesso e manutenção da amamentação), será aplicado o teste qui-quadrado. Para testar as diferenças entre os grupos em relação ao peso corporal, será utilizado o teste t independente. Para outras variáveis contínuas, serão utilizados testes t ou o teste de Mann–Whitney para calcular as diferenças entre os grupos. Médias ajustadas e intervalos de confiança de 95% também podem ser calculados utilizando o modelo linear misto (GLM) para as variáveis contínuas, considerando os termos de interação grupo x dias de avaliação.

Para cálculo do tamanho do efeito de intervenção será adotado intervalo de confiança de 95% e serão considerados o d de Cohen ou r (r = escore z / √amostra total), a depender da utilização de testes paramétricos ou não paramétricos, respectivamente. Será considerado o nível de significância α=5% para todas as análises.

5. Riscos e Benefícios

O presente estudo não apresenta riscos físicos ou psíquicos para o recém-nascido, mas podem existir sinais de sobrecarga sensorial tais como: flutuações da cor (palidez, cianose perioral, dentre outras), alterações cardiorrespiratórias (bradicardia, respiração irregular, apnéia, frequência respiratória irregular), alterações de estado (soluços, bocejos, salivação, sustos, choro, irritabilidade) e sinais de retraimento no recém-nascido ao ser manuseado. Ao detectar sinais de sobrecarga, deve-se interromper o manuseio para permitir a organização do recém-nascido, através de manobras como posicionamento e toque firme. Quanto aos benefícios, a pesquisa proporcionará uma estimulação/intervenção precoce aos recém-nascidos pré-termo hospitalizados, potencializando a interação do bebê com o ambiente por meio de estímulos sensório-motores e que o mesmo interprete estes estímulos como sendo prazeroso (sinais de aproximação). Já a posição canguru é uma intervenção segura e de baixo custo, podendo ser realizada em qualquer hospital e proporciona à equipe multiprofissional um importante recurso adicional na assistência e atenção aos recém-nascidos pré-termo e suas famílias, tornando-se essas, parte ativa no tratamento, promovendo uma transição eficiente do ambiente hospitalar para o domiciliar.

6. Orçamento

Foram orçados para o estudo gastos com equipamento, material de consumo e impressões, conforme quadro a seguir:

Materiais e Equipamentos	Quantidade	Valor Unitário	Valor Total
Câmera filmadora	02	R$1500,00	R$3000,00
Tripé com cabeçote	02	R$700,00	R$1.400,00
Notebook	01	R$3.500,00	R$3.500,00
HD externo (1TB)	01	R$440,00	R$440,00
Brinquedos de bebê	07	R$15,00	R$105,00
Colchonete de espuma	02	R$80,00	R$160,00
Oxímetro	01	R$ 2.000,00	R$ 2.000,00
Termômetro	01	R$ 15,00	R$ 15,00
Impressões	2000	R$ 0,20	R$ 400,00
**TOTAL**	-	-	**R$11.020,00**

Os equipamentos e materiais já foram adquiridos e fazem parte do Laboratório de Estudos em Neuropediatria (LABEN). No entanto, sendo necessário, os custos serão arcados com recursos pessoais do pesquisador. O projeto será também submetido a Editais e Chamadas junto a agências de fomento.

7. Cronograma

Identificação da Etapa	Início	Término
Submissão do projeto ao Comitê de Ética e Pesquisa da Universidade Federal de Mato Grosso do Sul	27/09/2018	27/09/2018
Atualização bibliográfica	27/09/2018	31/07/2021
Submissão ao REBEC (Registro Brasileiro de Ensaios Clínicos)	27/11/2018	30/11/2018
Início do recrutamento e coleta de dados	01/18/2018	30/06/2020
Redação de artigos	01/08/2019	01/08/2021
Divulgação em eventos científicos	01/09/2019	31/08/2021
Tabulação dos dados	30/06/2020	30/07/2020
Tratamento estatístico e Discussão dos resultados	30/07/2020	30/10/2020
Pré defesa/ Qualificação	01/11/2020	30/11/2020
Discussão dos resultados	30/10/2020	30/06/2021
Apresentação e defesa pública	01/09/2021	30/09/2021
Entrega da versão final	01/11/2021	30/11/2021

O cronograma poderá sofrer alterações por motivos de força maior.

4. Relevância e Viabilidade Econômica e de Execução do Trabalho

Há uma grande aceitação e aplicação da intervenção precoce e da posição canguru em unidades neonatais brasileiras, devido aos efeitos fisiológicos benéficos observados na rotina clínica. Porém, existe uma escassez de estudos investigando a associação de um protocolo de intervenção fisioterapêutica, composto por manuseios e exercício terapêutico, associado ao posicionamento canguru. Neste sentido, o presente estudo contribuirá com suporte científico para prática baseada em evidências nesse tema. Seus resultados poderão ser utilizados para implementar o protocolo fisioterapêutico associado a posição canguru na unidade neonatal do Hospital Universitário Maria Aparecida Pedrossian (HUMAP) – UFMS e no Hospital Regional de Mato Grosso do Sul (HRMS), podendo servir de modelo para outros hospitais brasileiros e do mundo, tendo em vista publicações e divulgações internacionais. Além disso, como este projeto será executado durante um Programa de Doutorado, terá impacto na formação de recursos humanos junto ao HUMAP - UFMS.

Ressalta-se que, levando em consideração a abordagem atual de humanização do cuidado neonatal, bem como a recente necessidade epidemiológica brasileira de maiores estudos voltados à estimulação precoce em recém-nascidos hospitalizados com risco ou alterações neurológicas estabelecidas, torna-se claro a necessidade de investigações terapêuticas que colaborem para prevenir e minimizar morbidades, impactando de forma favorável a economia do sistema de saúde.

No que se diz respeito a viabilidade financeira para realizar o projeto, os materiais e equipamentos necessários para a pesquisa dentro do hospital, tais como: oxímetro e termomêtro já foram adquiridos e são utilizados individualmente na rotina de assistência ao recém-nascido da Unidade Neonatal do HUMAP-UFMS e do HRMS, devendo os mesmos serem utilizados para a pesquisa. As câmeras filmadoras, tripés, colchonetes, notebook, HD externo e brinquedos já foram adquiridos, em momento anterior, e pertencem ao Laboratório de Estudos em Neuropediatria (LABEN), coordenado pela Profª. Dra. Daniele de Almeida Soares- Marangoni, sendo portanto viável do ponto de vista financeiro a realização da pesquisa.

Referências

ADAMS-CHAPMAN, I. Neurodevelopmental outcome of the late preterm infant. **Clinics in Perinatology**, v. 33, n. 4, p. 947-964, Dec. 2006.

ALBERTINE, K. H. Brain injury in chronically ventilated preterm neonates: collateral damage related to ventilation strategy. **Clinics in Perinatology**, v. 39, n. 3, p.727-40, Sept. 2012.

AMIN, S. B.; MERLE, K. S.; ORLANDO, M. S.; DALZELL, L. E.; GUILLET, R. Brainstem maturation in premature infants as a function of enteral feeding type. **Pediatrics**, v. 106, n. 2, p. 318-322, Sept. 2000.

ANAND, K. J. Effects of perinatal pain and stress. **Progress in Brain Research**, v. 122, p. 117-129, Feb. 2000.

AZEVEDO, V. M. G. O.; CALIXTO, A. F.; ABREU, L. R. Efeitos da posição canguru no sistema cardiorrespiratório de recém-nascidos pré-termo. *In*: ASSOCIAÇÃO BRASILEIRA DE FISIOTERAPIA CARDIORRESPIRATÓRIA E FISIOTERAPIA EM TERAPIA INTENSIVA; MARTINS, J. A., ANDRADE, L. B., RIBEIRO, S. N. S. (Org.). **PROFISIO Programa de atualização em fisioterapia pediátrica e neonatal:** cardiorrespiratória e terapia intensiva. Ciclo 6. Porto Alegre: Artmed Panamericana, 2017. v. 3, p.119-149.

BARTOCCI, M.; BERGQVIST, L. L.; LAGERCRANTZ, H.; ANAND, K. J. Pain activates cortical areas in the preterm newborn brain. **Pain**, v. 122, n. 1-2, p. 109-117, May 2006.

BEGUM, E. A.; BONNO, M.; OHTANI, N.; YAMASHITA, S.; TANAKA, S.; YANAMOTO, H.; KAWAI, M.; KOMADA, Y. Cerebral oxygenation responses during kangaroo care in low birth weight infants. **BioMedCentral Pediatrics**, v. 51, n. 8, Nov. 2008.

BENZIES, K.M.; MAGILL-EVANS, J.E.; HAYDEN, K. A.; BALLANTYNE, M. Keu components of early intervention programs for preterm infants and their parents: a systematic review and meta-analysis. **BioMedCentral Pregnancy and Childbirth,** v. 13, suppl. 1, n. 10, Jan. 2013.

BERA, A.; GHOSH, J.; SINGH, A. K.; HAZRA, A.; SOM, T.; MUNIAN, D. Effect of kangaroo mother care on vital physiological parameters of the low birth weight infants. **Indian Journal of Community Medicine :** official publication of indian association of preventive & social medicine, v. 39, n. 4, p. 245-9, Oct./Dec. 2014.

BIAZUS, G. F.; KUPKE, C. C.; MATOS, S. S.; JANDT, S. R. Avaliação fisioterapêutica em neonatos que apresentaram asfixia perinatal e que foram submetidos à hipotermia terapêutica. **Revista Fisioterapia Saúde Funcional**, v. 5, n. 1, p. 59-68, Jan./Jul. 2016.

BLENCOWE, H.; COUSENS, S.; CHOU, D.; OESTERGAARD, M.; SAY, L.; MOLLER, A.; KINNEY, M. Born Too Soon: The global epidemiology of 15 million preterm births. **Reproductive Health**, v. 10, supl. 1, S2, p. 1-14, Nov. 2013.

BOUNDY, E. O., DASTJERDI, R.; SPIEGELMAN, D.; FAWZI, W. W.; MISSMER, S.A.; LIEBERMAN, E.; KAJEEPETA, S.; WALL, S.; CHAN, G. J. Kangaroo mother care and neonatal outcomes: a meta-analysis. **Pediatrics,** v. 137, n. 1, p. 1-16, Jan. 2016.

BRASIL. Ministério da Saúde**.** Secretaria de Atenção à Saúde. Departamento de Ações Programáticas Estratégicas. **Atenção humanizada ao recém-nascido de baixo peso:** Método Canguru – manual técnico. Brasília: Ministério da Saúde, 2017.

BRASIL. Ministério da Saúde. Secretaria de Atenção à Saúde. Departamento de Ações Programáticas Estratégicas. **Guia de orientações para o Método Canguru na atenção básica:** cuidado compartilhado. Brasília: Ministério da Saúde, 2016.

BRASIL. Ministério da Saúde. Secretaria de Atenção à Saúde**. Protocolo de atenção à saúde e resposta à ocorrência de microcefalia relacionada à infecção pelo vírus Zika.** Brasília: Ministério da Saúde, 2016.

BRASIL. Ministério da Saúde. Secretaria de Atenção à Saúde. Departamento de Ações Programáticas Estratégicas. Departamento de Atenção Básica. **Aleitamento materno, distribuição de leites e fórmulas infantis em estabelecimentos de saúde e a legislação**. Brasília : Ministério da Saúde, 2014.

BRASIL. Ministério da Saúde. Secretaria de Atenção à Saúde**.** Departamento de Ações Programáticas Estratégicas. **Atenção humanizada ao recém-nascido de baixo peso:** Método Canguru – Manual Técnico. Brasília: Ministério da Saúde, 2013.

BRASIL. Ministério da Saúde. Secretaria de Atenção à Saúde. Departamento de Ações Programáticas Estratégicas. **Atenção à saúde do recém-nascido:** guia para os profissionais de saúde. Cuidados com o recém nascido pré-termo. Brasília: Ministério da Saúde, 2011a.

BRASIL. Ministério da Saúde. Secretaria de Atenção à Saúde. Departamento de Ações Programáticas Estratégicas. **Atenção à saúde do recém-nascido:** guia para os profissionais de saúde. Problemas respiratórios, cardiocirculatórios, metabólicos, neurológicos, ortopédicos e dermatológicos. Brasília: Ministério da Saúde, 2011b.

BRASIL. Ministério da Saúde. **Saúde da Criança: nutrição infantil e, aleitamento materno e alimentação complementar.** Caderno de Atenção Básica, nº 23. Brasília, DF: 2009.

BRAZELTON, T.B.; PARKER, W.B.; ZUCKERMAN, B. **Importance of behavioral assessment of the neonate**. Curr. Probl. Pediatr. 1976, 7, 1–82.

BUSQUET-VANDERHEYDEN, M. **O bebê em suas mãos:** método das cadeias fisiológicas. 1. ed. Barueri : Manole, 2009.

CABRAL, L. A., SCHETTINO, R. C., POMPEU, L. P. Estratégias favorecedoras do desenvolvimento neuropsicomotor de recém-nascidos pré-termo: da UTI ao ambulatório de seguimento. *In*: ASSOCIAÇÃO BRASILEIRA DE FISIOTERAPIA CARDIORRESPIRATÓRIA E FISIOTERAPIA EM TERAPIA INTENSIVA; MARTINS, J. A., NICOLAU, C. M., ANDRADE, L. B. (Org.). **PROFISIO Programa de atualização em fisioterapia pediátrica e neonatal:** cardiorrespiratória e terapia intensiva. Ciclo 4. Porto Alegre: Artmed/Panamericana, 2015. v.1, p.95-127.

CALDAS, I. F. R. Desenvolvimento sociocomunicativo: interação mãe-bebê e fatores de risco. *In*: ASSOCIAÇÃO BRASILEIRA DE FISIOTERAPIA CARDIORRESPIRATÓRIA E FISIOTERAPIA EM TERAPIA INTENSIVA; MARTINS, J. A., SCHIVINSKI, C. I. S., RIBEIRO, S. N. S. (Org.). **PROFISIO Programa de atualização em fisioterapia pediátrica**

e neonatal: cardiorrespiratória e terapia intensiva. Ciclo 6. Porto Alegre: Artmed/Panamericana, 2017. v.3, p. 29-50.

CALDAS, I. F. R. Fatores de risco e desenvolvimento sociocomunicativo em prematuros. **Revista Psicologia:** Teoria e Prática, São Paulo, v. 18, n. 2, p. 129-141, Maio/Ago. 2016.

CARBAJAL, R.; LENCLEN, R.; JUCIE, M.; PAUPE, A.; BARTON, B. A., ANAND, K. J. Morphine does not provide adequate analgesia for acute procedural pain among preterm neonates. **Pediatrics**, v.115, n. 6, p.1494-500, Mar. 2005.

CARVALHO, M. G. S., SIQUEIRA, J. C. F. Estimulação suplementar para recém-nascidos de alto risco. *In*: ASSOCIAÇÃO BRASILEIRA DE FISIOTERAPIA CARDIORRESPIRATÓRIA E FISIOTERAPIA EM TERAPIA INTENSIVA; NICOLAU, C. M., ANDRADE, L.B. (Org.). **PROFISIO Programa de atualização em fisioterapia pediátrica e neonatal:** cardiorrespiratória e terapia intensiva. Ciclo 2. Porto Alegre: Artmed/Panamericana, 2013. v. 3, p. 117-153.

DAWSON, G.; JONES, E. J. H.; MERKLE, K.; VENEMA, K.; LOWY, R.; FAJA, S.; KAMARA, D.; MURIAS, M.; GREENSON, J.; WINTER, J.; SMITH, M.; ROGERS, S. J. ; WEBB, S. J. Early Behavioral Intervention Is Associated With Normalized Brain Activity in Young Children With Autis. **Journal American Academy Child of Adolescent Psychiatry**, v. 51, n. 11, p.1150-1159, Nov. 2012.

DE GROOT, L. Posture and mobility in preterm infants. **Developmental Medicine e Child Neurology**, v. 42, n. 1, p. 65-8, Jan. 2000.

DE SCHUYMER, L.; DE GROOTE, I.; STRIANO, T.; STHAL, D.; ROYERES, H. Dyadic and triadic skills in preterm and full term infants: a longitudinal study in the first year. **Infant Behavior Development**., v. 34, p. 179-88, Feb. 2011.

DUARTE, D. T. R.; VANZO, L. C.; COPPO, M. R. C.; STOPIGLIA, M. S. Estimulação sensório-motora no recém-nascido. *In*: SARMENTO, G. J. V.; DE CARVALHO, F. A.; PEIXE, A. A. F. (Org.). **Fisioterapia Respiratória em Pediatria e Neonatologia**. 2. ed. Barueri: Manole, 2011.

DUBOWITZ, L. M.; DUBOWITZ, V.; GOLDERG, C. Clinical Assessment of gestational age in the newborn infant. **Journal Pediatrics**, v. 77, n. 1, p. 1-10, July 1970.

EINSPIELER, C.; PRECHT, H. F. R.; FERRARI, F.; CIONI, G.; BOS, A. F. The qualitative assessment of general movements in preterm, term and young infants - review of the methodology. **Early Human Development,** v. 50, n.1, p. 47-60, Nov. 1997.

EINSPIELER, C.; PEHARZ, R.; MARSCHIK, P. B. Fidgety movements – tiny in appearance, but huge in impact. **Jornal Pediatria.** v. 92, n. 3, supl. 1, p. 64-70, May/June 2016.

EINSPIELER, C.; PRECHTL, H. F. R. Prechtl's assessment of general movements: a diagnostic tool for the functional assessment of the young nervous system. **Mental Retardation and Developmental Disabilites Research Reviews**., v. 11, n. 1, p. 61-67, Apr. 2005.

FELDMAN, R., ROSENTHAL, Z., EIDELMAN, A. I. Maternal-preterm skin-to-skin contact enhances child physiologic organization and cognitive control across the first 10 years of life. **Biological Psychiatry,** v. 75, n. 1, p. 56-64, Jan. 2014.

FERREIRA, H.C., SANTOS, R.S. Posição prona em pediatria e neonatologia. *In*:ASSOCIAÇÃO BRASILEIRA DE FISIOTERAPIA CARDIORRESPIRATÓRIA E FISIOTERAPIA EM TERAPIA INTENSIVA; MARTINS, J. A., NICOLAU, C. M., ANDRADE, L. B. (Org.). **PROFISIO Programa de atualização em fisioterapia pediátrica e neonatal:** cardiorrespiratória e terapia intensiva. Ciclo 5. Porto Alegre: Artmed/Panamericana, 2016. v. 2, p. 9-57.

FOHE, K., KROPF, S., AVENARIUS, S. Skin-to-skin contact improves gas exchange in premature infants. **Journal Perinatology**, v. 5, n. 5, p. 311-5, July/Aug. 2000.

FORMIGA C. K. M. R.; PEDRAZZANI, E. S.; TUDELLA, E. **Intervenção Precoce com Bebês de Risco**. São Paulo: Atheneu, 2010.

FORMIGA, C. K. M. R.; LINHARES, M. B. M. Avaliação do desenvolvimento inicial de crianças nascidas pré-termo. **Revista da Escola de Enfermagem da USP**, v. 43, n. 2, p. 472-480, jun. 2009.

FORMIGA, C. K. M. R.; PEDRAZZANI, E. S.; TUDELLA, E. Desenvolvimento motor de lactentes pré-termo participantes de um programa de intervenção fisioterapêutica precoce. **Revista Brasileira de Fisioterapia,** v. 8, n. 3, p. 239-245, set./dez. 2004.

FORMIGA, C. K. M. R.; TUDELLA, E; MARQUES, L. R. FAGUNDES, R. R., DO AMARAL, L. E. F.; LINHARES, M. B. M. Desenvolvimento motor de bebês pré-termo e a termo de 0 a 6 meses de idade. **Pediatria Moderna**, v.51, n.12, p. 422-426, dez. 2015.

FORMIGA, C. K. M. R.; CEZAR, M. E. N.; LINHARES, M. B. M. Avaliação longitudinal do desenvolvimento motor e da habilidade de sentar em crianças nascidas prematuras. **Fisioterapia e Pesquisa**, v. 17, n. 2, p. 102-107, jun. 2010.

FRIEDRICH, L., CORSO, A. L., JONES, M. H. Prognóstico pulmonar em prematuros. **Jornal de Pediatria,** v. 81, Supl 1, p. S79-S88, 2005.

FUENTEFRIA, R. N.; SILVEIRA, R.C.; PROCIANOY, R.S. Motor development of preterm infants assessed by the Alberta Infant Motor Scale: systematic review article. **Jornal de Pediatria**, v. 93, n. 4, p. 328-342, July/Aug. 2017.

GARCIA, C.; GEPHART, S. M. The effectiveness of early intervention programs for NICU graduates. **Advances in Neonatal Care**, v.13, n. 4, p. 272-8, Aug. 2013.

GARCIA, J. M.; GHERPELLI, J. L.; LEONE, C. R. The role of spontaneous general movement assessment in the neurological outcome of cerebral lesions in preterm infants. **Jornal de Pediatria**, v. 80, n. 4, p. 296-304, July/Aug. 2004.

GASPARDO, M. C., LINHARES, M. B. M., MARTINEZ, F. E. A eficácia da sacarose no alívio de dor em neonatos: revisão sistemática da literatura. **Jornal de Pediatria**, v. 81, n. 6, p. 435-442, Nov. 2005.

GONÇALVES, M. C. P. Practicality and effectiveness of the physical examination protocol for neonatal neuromotor scanning. **Fiep Bulletin.** Special Edition- Article II, v. 80, p. 431-35, 2010.

GONÇALVES, M. C. P. **Prematuridade:** desenvolvimento neurológico e motor: avaliação e tratamento. Rio de Janeiro: Revinter, 2012.

GRENIER, I. R.; BIGSBY, R.; VERGARA, E. R.; LESTER, B. M. Comparison of motor self-regulatory and stress behaviors of preterm infants across body positions. **The American Journal of Occupational Therapy**, v. 57, n. 3, p. 289-97, May/June. 2003.

GRUNAU, R. Early pain in preterm infants: a model of long-term effects. **Clinics in Perinatology**, v.29, n. 3, p. 373-394, Oct. 2002.

GRYBOSKI, J. D. Suck and swallow in the premature infant. **Pediatrics**, v. 43, n. 1, p. 96-102, Jan. 1969.

GUIHARD-COSTA, A. M.; LARROCHE, J. C. Differential growth between the fetal brain and its infratentorial part. **Early Human Development**, v. 23, n. 1, p. 27-40, June 1990.

GUINCHAT. V.; THORSEN, P.; LAURENT, C.; CANS, C.; BODEAU, N.; COHEN, D. Pre, peri- and neonatal risk factors for autism**. Acta Obstetriciaet et Gynecologica Scandinavica,** v. 91, n. 3, p. 287-300, Mar. 2012.

HADDERS-ALGRA, M. Early diagnosis and early intervention in cerebral palsy**. Frontiers Neurology**, v. 5, n. 185, p. 1-13, Sept. 2014.

HAGBERG, H., JACOBSSON, B. Brain injury in preterm infants - what can the obstetrician do? **Early Human Development**, v. 81, n. 3, p. 231–235, Mar. 2005.

HEATHCOCK, J. C.; LOBO, M.; GALLOWAY, J. C. Movement training advances the emergence of reaching in infants born at less than 33 weeks of gestational age. **Physical Therapy**, v. 88, n. 3, p. 310–322, Mar. 2008.

HUNT, F. The importance of kangaroo care on infant oxygen saturation levels and bonding. **Journal of Neonatal Nursing,** v.14, n. 2, p. 47-51, Apr. 2008.

JOHNSTON, C.C. Kangaroo mother care diminishes pain from heel lance in very preterm neonates: a crossover trial. **BMC Pediatrics**, v. 8, n. 13, p. -9, Apr. 2008.

KINNEY, H.C. The near-term (late preterm) human brain and risk for periventricular leukomalacia: A review. **Seminars in Perinatology**, v. 30, n. 2, p. 81-88, Apr. 2006.

LAGO, P.; GARETTI, E.; MERAZZI, D.; PIERAGOSTINI, L.; ÂNCORA, G. PIRELLI, A.; BELLIENI, C. V. Pain Study Group of the Italian Society of Neonatology. Guideline for procedural pain in the newborn. **Acta Paediatrica**., v. 98, n. 6, p. 932-939, June 2009.

LAMY-FILHO, F.; DA SILVA, A. A. M.; LAMY, Z. C.; GOMES, M. A. S. M.; MOREIRA, M. E. L.; GRUPO DE AVALIAÇÃO DO MÉTODO CANGURU; REDE BRASILEIRA DE PESQUISAS NEONATAIS . Evaluation of the neonatal outcomes of the kangaroo mother method in Brazil. **The Journal of Pediatrics**, v.84, n. 5, p. 428-435, Sept./Oct. 2008.

LEAL, M. C.; ESTEVES-PEREIRA, A. P.; NAKAMURA-PEREIRA, M.; TORRES, J. A; TEMA-FILHA, M.; DOMINGUES, R. M. S. M.; DIAS, M. A. B.; MOREIRA, M. E.; GAMA, S. G. Prevalence and risk factors related to preterm birth in Brazil. **Reproductive Health**, v.13, Suppl 3, n. 127, p. 163-174, Oct. 2016.

LEMOS, A.; MAUX, D. A. S. X.; PAIVA, G. S. Assistência ventilatória em patologias neonatais. *In*: ASSOCIAÇÃO BRASILEIRA DE FISIOTERAPIA CARDIORRESPIRATÓRIA E FISIOTERAPIA EM TERAPIA INTENSIVA; NICOLAU, C. M.; ANDRADE, L. B. (Org.). **PROFISIO Programa de atualização em fisioterapia pediátrica e neonatal:** cardiorrespiratória e terapia intensiva. Ciclo 2. Porto Alegre: Artmed/Panamericana, 2013. v.3, p. 47-87.

LIEM, K. D.; GREISEN, G. Monitoring of cerebral haemodynamics in newborn infants. **Early Human Development**., v. 86, n. 3, p. 155-8, Mar. 2010.

LIMA, M. P. Bases do Método Reequilíbrio Tóraco-abdominal. *In*: SARMENTO, G. J. V. (Org.). **O ABC da Fisioterapia Respiratória**. Barueri: Manole, 2009. p. 197-211.

LUDINGTON-HOE, S. M.; ANDERSON, G. C.; SIMPSON, S.; HOLLINGSEAD, A.; ARGOTE, L. A.; REY, H. Birth-related fatigue in 34-36-week preterm neonates: rapid recove y with very early kangaroo (Skin-to-Skin) care. **Journal Obstetric Gynecologic and Neonatal Nursing**, v. 28, n. 1, p. 94-103, Jan./Feb. 1999.

LUDINGTON-HOE, S. M, FERREIRA,C.N, GOLDSTEIN,M.R. Kangaroo care with a ventilated preterm infant. **Acta Paediatrica**, v. 87, n. 6, p. 711-3, Jun. 1998.

MARLOW, N. Neurocognitive outcome after very preterm birth. **Archives Disease Childhood Fetal and Neonatal**, v. 89, n. 3, p. 224-8, May 2004.

NEWNHAM, C. A., INDER, T. E., MILGROM, J. Measuring preterm cumulative stressors within the NICU: The neonatal infant stressor scale. **Early Human Development**, v. 85, n. 9, p. 549-555, Sept. 2009.

NOVAK, I.; MORGAN, C.; ADDE, L.; LACKMAN, J.; BOYD, R.N.; BRUNSTROM-HERNANDEZ, J.; CIONI, G.; DAMIANO, D.; DARRAH, J.; ELIASSON, A.; DE VRIES, L. S.; EINSPIELER, C.; FAHEY, M.; FEHLINGS, D.; FERRIERO, D. M.; FETTERS, L.; FIORI, S.; FORSSBERG, H.; GORDON, A. M.; GREAVES, S.; GUZZETA, A. HADDERS-ALGRA, M.; HARBOURNE, R. KAKOOZA-MWESIGE, A.; KARLSSON, P.; KRUMLINDE-SUNDHOLM, L.; LATAL, B.; LOUGHRAN-FOWLDS, A.; MAITRE, N.; MCINTYRE, S.; NORITZ, G.; PENNINGTON, L.; ROMEO, D. M.; SHEPHERD, R.; SPITTLE, A. J.; THORNTON, M.; VALENTINE, J.; WALKER, K.; WHITE, R.; BADAWI, N. Early, Accurate Diagnosis and Early Intervention in Cerebral Palsy: Advances in Diagnosis and Treatment. **JAMA Pediatrics**, v. 171, n. 9, p. 897-907, Sept. 2017.

OBERG, G.K.; CAMPBELL, S. K.; GIROLAMI, G. L.; USTAD, T.; JORGENSEN, L.; KAARESEN, P. I. Study protocol: an early intervention program to improve motor outcome in preterm infants: a randomized controlled trial and a qualitative study of physiotherapy performance and parental experiences. **BMC Pediatrics,** v. 15, n. 12, p. 1-9, Feb. 2012.

ODD, D.; EVANS, D.; EMOND, A. Preterm birth, age at school entry and educational performance. **PLoS One,** v. 8, n. 10, Oct. 2013.

PADILHA, H. F.; STEIDL, E. M. S.; BRAZ, M. M. Efeitos do método mãe-canguru em recém-nascidos pré-termo. **Fisioterapia Brasil**, v. 15, n. 2, Mar./Abr. 2014.

PIN, T.W.; DARRER, T.; ELDRIDGE, B.; GALEA, M. P. Motor development from 4 to 8 months corrected age in infants born at or less than 29 weeks' gestation. **Developmental Medicine and Child Neurology,** v. 51, p. 739-45, Mar. 2009.

PIPER, M. C.; DARRAH. J. **Motor assessment of the developing infant**. Philadelphia: W. B. Saunders Company, 1994.

PITCHER, J. B.; SCHNEIDER, L.A.; BURNS, N. R.; DRYSDALE, J. L.; HIGGINS, R. D.; NETTELBECK, T. J.; HASLAN, R. R.; ROBINSON, J. S. Reduced corticomotor excitability and motor skills development in children born preterm. **The Journal of Physiology,** v. 590, n. 22, p. 5827-5844, Nov. 2012.

PRECHTL, H. F. Qualitative changes of spontaneous movements in fetus and preterm infant are a marker of neurological dysfunction. **Early Human Development**, v. 23, n. 3, p. 151-158, Sept. 1990.

RAMACHANDRAN, S.; DUTTA, S. Early developmental care interventions of preterm very low birth weight infants. **Indian Pediatrics**, v. 50, n. 8, p.765-770, Aug. 2013.

REICHERT, A. P. S.; LINS, R. N. P.; COLLET, N. Humanização do cuidado da UTI Neonatal. **Revista Eletrônica de Enfermagem**, v. 9, n. 1, p. 200-213, Jan./Fev. 2007.

REINAUX, C. M. A. **Evolução motora de recém-nascidos pré-termo submetidos ao método mãe-canguru.** 2005. 149 f. Dissertação (Mestrado em Fisioterapia) - Universidade Metodista de Piracicaba, Piracicaba, 2005.

SACCANI, R., VALENTINE, N. C. Reference curves for the Brazilian Alberta Infant Motor Scale: percentiles for clinical description and follow-up over time. **Jornal de Pediatria**, v. 88, n. 1, p. 40-47, Jan./Feb. 2012.

SACCANI, R.; VALENTINI, N. C.; PEREIRA, K. R. G. New Brazilian developmental curves and reference values for the Alberta infant motor scale. **Infant Behavior & Development**, v. 45, p. 38-46, Nov. 2016.

SANTORO JÚNIOR, W.; MARTINEZ, F. E. Effect of intervention on the rates of breastfeeding of very low birth weight newborns. **Jornal de Pediatria**, v. 83, n. 6, p. 541-546, Nov./Dez. 2007.

SANTOS, M. H.; AZEVEDO FILHO, F. M. Benefícios do método mãe canguru em recém-nascidos pré-termo ou baixo peso: uma revisão de literatura. **Universitas**: Ciências da Saúde, v.14, n. 1, p. 67-76, Jan./Jun. 2016.

SCHNEIDER, C.; CHARPAK, N.; RUIZ-PELAEZ, J.G.; TESSIER, R. Cerebral motor function in very premature-at-birth adolescents: a brain stimulation exploration of kangaroo mother care effects. **Acta Paediatrica**, v. 101, n. 10, p. 1045-1053, Oct. 2012.

SHAH, P. E.; ROBBINS, N.; COELHO, R. B.; POEHIMANN, J. The paradox of prematurity: the behavioral vulnerability of late preterm infants and the cognitive susceptibility of very preterm infants at 36 months post-term. **Infant Behavior & Development,** v. 36, n. 1, p. 50-62, Feb. 2013.

SHAIKH, A. G.; NAMRATA, P. Effectiveness of Massage Therapy as an Adjunct to Kangaroo Mother Care on Physiological and Behavioural Status of Low Birth Weight Preterm Infants. **Indian Journal of Physiotherapy and Occupational Therapy**, v. 11, n. 2, p. 103-8, Apr./June 2017.

SHAREK, P. J.; POWERS, R.; KOEHN, A.; ANAND, K. J. Evaluation and development of potentially better practices to improve pain management of neonates. **Pediatrics,** v. 118, Suppl 2, p. 78-86, Nov. 2006.

SHONKOFF, J. P.; GARNER, A. S. American Academy of Pediatrics Technical Report. The lifelong effects of early childhood adversity and toxic stress. **Pediatrics**, v. 129, n. 1, p. 232-244, Jan. 2012.

SILVA, E. S.; NUNES, M. L. The influence of gestational age and birth weight in the clinical assessment of the muscle tone of healthy term and preterm newborns. **Arquivos de Neuropsiquiatria**, v. 63, n. 4, p. 956-962, Dez. 2005.

SILVERMAN, W. A.; ANDERSEN, D. H. A controlled clinical trial of effects of water mist on obstructive respiratory signs, death rate and necropsy findings among premature infants. **Pediatrics**, v. 17, n. 1, p. 1–10, Jan. 1956.

SIMONS, S.H.; VAN DIJK, M.; ANAND, K. S.; ROOFTHOOFT, D; VAN LINGEN, R. A.; TIBBOEL, D. Do we still hurt newborn babies? A prospective study of procedural pain and analgesia in neonates. **Archives Pediatrics Adolescent Medicine**., v. 157, n. 11, p. 1058-64, Nov. 2003.

SISTEMA DE INFORMAÇÕES DE NASCIDOS VIVOS (SINASC). **Estatísticas vitais,** 2017. Disponível em: <http://tabnet.datasus.gov.br/cgi/deftohtm.exe?_sinasc/cnv/nvms.def>. Acesso em: 31 out. 2017.

SOARES-MARANGONI, D. A.; TEDESCO, N. M.; NASCIMENTO, A. L.; DE ALMEIDA, P. R.; PEREIRA, C. N. S. General movements and motor outcomes in two infants exposed to Zika virs: brief report. **Developmental Neurorehabilitation**, 2018. DOI: 10.1080/17518423.2018.1437843

SOARES, D. A.; VON HOFSTEN, C.; TUDELLA, E. Development of exploratory behavior in late preterm infants. **Infant Behavior and Development,** v. 35, n. 4, p. 912–915, Dec. 2012.

SOARES, D. A.; CUNHA, A. C.; TUDELLA, E. Differences between late preterm and full-term infants: Comparing effects of a short bout of practice on early reaching behavior. **Research in Developmental Disabilities**, v. 35, n. 11, p. 3096-3107, Nov. 2014.

SOUKKA, H.; GRONROOS, L.; LEPPASALO, J.; LEHTONEN. L. The effects of skin-to skin care on the diaphragmatic electrical activity in preterm infants. **Early Human Development**, v. 90, n. 9, p. 531-534, Sept. 2014.

SPITTLE, A.; ORTON, J.; ANDERSON, P.; BOYD, R.; DOYLE, L.W. Early developmental intervention programmes post-hospital discharge to prevent motor and cognitive impairments in preterm infants. **Cochrane Database Systematic Reviews,** n. 12, p. 1-103, Oct. 2012.

SPITTLE, A.; ORTON, J.; ANDERSON, P.; BOYD, R.; DOYLE, L.W. Early developmental intervention programmes provided post hospital discharge to prevent motor and cognitive impairment in preterm infants. **Cochrane Database Systematic Reviews**, n.11, p. 1-110, 2015.

VALENTINI, N. C; SACCANI, R. Brazilian Validation of the Alberta Infant Motor Scale. **Physical Therapy**, v. 92, n. 3, p. 440-7, Mar. 2012.

VANDERVEEN, J. A.; BASSLER, D.; ROBERTSON, C. M. T.; KIRPALANI, H. Early interventions involving parents to improve neurodevelopmental outcomes of premature infants: a meta-analysis. **Journal Perinatology**, v. 29, n. 5, p. 343-351, May 2009.

VAIVRE-DOURET, L.; ENNOURI, K.; JRAD, I.; GARREC, C.; PAPIERNIK, E. Effect of positioning on the incidence of abnormalities of muscle tone in low-risk, preterm infants. **European Journal of Paediatric Neurology**, v. 8, n. 1, p. 21-34, Jan. 2004.

VENTURELLA, C. B; ZANANDREA, G.; SACCANI, R.; VALENTINI, N. C. Desenvolvimento motor de crianças entre 0 e 18 meses de idade: Diferenças entre os sexos. **Motricidade**, v. 9, n. 2, p. 3-12, Abr. 2013.

WORLD HEALTH ORGANIZATION (WHO). Library Cataloguing in Publication Data. **International statistical classification of diseases and related health problems**. - 10th rev., v. 2, WAO, 2010.
